# Switching Site Selectivity in Alkoxyamine Hydration: From Lone-Pair Direction to Solvent Network Dominance

**DOI:** 10.1021/jacs.6c08250

**Published:** 2026-07-07

**Authors:** Filippo Baroncelli, Luca Evangelisti, Juan Carlos López, Susana Blanco, Assimo Maris, Sonia Melandri

**Affiliations:** † Department of Chemistry G. Ciamician, 9296 University of Bologna, I-40129 Bologna, Italy; ‡ Interdepartmental Centre for Industrial Aerospace Research, University of Bologna, I-47121 Forlì, Italy; § Department of Physical Chemistry and Inorganic Chemistry, IU-CINQUIMA, 16782 University of Valladolid, E-47011 Valladolid, Spain

## Abstract

Predicting site selectivity during the earliest stages of solvation in multifunctional organic molecules remains a complex challenge in chemical physics. In this work, we use high-resolution rotational spectroscopy and quantum-chemical calculations to decode the hydration landscape of *N*,*N*-diethylacetyloxyamine (DEAcA), a molecule featuring competing sp^3^ nitrogen and carbonyl oxygen acceptors. While the isolated monomer preferentially adopts a *syn* configuration driven by the electric dipole moment minimization and the synergistic alleviation of oxygen–oxygen electrostatic repulsion, microsolvation ((H_2_O)_*n*_, *n* = 1–3) reveals an exclusive preference for the highly basic nitrogen site. We demonstrate that the ^14^N nuclear quadrupole coupling (NQC) constants provide direct experimental evidence of the cooperative polarization that strengthens the nascent water chain. Crucially, at the tetrahydrated level (*n* = 4), we identify a sharp transition in the solvation regime: the global minimum shifts to a cyclic water tetramer anchored at the carbonyl oxygen. This structural pivot marks the boundary where localized stereoelectronic control yields to the collective stabilization of the solvent network. These findings provide a rigorous benchmark for modeling the transition from molecular recognition to bulk-like solvation in complex organic systems.

## Graphical abstract

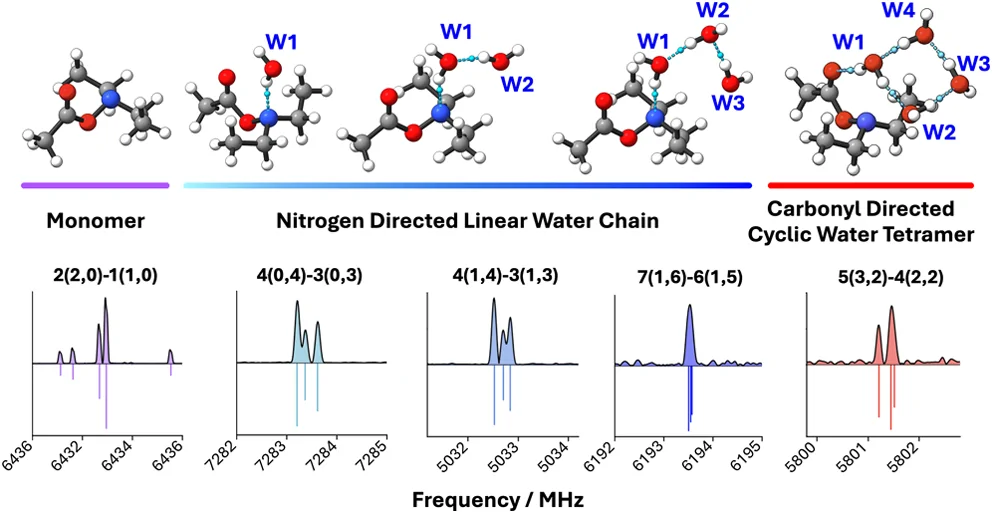

## Introduction

Alkoxyamines represent an important class of molecules whose utility spans from synthetic intermediates to advanced materials science.^1,2^ Their chemical significance arises primarily from the unique properties of the N–O bond, which undergoes reversible homolytic cleavage to generate stable nitroxide radicals.^3^ This reactivity is the cornerstone of nitroxide-mediated polymerization (NMP), enabling the synthesis of complex macromolecular architectures with precise stereochemistry and low dispersity.^4,5^ Furthermore, these species are essential participants in the Denisov cycle, acting as hindered amine light stabilizers (HALSs) to prevent polymer photodegradation.^6,7^ However, translating these macroscopic applications into predictive models requires a precise atomistic understanding of their fundamental reactivity, preferred conformations, and initial interactions with the solvent molecules.

A recurring challenge in chemical physics is predicting site selectivity in multifunctional acceptors at the earliest stages of solvation. In molecules containing both oxygen and sp^2^-hybridized nitrogen (e.g., oxazoles,^8^ oxime ethers,^8^ and aza-crowns^9^), hydrogen bonding preferentially occurs at the nitrogen. Conversely, in aminoketones and aminoesters, the carbonyl oxygen is typically the favored acceptor due to resonance effects, unless conjugation is sterically disrupted (e.g., twisted amides).^10^ While gas-phase solvation studies of 2-pyridone,^11^ formamide,^12^ and 2′-aminoacetophenone^13^ confirm this carbonyl preference, the introduction of saturated, sp^3^-hybridized centers, such as those in ethanolamine^14^ and *N*,*N*-diethylhydroxylamine (DEHA^15^), can shift this preference back to the nitrogen.

Within this competitive landscape, *N*,*N*-diethylacetyloxyamine (DEAcA hereafter, Figure 1) presents an intriguing structural puzzle. It possesses three distinct proton-accepting sites, an sp^3^ nitrogen, an sp^3^ ether-like oxygen, and an sp^2^ carbonyl oxygen, all within a flexible scaffold lacking stabilizing conjugation. Furthermore, the orientation of the acetyl group in acetates is traditionally dictated by the nature of the substituent to ensure optimal electric dipole alignment (see below); DEAcA tests whether these conformational “rules” hold when a bulky, highly basic amine is introduced. In this work, we characterize the structural landscape of DEAcA and its progressive hydration using high-resolution rotational spectroscopy in combination with quantum-chemical calculations. Rotational spectroscopy probes the quantized rotation of molecules in the gas phase, yielding rotational constants that are directly related to the molecular moments of inertia and, therefore, to the three-dimensional geometry of the species with subångström precision. When coupled with supersonic jet expansion, each observed spectrum serves as a unique fingerprint, allowing unambiguous identification of distinct conformers as well as weakly bound complexes trapped in the cold, isolated conditions of the expansion. When a nucleus carries a nonzero electric quadrupole moment, such as ^14^N (*I* = 1), its coupling to the electric field gradient (EFG) at that nuclear site produces characteristic hyperfine splittings in the rotational spectrum. The nuclear quadrupole coupling (NQC) constants extracted from these splittings reflect the asymmetry of the electron density around the nucleus, making them uniquely sensitive probes of hybridization state, lone-pair directionality, and changes in the local electronic environment induced by hydrogen bonding or conformational change.^16^ By tracking the ^14^N NQC constants and planar moments of inertia, we demonstrate how local basicity dictates a lone-pair-directed cooperative water chain up to the third hydration step (*n* = 1–3). Extending this analysis to the tetrahydrated complex, we identify a structural regime shift, delineating the boundary at which local electronic control gives way to collective solvent–solvent interactions.

**Figure 1:**
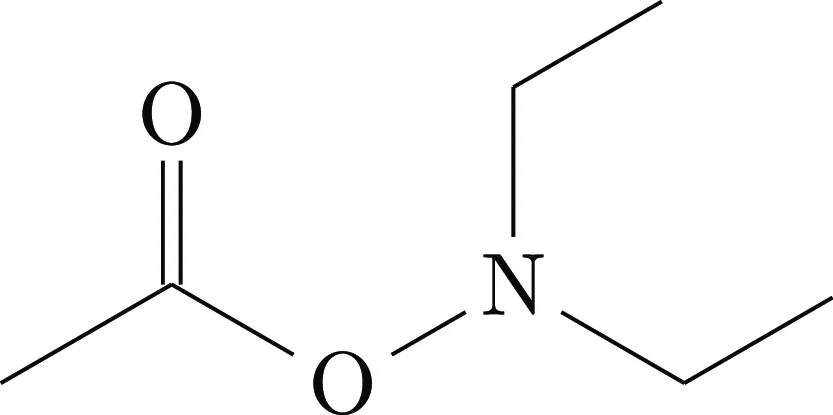
*N*,*N*-Diethylacetyloxyamine, C_6_H_13_NO_2_, DEAcA for short.

## Methods and Materials

### Computational Procedures

Geometry optimizations and vibrational frequency calculations of the most stable conformers of DEAcA were performed at the MP2/aug-cc-pVTZ level of theory^17,18^ using the Gaussian 16 (Rev. A.03) software package.[Fn fna] For the water complexes, preliminary conformational searches were conducted using the Conformer-Rotamer Ensemble Sampling Tool (CREST^19^). The resulting structures were refined via density functional theory (DFT) at the B3LYP-D3­(BJ)/def2-TZVP level of theory,^20–24^ including vibrational analyses to verify them as true local minima. The lowest-energy geometries were subsequently reoptimized at the MP2/aug-cc-pVTZ level. Finally, the ab initio electron density distribution was analyzed utilizing the quantum theory of atoms in molecules (QTAIM^25^) and noncovalent interaction plotting^26^ within the Multiwfn program.^27^ The theoretical results are described in detail in the Supporting Information.

### Synthesis and Sample Preparation

DEAcA was synthesized via the direct acylation of DEHA with acetyl chloride according to established protocols:^28^

1 $${\left(C_{2}H_{5}\right)}_{2}\mathrm{NOH}+{\mathrm{CH}}_{3}C\left(O\right)\mathrm{Cl}\overset{N{\left({\mathrm{CH}}_{3}\right)}_{3}}{\underset{{\mathrm{CH}}_{2}{\mathrm{Cl}}_{2}}{\to }}{\left(C_{2}H_{5}\right)}_{2}\mathrm{NOC}\left(O\right){\mathrm{CH}}_{3}+\mathrm{HCl}$$

The resulting pale-yellow liquid is thermally sensitive; to mitigate decomposition, samples were stored at 253 K and used immediately. For spectroscopic analysis, the vapor pressure of the liquid at room temperature was entrained in a stream of noble carrier gas (He, Ne, or Ar) and expanded through a pinhole nozzle into a vacuum chamber. This supersonic expansion effectively cooled the molecular internal degrees of freedom to rotational temperatures of *T*_rot_ = 1–10 K, both simplifying the rotational spectrum and stabilizing weakly bound molecular complexes.

### Instrumentation

Three complementary spectrometers were employed to ensure complete characterization. A chirped-pulse pulsed-jet Fourier-transform microwave spectrometer was used for rapid broadband surveys (CP-PJ-FTMW, 2–8 GHz).^29,30^ A COBRA-type pulsed-jet Fourier-transform microwave spectrometer provided high-sensitivity and high-resolution measurements of hyperfine structures (PJ-FTMW, 6.5–18.5 GHz).^31–34^ A Stark-modulated free-jet absorption millimeter-wave spectrometer extended the observations into the high-frequency regime (FJ-AMMW, 59.6–74.4 GHz).^35–37^ Experimental conditions are detailed in the Supporting Information.

### Spectral Analysis

Rotational transitions were assigned and fitted using the CALPGM program suite.^38^ The experimental spectra were modeled using a semirigid Watson S-reduction Hamiltonian^39^ in the I^r^ or ${\mathrm{III}}^{l}$ representation, depending on whether the investigated system was prolate or oblate:

2 $$Ĥ={Ĥ}_{R}+{Ĥ}_{\mathrm{CD}}+{Ĥ}_{Q}$$

where *Ĥ*_R_, *Ĥ*_CD_, and *Ĥ*_Q_ represent the rigid rotor, quartic centrifugal distortion, and ^14^N nuclear quadrupole interaction terms, respectively. The complete lists of assigned lines and determined spectroscopic constants are given in the Supporting Information.

## Results and Discussion

### Conformational Landscape

The potential energy surface of isolated DEAcA is governed by four rotatable bonds and the amine inversion coordinate. Of the 25 nonequivalent conformers identified computationally, six lie within 8 kJ mol^–1^ of the global minimum at the B3LYP-D3­(BJ)/def2-TZVP level of calculation.^28^ Structural refinements at the MP2/aug-cc-pVTZ level yield slightly shifted relative energies (Figure 2), yet two structural motifs consistently emerge as energetically dominant: (i) with respect to torsion about the N–O bond, the acetyl group preferentially orients toward the nitrogen lone pair, and (ii) considering the torsion around the O–C bond, the O=C–O–N frame adopts a *syn* configuration. Furthermore, the ethyl chains favor an *anti* rather than *gauche* arrangement, echoing the structural preferences previously observed in DEHA.^40^ While a comprehensive four-letter code can capture these geometrical nuances (denoting the CCNC chains, acetoxy orientation, and OCON acetyl arrangements; see Figure 2 and Section S2), we adopt a simplified, sequential numbering scheme based on increasing relative energy for clarity. A prime denotes conformers where the methyl group, rather than the carbonyl, faces the nitrogen lone pair.

**Figure 2:**
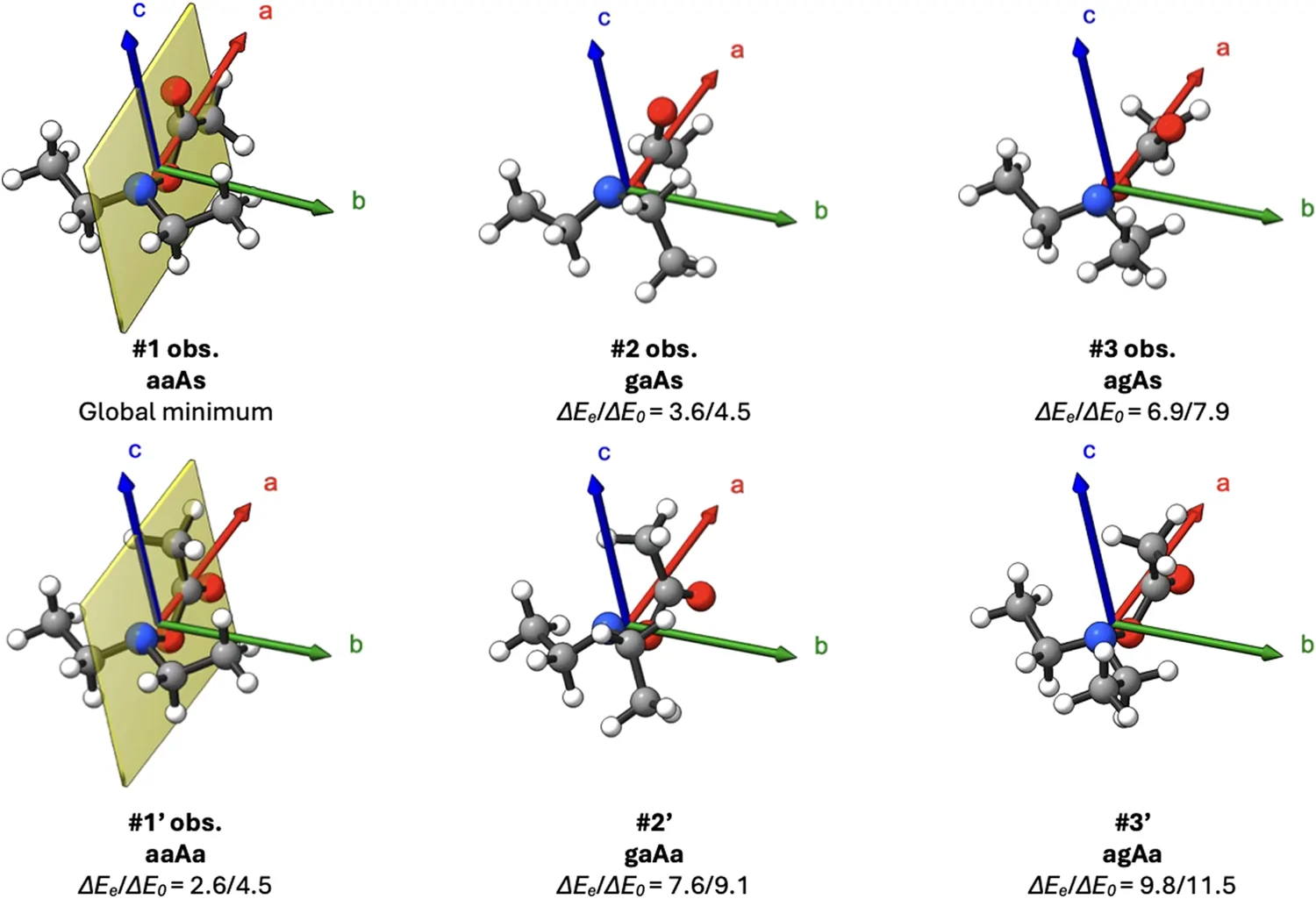
Geometries, principal axis systems (*a*, *b*, *c*), and relative energies (kJ mol^–1^) of the six most stable conformers of *N*,*N*-diethylacetyloxyamine at the MP2/aug-cc-pVTZ level of calculation. For structures possessing a plane of symmetry (*C*_*s*_ point group), the mirror plane (shaded) is explicitly rendered. The four-letter code describes the arrangement of the ethyl chains (CCNC), the acetoxy group with respect to the bisector line of the CNC bond angle and of the acetyl group (OCON), respectively. Species observed in the spectrum are indicated (obs.).

The rotational transitions for the four most stable conformers (#1, #2, #3, and #1′) were assigned, allowing for the determination of the spectroscopic constants reported in Table 1. To evaluate the conformational distribution within the supersonic expansion, the experimental relative populations were estimated from the intensities of the CP-FTMW transitions in the helium expansion, yielding an approximate ratio of #1/#2/#3/#1′ = 1:0.14:0.06:0.03. These values contrast significantly with the theoretical populations predicted by Boltzmann statistics at room temperature. Based on MP2/aug-cc-pVTZ zero-point corrected energies and explicitly accounting for the statistical weight of equivalent species, the theoretical ratio is expected to be 1:0.33:0.16:0.09. While conformer #1 is the dominant species, the experimental populations of #2, #3, and #1′ are less than half of that predicted by Boltzmann statistics based on the theoretical energy values. This disparity, also observed in the study of DEHA,^40^ suggests partial collisional relaxation into the global minimum during the supersonic expansion, a process typically facilitated by low interconversion barriers.^41^ However, relaxed one-dimensional (1D) torsional scans around the C–N and O–C bonds (B3LYP-D3­(BJ)/def2-TZVP) yield relatively high barriers for the #2 → #1 (1494 cm^–1^), #3 → #1 (781 cm^–1^), and #1′ → #1 (3708 cm^–1^) pathways. We hypothesize that the true relaxation mechanism circumvents these 1D topological maxima through strong coupling with the large-amplitude inversion motion of the amine group. A comprehensive, multidimensional treatment of the potential energy surface would be required to capture these correlated dynamics accurately. As the present work focuses on high-resolution structural characterization, such an exhaustive dynamical study is deferred to future work, though the computed 1D potential energy surfaces are available in the Supporting Information.

**Table 1:** Spectroscopic Parameters for the Observed Conformers of *N*,*N*-Diethylacetyloxyamine and Their Water Complexes^a^

	#1	#1′	#2	#3	#1:1	#1:2	#1:3	#1:4
EXP								
*A*/MHz	1725.75657(8)	1682.8137(6)	2023.0637(9)	1889.7753(8)	1197.7232(4)	1167.9953(4)	999.48(2)	783.7900(8)
*B*/MHz	1445.78368(7)	1463.5815(6)	1182.4110(7)	1245.0114(7)	976.7703(4)	655.0142(4)	445.1019(4)	403.2605(3)
*C*/MHz	953.2627(2)	966.3570(4)	858.2621(7)	937.9919(6)	886.7824(4)	620.9658(3)	434.8648(4)	359.9288(4)
*D*_J_/kHz	1.0618(4)^b^	—	0.25(3)^c^	—	0.26(1)	0.118(6)	0.089(2)^d^	0.119(3)^e^
χ_*aa*_/MHz	4.635(2)	3.736(9)	5.446(4)	3.455(9)	6.469(4)	6.522(6)	6.43(4)	4.15(2)
χ_*bb*_/MHz	0.567(2)	0.524(9)	0.550(4)	–0.37(1)	0.442(5)	0.300(6)	–6.7(1)	0.05(1)
χ_*cc*_/MHz	–5.202(2)	–4.260(5)	–5.996(4)	–3.09(1)	–6.911(5)	–6.822(6)	0.3(1)	–4.19(1)
Trans. type	*a*,*c*	*a*,*c*	*a*,*b*,*c*	*a*,*c*	*a*,*c*	*a*,*c*	*a*	*a*,*c*
*N*_lines_	396	45	141	42	95	155	102	147
σ^f^/kHz	26	31	9	17	15	11	11	21
MP2								
*A*/MHz	1734.11	1699.30	2029.94	1904.60	1225.56	1200.92	1016.04	795.56
*B*/MHz	1457.83	1479.52	1194.84	1253.12	994.70	668.33	455.81	416.80
*C*/MHz	964.51	978.54	862.57	946.51	893.37	623.12	444.80	371.78
χ_*aa*_/MHz	4.50	3.62	5.30	3.29	6.34	6.38	6.27	4.0790
χ_*bb*_/MHz	0.49	0.44	0.49	–0.37	0.36	0.19	–5.46	0.03
χ_*cc*_/MHz	–4.99	–4.06	–5.79	–2.92	–6.70	–6.57	–0.81	–4.11
μ_*a*_/μ_*b*_/μ_*c*_/D	1/0/2.4	3.6/0/2.2	0.7/1.2/2.0	1.3/0.1/2.3	2.3/0/3.0	1.9/0.1/1.2	2.0/0.9/0.9	1.3/0.6/2.4

a The table reports experimental and theoretical (MP2/aug-cc-pVTZ) rotational constants (*A*, *B*, *C*), quartic centrifugal distortion constant (*D*_J_), ^14^N nuclear quadrupole coupling constants (*χ*_*gg*_ *g* = *a*, *b*, *c*), and electric dipole moment components (|*μ*_*g*_|). Experimental uncertainties in parentheses are given in units of the last digit.

b *D*_JK_ = −0.992(2), *D*_K_ = −0.034(8), *d*_1_ = 0.0106(4), *d*_2_ = 0.4321(3) kHz.

c *D*_JK_ = 1.04(6), *d*_1_ = −0.11(1), and *d*_2_ = −0.026(4) kHz.

d *D*_JK_ = 1.41(4), *d*_2_ = 0.018(1) kHz.

e *D*_JK_ = −0.11(2), *D*_K_ = 0.13(4) kHz.

f σ is the standard deviation of the fit. For species measured with multiple spectrometers, like #1 and #1′, all transition lines were included in a single global fit; the standard deviations of the individual subsets of lines are reported in the Supporting Information.

### Structural Symmetry and Mass Distribution

Conformers #1 and #1′ possess *C*_*s*_ symmetry, with the CC­(=O)­ON frame residing in the symmetry plane that corresponds to the *ac* principal inertial plane (σ_*ac*_). This was confirmed by the absence of μ_*b*_-type transitions and, for conformer #1, by the detection of the rotational transition belonging to the ^13^C isotopologues for the ethyl chains with intensity (ca. 2%) twice that expected for the natural abundance of the ^13^C isotope (ca. 1%). In contrast, conformers #2 and #3 lack this symmetry plane (*C*_1_ point group). These species differ from the global minimum #1 by the orientation of one lateral ethyl chain, which adopts a *gauche* or *gauche′* configuration rather than the extended *anti* arrangement. This structural desymmetrization is directly evidenced in the experimental spectrum of #2 by the activation of μ_*b*_-type transitions, which are strictly forbidden by symmetry in #1 and #1′. For #3, however, the expected electric dipole moment component along the *b* inertial axis is accidentally small (μ_*b*_ ≃0.1 D), precluding the observation of the corresponding lines.

The planar moments of inertia, which describe the mass (*m*) extension out of the molecular plane perpendicular to a given inertial axis (*g*):

3 $$P_{\mathrm{gg}}=\sum_{i=1}^{N_{atoms}}m_{i}\cdot g_{i}^{2},g=a,b,c$$

provide a sensitive probe of the ethyl chain orientation. In particular, considering the planar moment related to the axis perpendicular to the symmetry plane (out-of-plane component, *P*_oop_, hereafter) reveals a distinct structural deformation of the monomer backbone due to the presence and orientation of the acetyl group (Table 2). Compared to the DEHA analogue, for which *a* is the axis perpendicular to the molecular symmetry plane, the change is +1.33 u·Å^2^ for #1 and +3.60 u·Å^2^ for #1′. Given the acetic acid fragment contribution of +1.49 u·Å^2^,^42,43^ these values imply a contraction of the ethyl framework in #1 to maximize carbonyl interactions and a significant elongation in #1′ to alleviate steric repulsion with the acetyl methyl group. It is worth noting that this trend is confirmed at the MP2/aug-cc-pVTZ level of calculation, for which the rigid out-of-plane contribution of the acetyl group is 1.56 u·Å^2^ for #1 and 1.54 u·Å^2^ for #1′.

**Table 2:** Experimental and Calculated (MP2/aug-cc-pVTZ) Out-of-Plane (oop) Components of the Planar Moments of Inertia (*P*_oop_ in u·Å^2^) and ^14^N Nuclear Quadrupole Coupling Constants (χ_oop_ in MHz) for the Symmetric Observed Species of DEHA, DEAcA, and Their Water Complexes

	*P*_oop_^exp.^	*P*_oop_^calc.^	χ_oop_^exp.^	χ_oop_^calc.^
DEAcA#1′	238.9942(1)	236.143	0.524(9)	0.445
DEAcA#1	236.72415(6)	234.371	0.567(2)	0.490
DEAcA#1:1	237.2268(2)	234.998	0.442(5)	0.361
DEAcA#1:2	237.4973(3)	237.845	0.300(6)	0.188
DEHA^40^	235.39148(2)	233.265	0.711(2)	0.606
DEHA:W^15^	236.44297(9)	235.606	0.664(2)	0.513

The orientation of the acetyl group in CH_3_COOR compounds is typically dictated by the organyl group R. In acetic acid,^43^ peroxyacetic acid,^44^ and a wide array of esters (i.e., ethyl acetate,^45^ vinyl acetate,^46^ phenyl acetate^47^), the *syn* O=C–O–R arrangement is ubiquitous. This conformation is stabilized by the minimization of the electric dipole moment while simultaneously reducing steric interference with the acetyl methyl group.

In DEAcA, our experimental results confirm a consistent preference for this *syn* topology; the global minimum (conformer #1) adopts *C*_*s*_ symmetry with the carbonyl oxygen oriented toward the nitrogen lone pair. At first glance, this arrangement appears sterically and electronically challenging, as one might expect a more favorable interaction if the acetyl methyl group were oriented toward the basic nitrogen. However, while this geometry is consistent with the minimization of the molecular electric dipole moment, a well-established feature of many esters, it is expected to be stabilized by local stereoelectronic effects that reinforce this preference. The *syn* arrangement avoids the close proximity of the oxygen lone pairs that would be present in the alternative anticonfiguration. Consequently, both global electrostatic considerations and local stereoelectronic effects may contribute to the stabilization of the observed conformer.

### ^14^N Nuclear Quadrupole Coupling

The ^14^N NQC constants provide a sensitive probe of the EFG at the nitrogen nucleus. To properly interpret these values, it is necessary to distinguish the principal axes of inertia (*a*, *b*, *c*) from the principal axes of the EFG tensor (*x*, *y*, *z*) at the nitrogen atom, as depicted in Figure 3. For the *C*_*s*_ symmetric conformers #1 and #1′, symmetry dictates that the out-of-plane EFG axis (*y*) strictly coincides with the *b* inertial axis. Consequently, the experimentally determined χ_*bb*_ constant directly corresponds to the out-of-plane EFG component (χ_*yy*_ ≡ χ_oop_). Upon acetylation of DEHA, this out-of-plane component undergoes a significant 0.144 MHz reduction, moving from χ_*aa*_ in DEHA to χ_*bb*_ in DEAcA#1 (Table 2). This attenuation is well reproduced by MP2/aug-cc-pVTZ calculations (0.118 MHz) and indicates a substantial redistribution of the nitrogen lone-pair density. This reduction, coupled with the calculated ∼0.6 MHz shift in the in-plane components (χ_*yy*_ and χ_*zz*_), highlights the strong inductive electron-withdrawing effect of the *O*-acetyl group.

**Figure 3:**
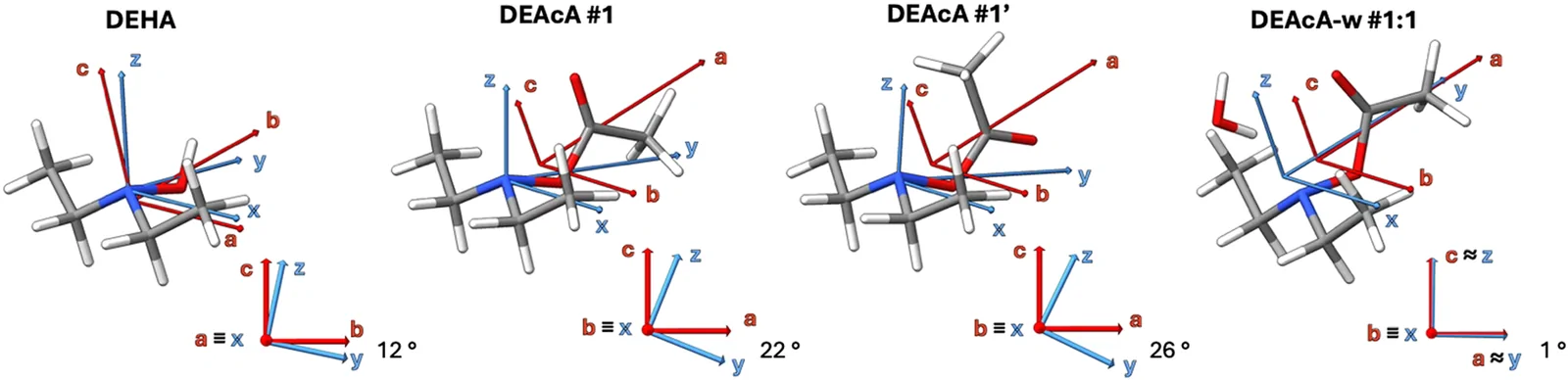
Comparison of the principal inertial axes (*a*, *b*, *c*; red) and the electric field gradient (EFG) principal axes (*x*, *y*, *z*; blue) at the nitrogen nucleus for the *C*_*s*_ conformers of DEHA, DEAcA, and the #1:1 DEAcA–water complex at the MP2/aug-cc-pVTZ level. For all three species, molecular symmetry dictates that the out-of-plane inertial axis (e.g., *a* for DEHA, *b* for DEAcA) strictly coincides with its *y* EFG principal axis. The resulting tilt angle between the remaining in-plane coordinate axes is indicated beneath each structure.

A similar trend is observed for the DEAcA#1′. However, the magnitude of the acetylation-induced shift is more pronounced in this conformer (exp. 0.187 MHz, calc. 0.161 MHz). This enhanced perturbation suggests that through-space interactions between the nitrogen electron cloud and the neighboring acetyl methyl or carbonyl groups further modulate the primary inductive effect. Such sensitivity of the NQC constants to the global conformation underscores the intricate balance of electronic forces governing the DEAcA scaffold.

For the *C*_1_ conformers #2 and #3, the lack of a molecular symmetry plane means the EFG principal axes are not constrained to align with the inertial framework. As a result, the nonzero off-diagonal elements of the coupling tensor prevent a direct geometric extraction of an isolated out-of-plane χ_*yy*_ component for simple comparison. Nevertheless, the experimentally measured diagonal components (χ_*aa*_, χ_*bb*_, χ_*cc*_) for these asymmetric species remain in good agreement with the theoretical predictions, rigorously confirming their respective structural assignments.

### Microsolvation

The hydration of conformer #1 exhibits highly site-selective microsolvation (Figure 4). In the #1:1 complex, the primary water molecule (hereafter labeled W1) anchors exclusively to the sp^3^ nitrogen lone pair, acting as a proton donor to the amine. This preference for the amine site over the sp^2^ carbonyl oxygen, despite the latter’s significant dipole moment, is driven by the superior basicity of the oxyamine nitrogen. The computed binding energy (BE = *E*_complex_ – Σ­(*E*_monomers_)) reveals a definitive thermodynamic hierarchy: stabilization is maximized at −42.6 kJ mol^–1^ for the nitrogen docking site, significantly outpacing the alternative orientations at the carbonyl oxygen (−30.0 kJ mol^–1^) and the ester oxygen (−22.8 kJ mol^–1^).

**Figure 4:**
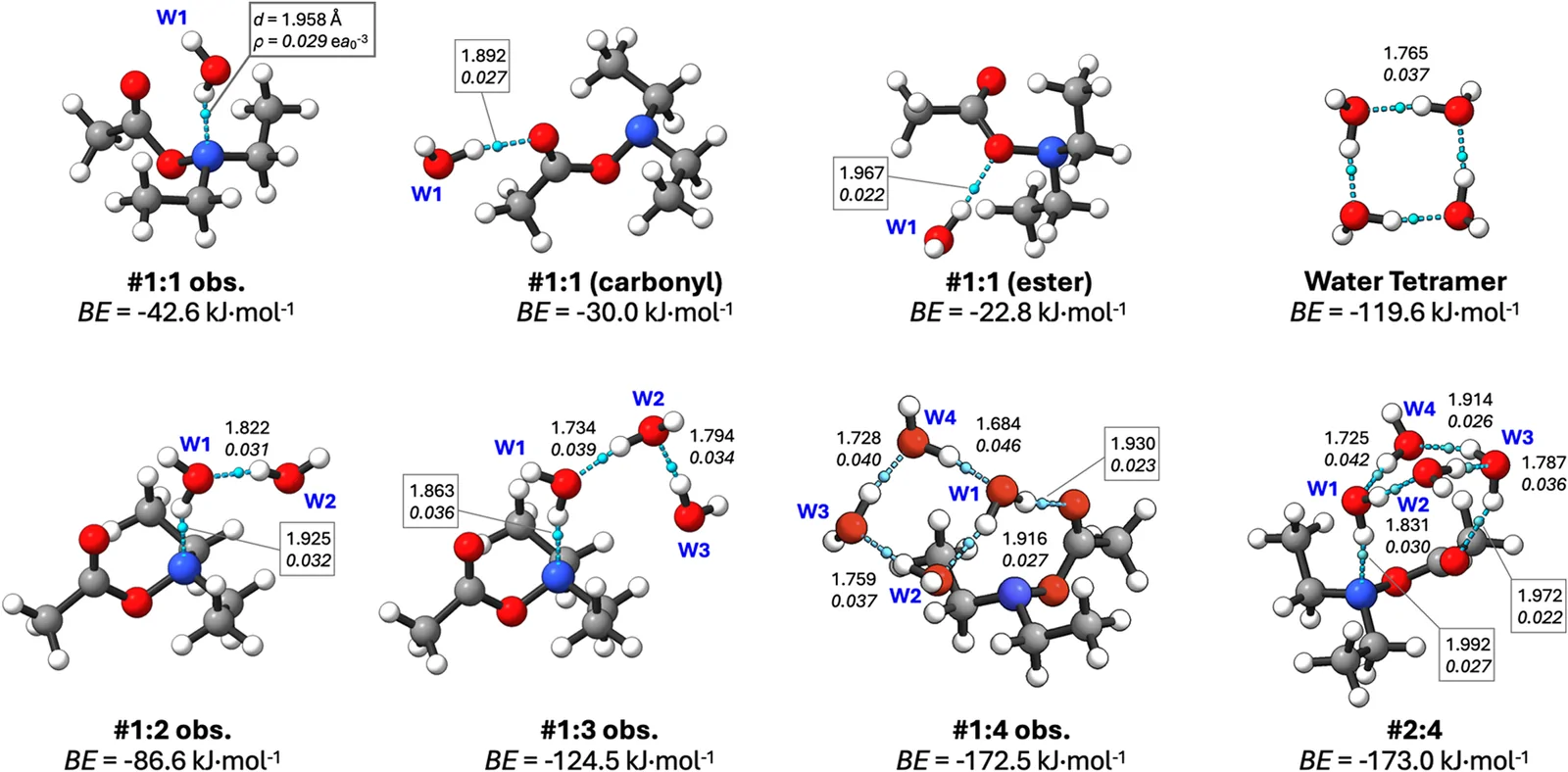
Geometries, hydrogen-bond distances (*d* in Å), electron densities (ρ in e·a_0_^–3^) at the hydrogen-bond critical points (small cyan spheres), and binding energy values (BE in kJ mol^–1^) of *N*,*N*-diethylacetyloxyamine–water clusters at the MP2/aug-cc-pVTZ level of calculation.

As the cluster expands to the #1:2 and #1:3 species, a sequential hydrogen-bonded water chain is established (N···H–O···H–O···H–O). In this growing network, W1 acts as a proton donor to a second water molecule (W2), which in turn acts as a proton donor to a third (W3). The experimental planar moments along the direction perpendicular to the symmetry plane of the monomer (Table 2) demonstrate that the water chain remains confined within the molecular symmetry plane up to *n* = 2, the increase with respect to the monomer value being attributable to large-amplitude motions of the water moieties, as observed for DEHA–water.^15^ Weak interactions between the water oxygen atoms and the ethyl-group pocket likely stabilize this structural motif (Figure S5). For the trihydrated form, the larger variation (*P*_*cc*_(#1:3) = 239.457(5) u·Å^2^) could be attributable to an out-of-plane arrangement of the water molecules (as suggested by theoretical computations), as well as an elongation of the ethyl chains to better accommodate the ligands.

The electronic impact of hydration is manifest in the systematic decrease of the χ_oop_^14^N NQC constant (Table 2). While the primary water molecule (W1) directly perturbs the nitrogen electric field gradient via hydrogen bonding, the continued attenuation in the #1:2 species highlights a significant cooperative effect: W2 polarizes the chain, strengthening the primary N···H interaction. This electronic perturbation is notably more pronounced than in DEHA, where complexation with a single water molecule yields a significantly smaller shift in χ_oop_.

To contextualize this electronic perturbation, we compared the experimental out-of-plane ^14^N NQC constants (χ_*bb*_) with the calculated values for a series of reference amines, including ammonia,^48^ diethylamine,^49^ hydroxylamine,^50^ DEHA,^40^ DEAcA, and its water complexes (*n* = 1–2), all of which exhibit a plane of symmetry with the nitrogen atom lying in the symmetry plane (Figure 5).

**Figure 5:**
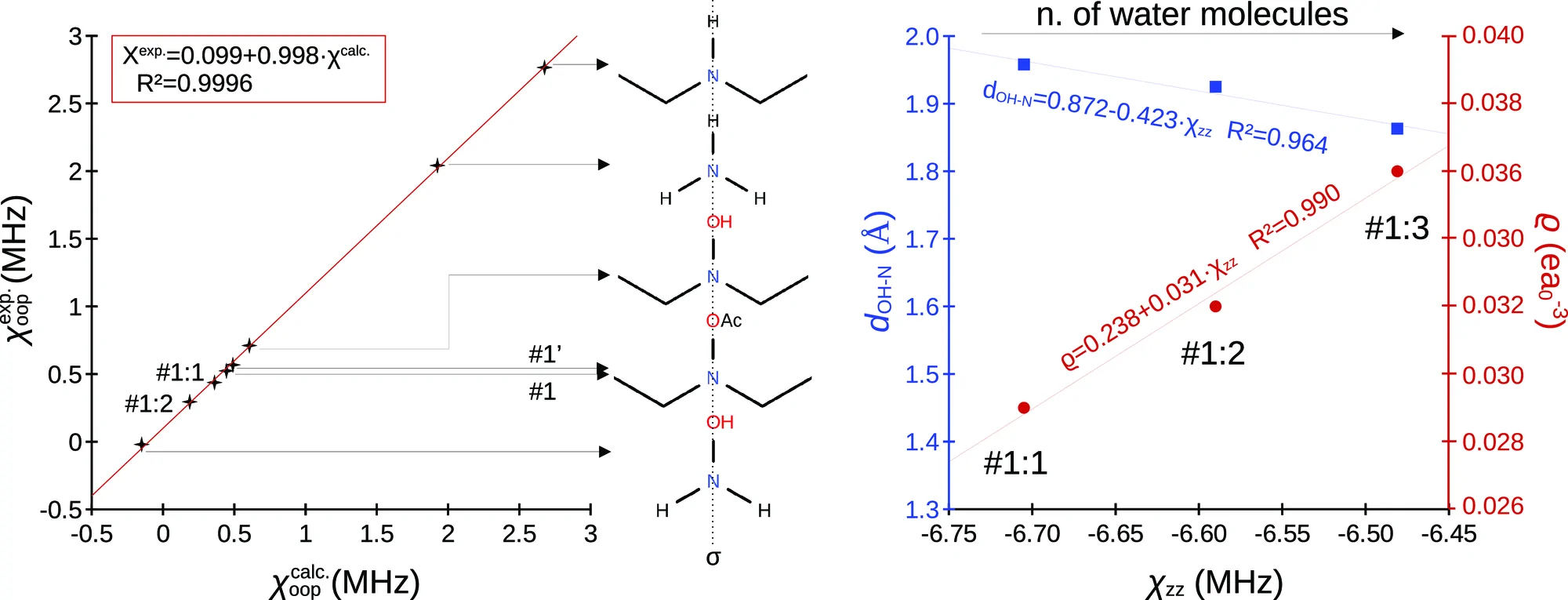
(Left) Comparison of the experimental and calculated (MP2/aug-cc-pVTZ) out-of-plane ^14^N nuclear quadrupole coupling constants for a series of related symmetric amines (σ plane of symmetry), including conformers #1 and #1′ of DEAcA and its mono- (1:1) and dihydrated (1:2) complexes. A systematic computational underestimation of approximately 0.1 MHz is observed across the series. (Right) Linear correlation of the computed primary N···H hydrogen bond length (*d*_OH–N_) and the QTAIM electron density at the bond critical point (ρ_BCP_) as a function of the principal ^14^N NQC tensor component χ_*zz*_, illustrating the cooperative polarization of the water network.

While the MP2/aug-cc-pVTZ level of theory systematically underestimates the absolute experimental quadrupole coupling constants by approximately 0.1 MHz, the relative structural trends are faithfully reproduced across the series. This theoretical fidelity allows us to confidently correlate the experimental NQC shifts with computationally derived intermolecular metrics. Specifically, the cooperative strengthening of the hydrogen-bond network is evidenced by a monotonic contraction of the primary N···H intermolecular distance (*d*_OH–N_), which shortens from 1.958 Å in the #1:1 complex to 1.925 and 1.863 Å in the #1:2 and #1:3 clusters, respectively. Concurrently, QTAIM analysis reveals a corresponding increase in electron density at the hydrogen-bond critical point (ρ), from 0.029 to 0.032 and ultimately to 0.036 e·a_0_^–3^ across the hydration series. As illustrated in Figure 5, these localized structural and topological markers scale linearly with the principal χ_*zz*_ tensor component. Because the *z*-axis aligns nearly parallel to the nitrogen lone pair involved in the hydrogen bond, the variation in χ_*zz*_ serves as a sensitive, direct spectroscopic proxy for lone-pair polarization within the nascent solvent network.

At higher hydration levels, the *n* = 4 cluster marks a fundamental transition in the solvation regime. Although the *n* = 1–3 series is governed by nitrogen lone-pair binding, the experimentally observed complex for conformer #1 (labeled #1:4) involves a cyclic water tetramer docking at the carbonyl oxygen via an O–H···O=C interaction. In this structural motif, a cyclic water tetramer is formed. The fourth water molecule (W4) accepts a hydrogen bond from W3 and donates one to W1, thereby closing the cycle. As a result, W1 simultaneously donates hydrogen bonds to DEAcA and W2, while accepting one from W4.

The detachment of the solvent network from the amine nitrogen in the #1:4 complex is unambiguously corroborated by the theoretical χ_*zz*_ nuclear quadrupole tensor component, which at 6.987 MHz is nearly identical to that of the isolated monomer #1 (6.985 MHz), confirming the absence of any primary N···H–O interaction. Once the water network has achieved substantial stabilization through four internal hydrogen bonds, its considerable steric bulk renders docking at the highly hindered sp^3^ nitrogen unfavorable. The anchoring of the water tetramer to DEAcA drives a significant redistribution of electron density within the solvent network itself. In an isolated cyclic water tetramer, the computed electron density (ρ) at the hydrogen bond critical points is 0.037 e·a_0_^–3^. Upon complexation to DEAcA, where W1 constitutes the anchor point, QTAIM calculations reveal a localized topological shift: the W1–W4 hydrogen bond is strengthened, while the W1–W2 bond is weakened relative to the isolated tetramer. Conversely, the W2–W3 interaction remains almost entirely unchanged.

Interestingly, theoretical computations reveal that the global minimum for the DEAcA–(H_2_O)_4_ system actually involves a less stable monomeric conformer (conformer #2), which is favored by a narrow margin over the #1:4 complex (0.47 kJ mol^–1^ MP2/aug-cc-pVTZ, 0.12 kJ mol^–1^ B3LYP-D3­(BJ)/def2-TZVP). This underlying conformational destabilization is effectively counterbalanced by stronger intermolecular binding: in the #2:4 structure, the water tetramer bridges the DEAcA molecule by forming two distinct anchoring bonds, with W1 coordinating to the aminic nitrogen and W3 binding to the carbonyl oxygen. However, vibrational frequency calculations within the harmonic approximation indicate that zero-point energy (ZPE) corrections decisively invert this stability order. Upon inclusion of the ZPE (B3LYP-D3­(BJ)/def2-TZVP), the #2:4 complex becomes 3.38 kJ mol^–1^ higher in energy than the #1:4 cluster. This ZPE-driven inversion aligns perfectly with the exclusive experimental observation of the #1:4 structural motif. However, the absence of the #2:4 complex may also have an alternative origin, as complex formation in the jet is governed by kinetic rather than thermodynamic control. Notably, conformer #2 is already absent in the mono-, di-, and trihydrated species. Even if the tetramer complex were formed directly from the monomer and a pre-existing water tetramer, the lower population of monomer #2 in the jet would naturally favor the observation of the #1:4 complex. Together, these findings confirm that the lone-pair-directed solvation regime yields to collective solvent–solvent and multidentate interactions at *n* = 4. Similar transitions have been computationally and experimentally noted for other proton-acceptor systems, such as sevoflurane (CH­(CF_3_)_2_OCH_2_F^51^), benzaldehyde (PhCHO^52^), and fenchone (C_10_H_16_O^53^).

## Conclusions

We provide a spectroscopic and computational account of the conformational and microsolvation landscape of *N,N*-diethylacetyloxyamine across the full hydration series (H_2_O)_*n*_, *n* = 0–4. The isolated monomer adopts a *syn* configuration, in which the acetyl group orients its carbonyl oxygen toward the nitrogen lone pair to minimize destabilizing oxygen–oxygen repulsions. The early stages of hydration (*n* = 1–3) exhibit strict site selectivity: despite the presence of a highly polar carbonyl group, water molecules bind exclusively to the sp^3^ nitrogen, initiating a sequential, *C*_*s*_-symmetric cooperative hydrogen-bonded chain. The observed trend in the ^14^N NQC constants provides direct experimental evidence of electronic cooperativity, demonstrating that each additional water molecule polarizes the network and strengthens the primary N···H bond. At *n* = 4, a fundamental transition in the solvation regime is experimentally confirmed: the global minimum shifts to a cyclic water tetramer anchored at the carbonyl oxygen, where collective solvent–solvent interactions override the site selectivity imposed by local basicity. This work thus establishes a complete, experimentally benchmarked picture of the competing forces that shape early solvation in multifunctional amines: localized stereoelectronic effects and lone-pair basicity dictate the architecture of the nascent solvation shell, whereas the cooperative stabilization of an extended hydrogen-bond network drives a topology change at higher hydration levels.

## Supplementary Material



## Footnotes

a Gaussian is a registered trademark of Gaussian, Inc., 340 Quinnipiac St. Bldg., 40 Wallingford, Connecticut 06492, United States.

## Notes

Experimental spectrometer configurations and measurement conditions; ab initio (MP2/aug-cc-pVTZ) equilibrium geometries and principal axis system structures for DEAcA conformers and their water complexes (#1:1 to #1:4 and #2:4); experimental and theoretical spectroscopic parameters; theoretical ^14^N nuclear quadrupole tensor components in the EFG principal axis system; Kraitchman substitution coordinates for the ethyl carbon atoms; potential energy surface scans; electron density topological (AIM/NCI) analysis of all water complexes; conformational search results (B3LYP-D3­(BJ)/def2-TZVP) for mono-, di-, tri-, and tetrahydrated DEAcA species; complete lists of assigned rotational transition frequencies with residuals for all measured species (PDF)
