# Supplementary material for: Switching Site Selectivity in Alkoxyamine Hydration: From Lone-Pair Direction to Solvent Network Dominance
Source: J Am Chem Soc. 2026 Jul 7;148(36):38731–9. doi: 10.1021/jacs.6c08250 (PMC13587680; doi:10.1021/jacs.6c08250)
Supplement: Supplementary file 1 [file ja6c08250_si_001.pdf]

# Supplementary Information

## Switching Site-Selectivity in Alkoxyamine Hydration: From Lone-Pair Direction to Solvent-Network Dominance

Filippo Baroncelli, Luca Evangelisti, Juan Carlos López,

Susana Blanco, Assimo Maris, Sonia Melandri

June 26, 2026

## Contents

**1 Experimental conditions** **4**

**2 Ab initio equilibrium molecular structures (MP2/aug-cc-pVTZ)** **5**
- 2.1 Conformers of the monomer . . . 5
- 2.2 #1:1 water complex . . . 12
- 2.3 #1:2 water complex . . . 15
- 2.4 #1:3 water complex . . . 16
- 2.5 #1:4 water complex . . . 17
- 2.6 #2:4 water complex . . . 18

**3 Experimental and ab initio spectroscopic parameters of the conformers of DEAcA.** **19**

**4 Experimental and ab initio spectroscopic parameters for the #1:1 water complex** **24**

**5 Experimental and ab initio spectroscopic parameters of the #1:2 water complex** **25**

**6 Experimental and ab initio spectroscopic parameters of the #1:3 water complex** **26**

**7 Experimental and ab initio spectroscopic parameters of the #1:4 water complex** **27**

**8 Ab initio spectroscopic parameters for the #2:4 water complex** **28**

**9 Ab initio principal components of the $^{14}$N nuclear quadrupole tensor.** **29**

**10 Kraitchman's substitution coordinates** **30**

**11 Electron density topological analysis** **31**

**12 Conformational potential energy profiles for DEAcA.** **34**

**13 Conformers of DEAcA water complexes from DFT search** **35**

**14 Experimental rotational transition frequencies** **45**

## List of Tables

S1 Spectrometers setups and working conditions. . . . 4

S2 Principal axis system structure of #1-DEAcA (aaAs, MP2/aug-cc-pVTZ). The *a*, *b*, and *c* axes are color-coded following the RGB scheme: red, green, and blue. . . . 6

S3 Principal axis system structure of #1'-DEAcA (aaAa, MP2/aug-cc-pVTZ). The $a$, $b$, and $c$ axes are color-coded following the RGB scheme: red, green, and blue. . . . 7
S4 Principal axis system structure of #2-DEAcA (gaAs, MP2/aug-cc-pVTZ). The $a$, $b$, and $c$ axes are color-coded following the RGB scheme: red, green, and blue. . . . 8
S5 Principal axis system structure of #2'-DEAcA (gaAa, MP2/aug-cc-pVTZ). The $a$, $b$, and $c$ axes are color-coded following the RGB scheme: red, green, and blue. . . . 9
S6 Principal axis system structure of #3-DEAcA (agAs, MP2/aug-cc-pVTZ). The $a$, $b$, and $c$ axes are color-coded following the RGB scheme: red, green, and blue. . . . 10
S7 Principal axis system structure of #3'-DEAcA (agAa, MP2/aug-cc-pVTZ). The $a$, $b$, and $c$ axes are color-coded following the RGB scheme: red, green, and blue. . . . 11
S8 Principal axis system structure of the observed #1:1 water complex of #1-DEAcA (MP2/aug-cc-pVTZ). The $a$, $b$, and $c$ axes are color-coded following the RGB scheme: red, green, and blue. . . . 12
S9 Principal axis system structure of the #1:1 (carbonyl) water complex of #1-DEAcA (MP2/aug-cc-pVTZ). The $a$, $b$, and $c$ axes are color-coded following the RGB scheme: red, green, and blue. . . . 13
S10 Principal axis system structure of the #1:1 (ester) water complex of #1-DEAcA (MP2/aug-cc-pVTZ). The $a$, $b$, and $c$ axes are color-coded following the RGB scheme: red, green, and blue. . . . 14
S11 Principal axis system structure of the observed #1:2 water complex of #1-DEAcA (MP2/aug-cc-pVTZ). The $a$, $b$, and $c$ axes are color-coded following the RGB scheme: red, green, and blue. . . . 15
S12 Principal axis system structure of the observed #1:3 water complex of #1-DEAcA (MP2/aug-cc-pVTZ). The $a$, $b$, and $c$ axes are color-coded following the RGB scheme: red, green, and blue. . . . 16
S13 Principal axis system structure of the observed #1:4 water complex of #1-DEAcA (MP2/aug-cc-pVTZ). The $a$, $b$, and $c$ axes are color-coded following the RGB scheme: red, green, and blue. . . . 17
S14 Principal axis system structure of #2:4 water complex of #2-DEAcA (MP2/aug-cc-pVTZ). The $a$, $b$, and $c$ axes are color-coded following the RGB scheme: red, green, and blue. . . . 18
S15 Experimental (S-reduction, $III^{\ell}$-representation) and theoretical (MP2/aug-cc-pVTZ) spectroscopic parameters for #1-DEAcA (aaAs) and its $^{13}$C1 and $^{13}$C2 isotopologues. . . . 19
S16 Experimental (S-reduction, $III^{\ell}$-representation) and theoretical (MP2/aug-cc-pVTZ) parameters of #1'-DEAcA (aaAa). . . . 20
S17 Experimental (S-reduction, $I^{r}$-representation) and theoretical (MP2/aug-cc-pVTZ) parameters of #2-DEAcA (gaAs). . . . 21
S18 Experimental (S-reduction, $I^{r}$-representation) and theoretical (MP2/aug-cc-pVTZ) parameters of #3-DEAcA (agAs). . . . 22
S19 Theoretical (MP2/aug-cc-pVTZ) parameters of #2'-DEAcA (gaAa) and #3'-DEAcA (agAa). . . . 23
S20 Experimental (S-reduction, $I^{r}$-representation) and theoretical (MP2/aug-cc-pVTZ) parameters of the #1:1 water complex of #1-DEAcA. . . . 24
S21 Theoretical (MP2/aug-cc-pVTZ) parameters of the #1:1 (carbonyl) and #1:1 (ester) water complexes of #1-DEAcA. . . . 24
S22 Experimental (S-reduction, $I^{r}$-representation) and theoretical (MP2/aug-cc-pVTZ) parameters for the #1:2 water complex of #1-DEAcA. . . . 25
S23 Experimental (S-reduction, $I^{r}$-representation) and theoretical (MP2/aug-cc-pVTZ) parameters for the #1:3 water complex of #1-DEAcA. . . . 26
S24 Experimental (S-reduction, $I^{r}$-representation) and theoretical (MP2/aug-cc-pVTZ) parameters for the #1:4 water complex of #1-DEAcA. . . . 27
S25 Theoretical (MP2/aug-cc-pVTZ) parameters of the #2:4 water complex of #2-DEAcA. . . . 28
S26 Theoretical (MP2/aug-cc-pVTZ) $^{14}$N nuclear quadrupole coupling constants in the electric-field-gradient principal-axis system and the asymmetry parameter $\eta = (\chi_{xx} - \chi_{yy})/\chi_{zz}$ for the most stable conformers of DEAcA and its observed water complexes. . . . 29
S27 Principal axis system coordinates (in Å) of the ethyl carbon atoms of #1-DEAcA obtained with Kraitchman's substitution method ($r_s$) and ab initio calculations ($r_e$, MP2/aug-cc-pVTZ). . . . 30
S28 Theoretical (B3LYP-D3(BJ)/def2-TZVP) energy and spectroscopic parameters of the mono-hydrated species of DEAcA. . . . 35
S29 Theoretical (B3LYP-D3(BJ)/def2-TZVP) energy and spectroscopic parameters of the di-hydrated species of DEAcA. . . . 36
S30 Theoretical (B3LYP-D3(BJ)/def2-TZVP) energy and spectroscopic parameters of the tri-hydrated species of DEAcA. . . . 37
S31 Theoretical (B3LYP-D3(BJ)/def2-TZVP) energy and spectroscopic parameters of the tetra-hydrated species of DEAcA. . . . 39
S32 Experimental rotational transition frequencies (MHz) for #1-DEAcA (aaAs). . . . 45
S33 Experimental rotational transition frequencies (MHz) for #1-DEAcA($^{13}$C1, aaAs). . . . 53
S34 Experimental rotational transition frequencies (MHz) for #1-DEAcA($^{13}$C2, aaAs). . . . 54

S35 Experimental rotational transition frequencies (MHz) for #1'-DEAcA (aaAa). . . . . 55
S36 Experimental rotational transition frequencies (MHz) for #2-DEAcA (gaAs). . . . . 56
S37 Experimental rotational transition frequencies (MHz) for #3-DEAcA (agAs). . . . . 59
S38 Experimental rotational transition frequencies (MHz) for the #1:1 water complex of #1-DEAcA (aaAs:1w). . . . . 60
S39 Experimental rotational transition frequencies (MHz) for the #1:2 water complex of #1-DEAcA (aaAs:2w) . . . . . 62
S40 Experimental rotational transition frequencies (MHz) for the #1:3 water complex of #1-DEAcA (aaAs:3w). . . . . 65
S41 Experimental rotational transition frequencies (MHz) for the #1:4 water complex of #1-DEAcA (aaAs:4w). . . . . 67

# List of Figures

S-I Numbering of the skeletal atoms of *N*,*N*-diethylacetyloxyamine. . . . . 5
S-II Electron density topological analysis of #1:1 water complex of #1-DEAcA at the MP2/aug-cc-pVTZ level of calculation. . . . . 31
S-III Electron density topological analysis of #1:1 (carbonyl) water complex of #1-DEAcA at the MP2/aug-cc-pVTZ level of calculation. . . . . 31
S-IV Electron density topological analysis of #1:1 (ester) water complex of #1-DEAcA at the MP2/aug-cc-pVTZ level of calculation. . . . . 32
S-V Electron density topological analysis of #1:2 water complex of #1-DEAcA at the MP2/aug-cc-pVTZ level of calculation. . . . . 32
S-VI Electron density topological analysis of #1:3 water complex of #1-DEAcA at the MP2/aug-cc-pVTZ level of calculation. . . . . 32
S-VII Electron density topological analysis of #1:4 water complex of #1-DEAcA at the MP2/aug-cc-pVTZ level of calculation. . . . . 33
S-VIII Electron density topological analysis of #2:4 water complex of #2-DEAcA at the MP2/aug-cc-pVTZ level of calculation. . . . . 33
S-IX Relaxed potential energy scan for the acetyl torsion (O1N1-O2C5, step 12°) in #1-DEAcA at the B3LYP-D3(BJ)/def2-TZVP level of calculation. . . . . 34
S-X Relaxed potential energy scan for the ethyl torsion (C1C2-N1C3, step 10°) in #1-DEAcA at the B3LYP-D3(BJ)/def2-TZVP level of calculation. . . . . 34

# 1 Experimental conditions

Table S1: Spectrometers setups and working conditions.

.

| | MB-CP-FTMW[a] | PJ-FTMW[b] | FJ-AMMW |
|---|---|---|---|
| Frequency range | 2-8 GHz | 6.5-18.5 GHz | 59.6-74.5 GHz |
| Accuracy | 10 kHz | 3 kHz | 50 kHz |
| Resolution | 50 kHz | 7 kHz | 300 kHz |
| Carrier gas | He, Ne | He | Ar |
| Gas flow | Pulsed | Pulsed | Continuous |
| Pinhole nozzle diameter | 0.8 | 0.5 mm | 0.3 mm |
| Stagnation pressure | 300 kPa | 400 kPa | 19 kPa |
| Background pressure | $\simeq 10^{-3}$ Pa | $\simeq 10^{-3}$ Pa | 0.2 Pa |
| Stagnation temperature | 298 K | 298 K | 298 K |
| Rotational temperature | 1 K | 1 K | 10 K |
| Molecular Beam and radiation alignment | Perpendicular | Parallel | Perpendicular |
| Location | Valladolid | Bologna | Bologna |
| Molecular Species Observed | All | #1, #1' | #1 |

[a] The direct-digital CP-FTMW instrument uses an arbitrary-waveform generator (25 MSamples/s) digital source, followed by amplification with a travelling-wave tube (250 W). A set of eight chirp pulses was used for each gas pulse.
[b] Expansion takes place through a solenoid valve (General Valve, Series 9) into the Fabry-Pérot cavity. The spectral line positions are determined after Fourier transforming the time-domain signal, which has 8000 data points and was recorded with 100 ns sample intervals. Each rotational transition appears as a doublet due to the Doppler effect; the line position is calculated as the arithmetic mean of the Doppler-shifted frequencies.

# 2 Ab initio equilibrium molecular structures (MP2/aug-cc-pVTZ)

## 2.1 Conformers of the monomer

The conformers of DEAcA are labelled with a four-letter code that describes the *syn*, *anti* or gauche arrangements of the ethyl chains (C1C2N1C3, C4C3N1C2), the acetyloxy group (C5O1N1X, where X is a point along the bisector line of the CNC bond angle) and the acetyl group (O2C5O1N1), respectively. Non-equivalent *syn* forms are denoted by a single or double prime.

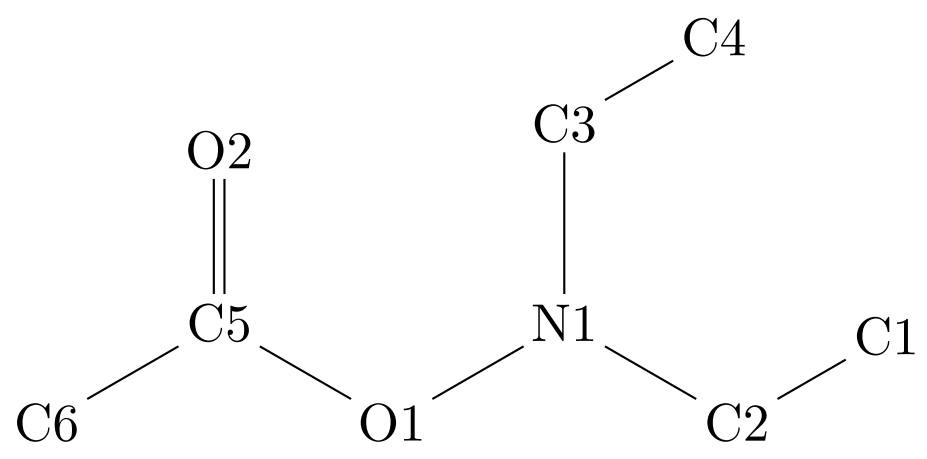


Figure S-I: Numbering of the skeletal atoms of *N*,*N*-diethylacetyloxyamine.

Table S2: Principal axis system structure of #1-DEAcA (aaAs, MP2/aug-cc-pVTZ). The $a$, $b$, and $c$ axes are color-coded following the RGB scheme: red, green, and blue.

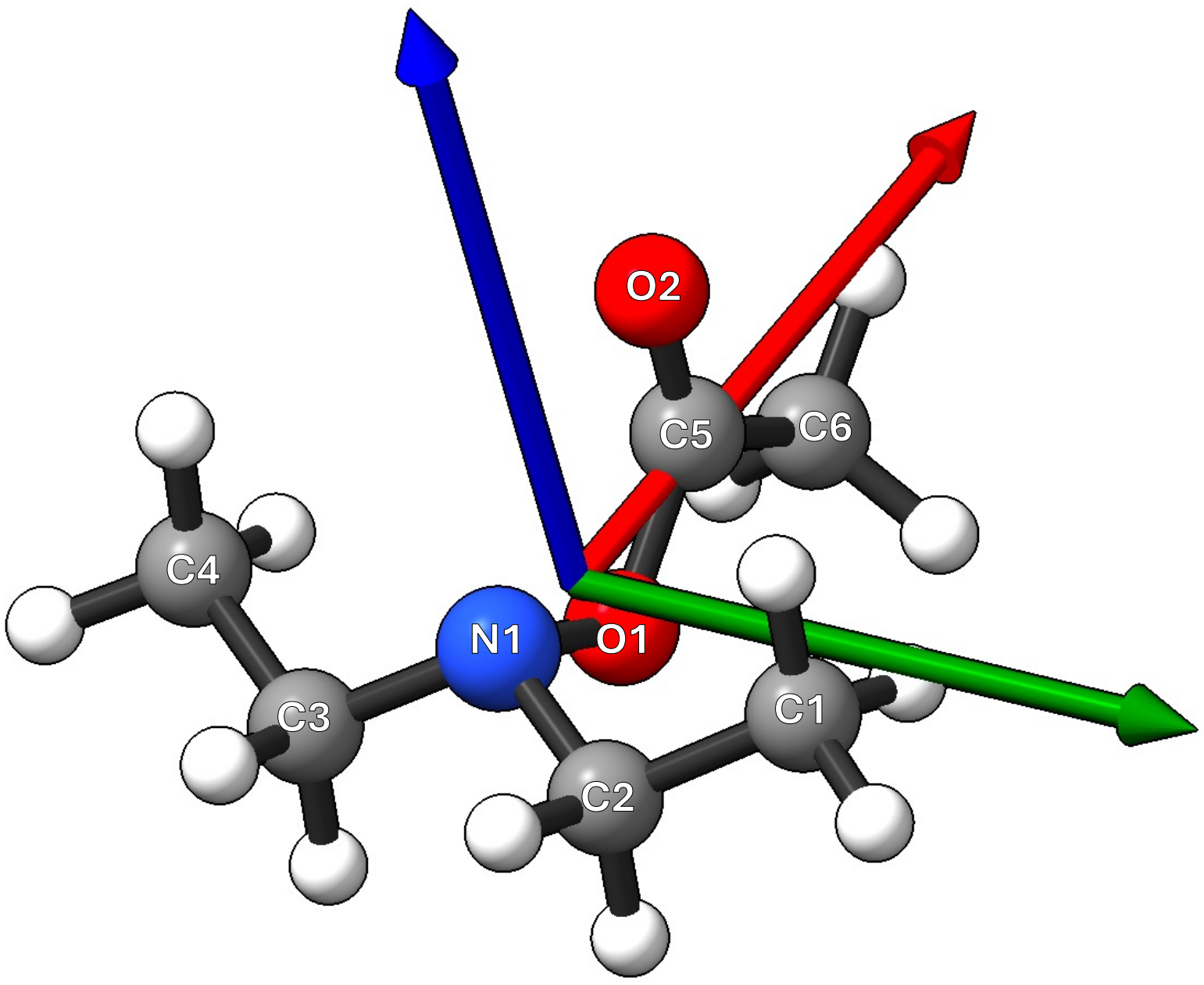


| atom | $a$/Å | $b$/Å | $c$/Å |
|---|---|---|---|
| C1 | −0.8917 | 2.4561 | −0.2114 |
| C2 | −1.6215 | 1.2079 | 0.2449 |
| N1 | −0.9097 | 0.0000 | −0.1639 |
| C3 | −1.6215 | −1.2079 | 0.2449 |
| C4 | −0.8917 | −2.4561 | −0.2114 |
| O1 | 0.2968 | 0.0000 | 0.6402 |
| C5 | 1.4704 | 0.0000 | −0.1080 |
| O2 | 1.4704 | 0.0000 | −1.3104 |
| C6 | 2.6168 | 0.0000 | 0.8351 |
| H1 | −1.4764 | 3.3430 | 0.0303 |
| H2 | −0.7251 | 2.4221 | −1.2864 |
| H3 | 0.0732 | 2.5439 | 0.2850 |
| H4 | −2.5991 | 1.1448 | −0.2359 |
| H5 | −1.7767 | 1.2156 | 1.3325 |
| H6 | −1.4764 | −3.3430 | 0.0303 |
| H7 | −0.7251 | −2.4221 | −1.2864 |
| H8 | 0.0732 | −2.5439 | 0.2850 |
| H9 | −2.5991 | −1.1448 | −0.2359 |
| H10 | −1.7767 | −1.2156 | 1.3325 |
| H11 | 2.5760 | 0.8792 | 1.4753 |
| H12 | 2.5760 | −0.8792 | 1.4753 |
| H13 | 3.5347 | 0.0000 | 0.2574 |

Table S3: Principal axis system structure of #1'-DEAcA (aaAa, MP2/aug-cc-pVTZ). The $a$, $b$, and $c$ axes are color-coded following the RGB scheme: red, green, and blue.

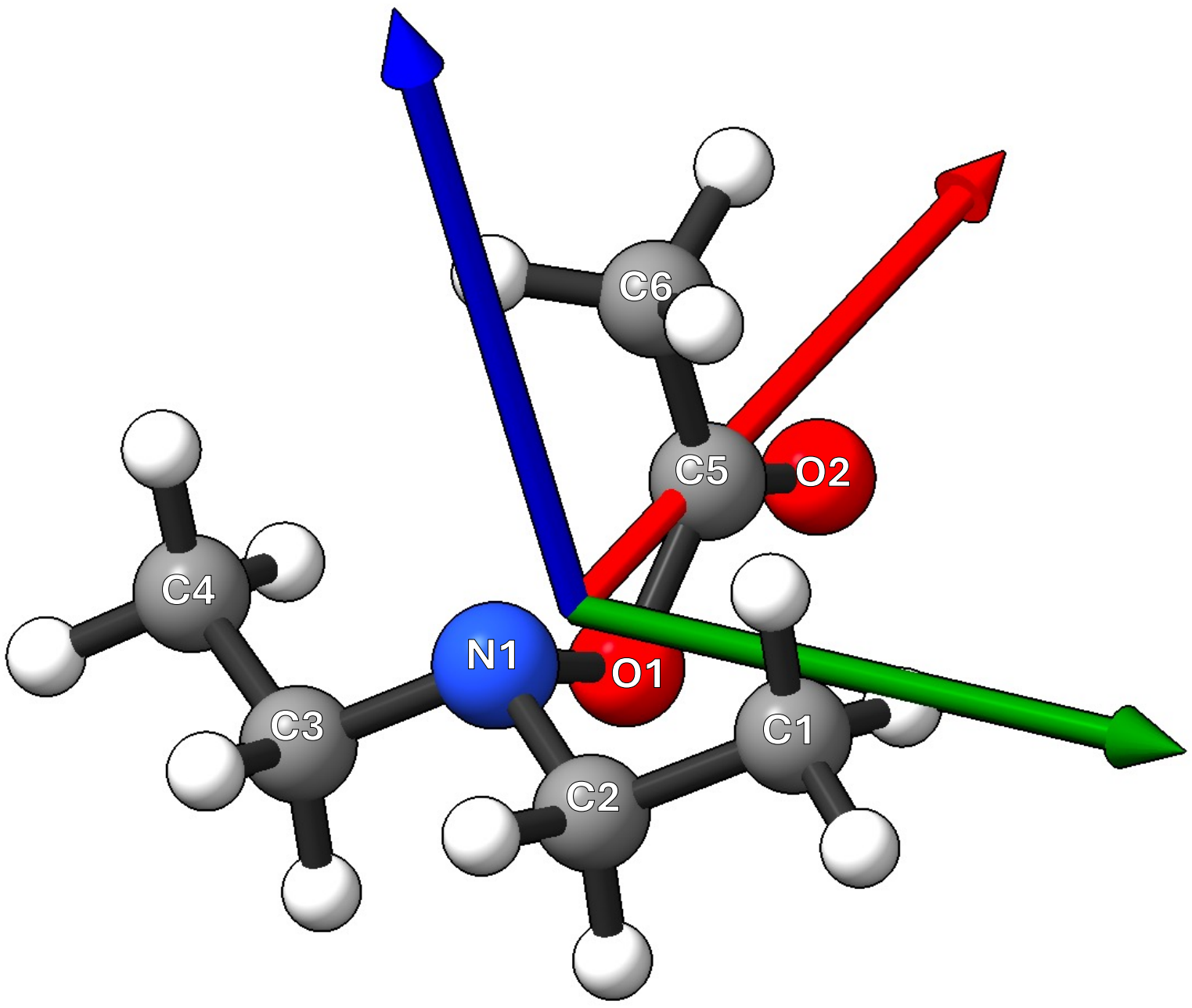


| atom | $a$/Å | $b$/Å | $c$/Å |
|---|---|---|---|
| C1 | −0.8849 | −2.4695 | −0.2082 |
| C2 | −1.6121 | −1.2024 | 0.1953 |
| N1 | −0.8639 | 0.0000 | −0.1812 |
| C3 | −1.6121 | 1.2024 | 0.1953 |
| C4 | −0.8849 | 2.4695 | −0.2082 |
| H1 | −1.4684 | −3.3420 | 0.0835 |
| H2 | −0.7372 | −2.5022 | −1.2867 |
| H3 | 0.0836 | −2.5346 | 0.2847 |
| H4 | −2.5679 | −1.1310 | −0.3263 |
| H5 | −1.8101 | −1.1944 | 1.2746 |
| H6 | −1.4684 | 3.3420 | 0.0835 |
| H7 | −0.7372 | 2.5022 | −1.2867 |
| H8 | 0.0836 | 2.5346 | 0.2847 |
| H9 | −2.5679 | 1.1310 | −0.3263 |
| H10 | −1.8101 | 1.1944 | 1.2746 |
| O1 | 0.2803 | 0.0000 | 0.7110 |
| C5 | 1.5019 | 0.0000 | 0.0859 |
| O2 | 2.4784 | 0.0000 | 0.7957 |
| C6 | 1.5287 | 0.0000 | −1.4141 |
| H11 | 1.0144 | 0.8739 | −1.8049 |
| H12 | 1.0144 | −0.8739 | −1.8049 |
| H13 | 2.5700 | 0.0000 | −1.7199 |

Table S4: Principal axis system structure of #2-DEAcA (gaAs, MP2/aug-cc-pVTZ). The $a$, $b$, and $c$ axes are color-coded following the RGB scheme: red, green, and blue.

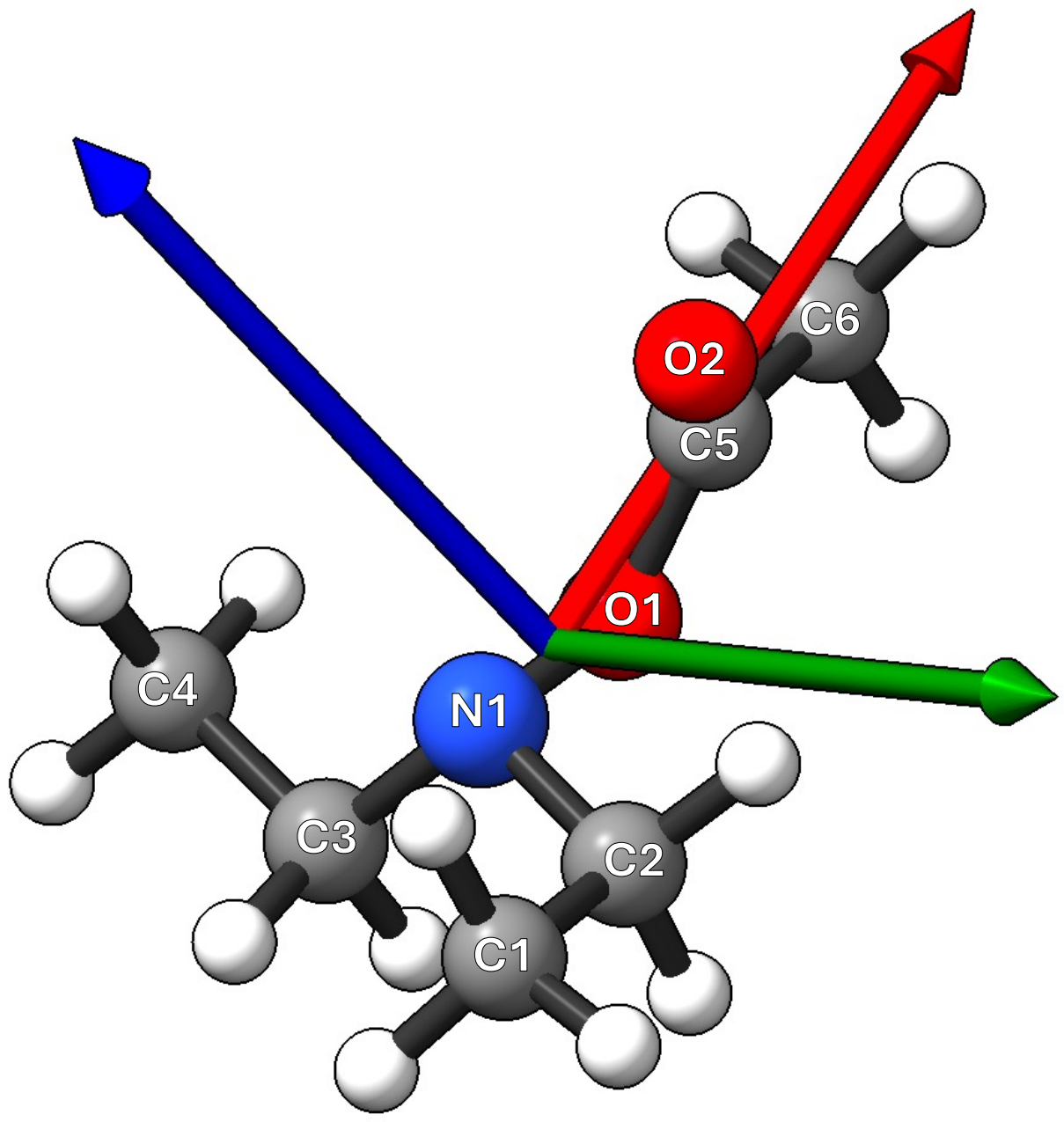


| atom | $a$/Å | $b$/Å | $c$/Å |
|---|---|---|---|
| C1 | −2.4305 | 1.6820 | −0.1223 |
| C2 | −1.1815 | 1.1663 | 0.5725 |
| N1 | −0.6783 | −0.0360 | −0.0982 |
| C3 | −1.4715 | −1.2181 | 0.2273 |
| C4 | −0.9749 | −2.4406 | −0.5201 |
| O1 | 0.6025 | −0.3259 | 0.5274 |
| C5 | 1.6438 | 0.2921 | −0.1042 |
| O2 | 1.5298 | 1.0944 | −0.9968 |
| C6 | 2.9328 | −0.1928 | 0.4977 |
| H1 | −2.2352 | 1.8152 | −1.1849 |
| H2 | −2.7101 | 2.6451 | 0.3030 |
| H3 | −3.2786 | 1.0107 | −0.0031 |
| H4 | −1.3664 | 0.9669 | 1.6365 |
| H5 | −0.3941 | 1.9133 | 0.4929 |
| H6 | −1.6183 | −3.2950 | −0.3126 |
| H7 | 0.0380 | −2.6942 | −0.2151 |
| H8 | −0.9783 | −2.2512 | −1.5921 |
| H9 | −1.4739 | −1.3904 | 1.3124 |
| H10 | −2.4906 | −0.9830 | −0.0783 |
| H11 | 2.8342 | −0.3268 | 1.5715 |
| H12 | 3.1817 | −1.1573 | 0.0562 |
| H13 | 3.7204 | 0.5173 | 0.2683 |

Table S5: Principal axis system structure of #2'-DEAcA (gaAa, MP2/aug-cc-pVTZ). The $a$, $b$, and $c$ axes are color-coded following the RGB scheme: red, green, and blue.

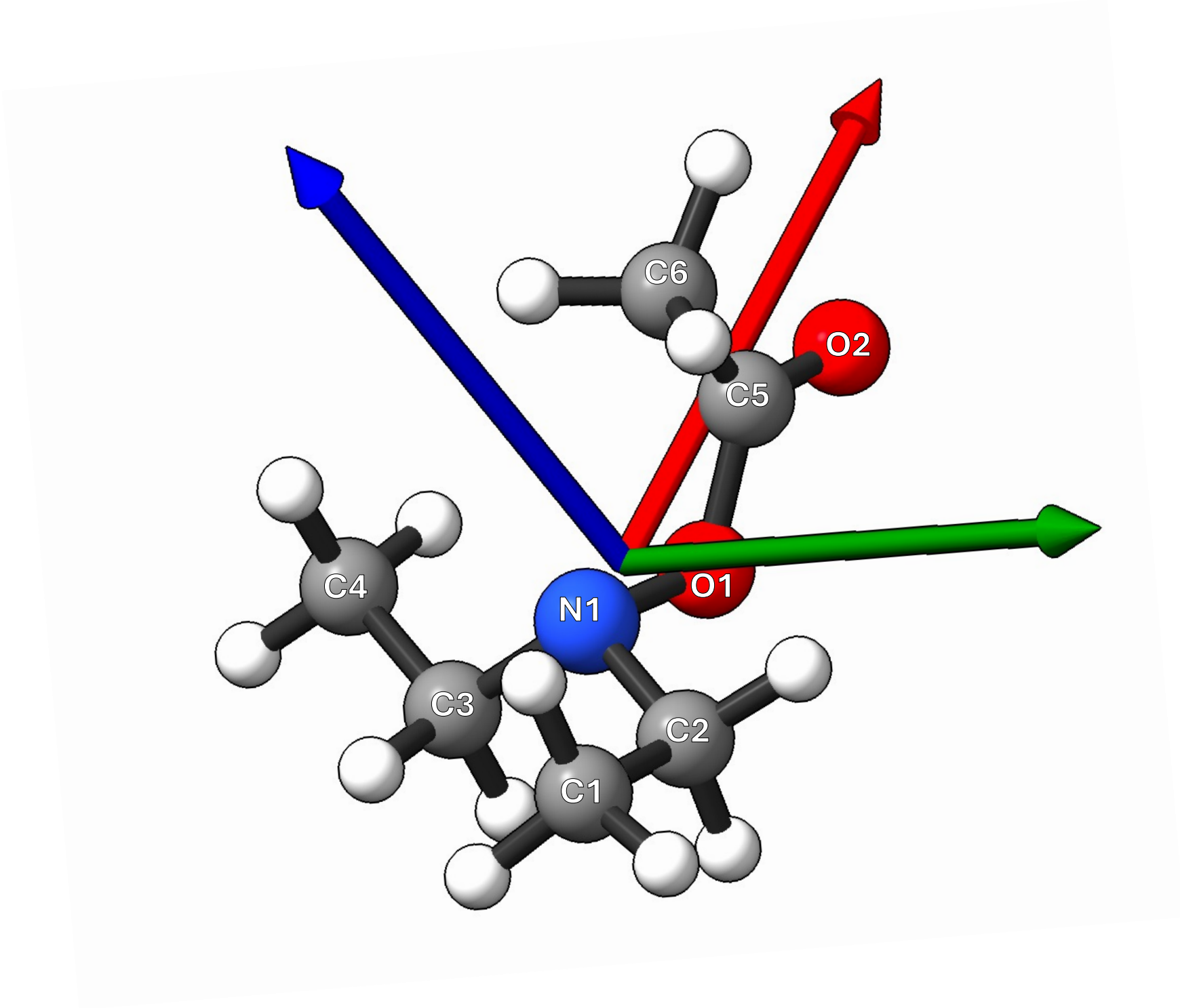


| atom | $a$/Å | $b$/Å | $c$/Å |
|---|---|---|---|
| C1 | −2.7538 | 1.2708 | −0.3046 |
| C2 | −1.4791 | 1.0835 | 0.5009 |
| N1 | −0.6605 | −0.0008 | −0.0496 |
| C3 | −1.2288 | −1.3165 | 0.2455 |
| C4 | −0.3918 | −2.4288 | −0.3555 |
| O1 | 0.5563 | 0.0355 | 0.7410 |
| C5 | 1.6834 | 0.3520 | 0.0256 |
| O2 | 2.7300 | 0.3675 | 0.6263 |
| C6 | 1.5235 | 0.6571 | −1.4352 |
| H1 | 0.6130 | −2.4264 | 0.0625 |
| H2 | −0.8449 | −3.3957 | −0.1399 |
| H3 | −0.3255 | −2.3148 | −1.4367 |
| H4 | −2.2266 | −1.3214 | −0.1923 |
| H5 | −1.3298 | −1.4433 | 1.3312 |
| H6 | −2.5222 | 1.3721 | −1.3640 |
| H7 | −3.4470 | 0.4418 | −0.1788 |
| H8 | −3.2608 | 2.1764 | 0.0258 |
| H9 | −0.8709 | 1.9862 | 0.4431 |
| H10 | −1.6989 | 0.8990 | 1.5601 |
| H11 | 2.5012 | 0.9291 | −1.8197 |
| H12 | 0.8135 | 1.4672 | −1.5850 |
| H13 | 1.1324 | −0.2096 | −1.9621 |

Table S6: Principal axis system structure of #3-DEAcA (agAs, MP2/aug-cc-pVTZ). The $a$, $b$, and $c$ axes are color-coded following the RGB scheme: red, green, and blue.

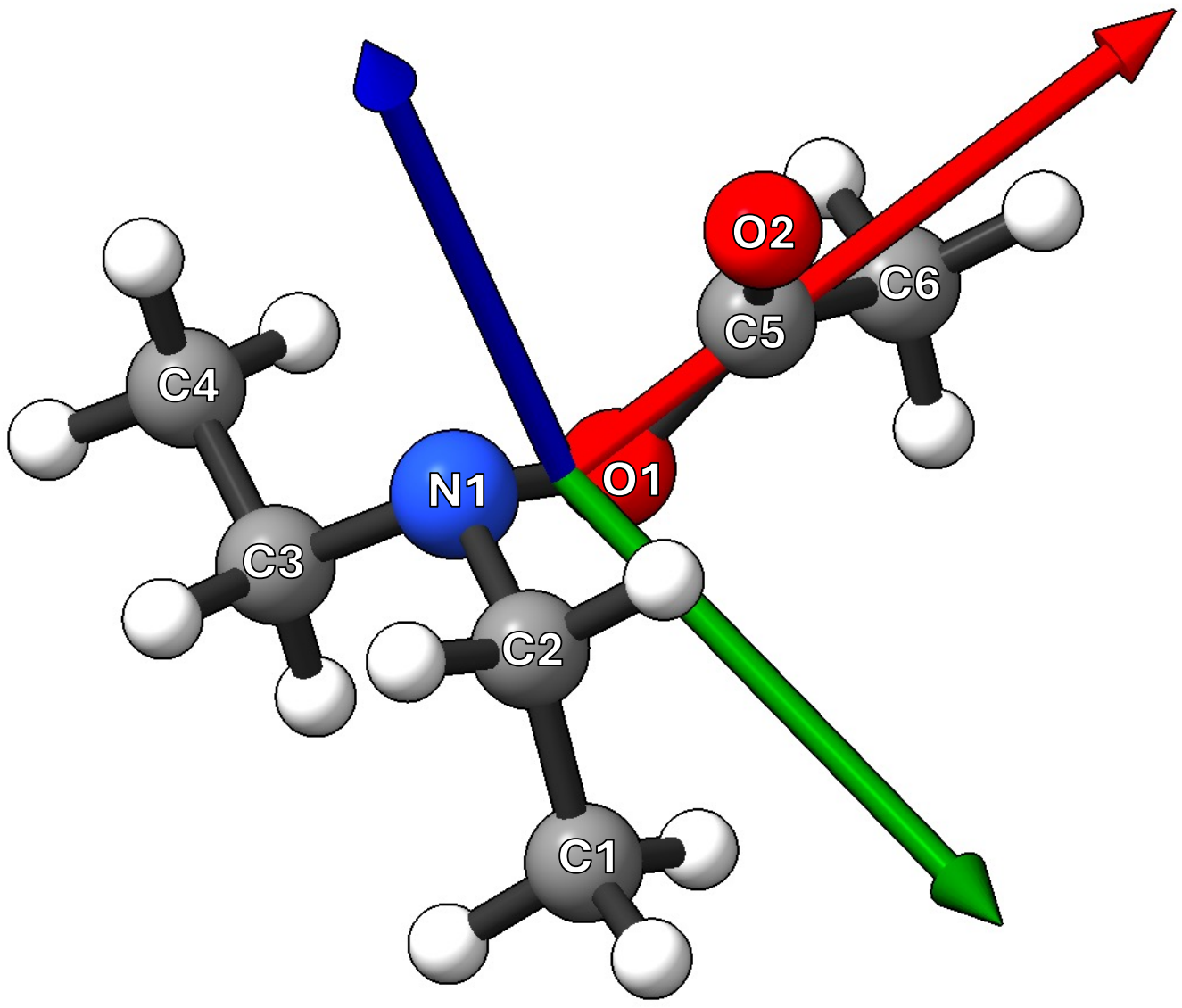


| atom | $a$/Å | $b$/Å | $c$/Å |
|---|---|---|---|
| C1 | −1.5286 | 2.0303 | 0.7430 |
| C2 | −1.0802 | 1.3709 | −0.5553 |
| N1 | −0.7078 | −0.0458 | −0.4755 |
| C3 | −1.7540 | −0.8878 | 0.0966 |
| C4 | −1.3947 | −2.3555 | −0.0342 |
| O1 | 0.3730 | −0.1462 | 0.4909 |
| C5 | 1.6037 | 0.0050 | −0.0813 |
| O2 | 1.7975 | 0.3252 | −1.2271 |
| C6 | 2.6569 | −0.2917 | 0.9495 |
| H1 | −0.4918 | −2.5776 | 0.5315 |
| H2 | −2.2004 | −2.9791 | 0.3522 |
| H3 | −1.2210 | −2.6090 | −1.0783 |
| H4 | −2.6537 | −0.6614 | −0.4792 |
| H5 | −1.9492 | −0.6309 | 1.1439 |
| H6 | −1.6852 | 3.0952 | 0.5728 |
| H7 | −2.4633 | 1.6122 | 1.1131 |
| H8 | −0.7703 | 1.9190 | 1.5163 |
| H9 | −1.8730 | 1.4108 | −1.3050 |
| H10 | −0.2177 | 1.8873 | −0.9747 |
| H11 | 2.3552 | 0.0666 | 1.9300 |
| H12 | 3.5921 | 0.1652 | 0.6428 |
| H13 | 2.7903 | −1.3714 | 1.0082 |

Table S7: Principal axis system structure of #3'-DEAcA (agAa, MP2/aug-cc-pVTZ). The $a$, $b$, and $c$ axes are color-coded following the RGB scheme: red, green, and blue.

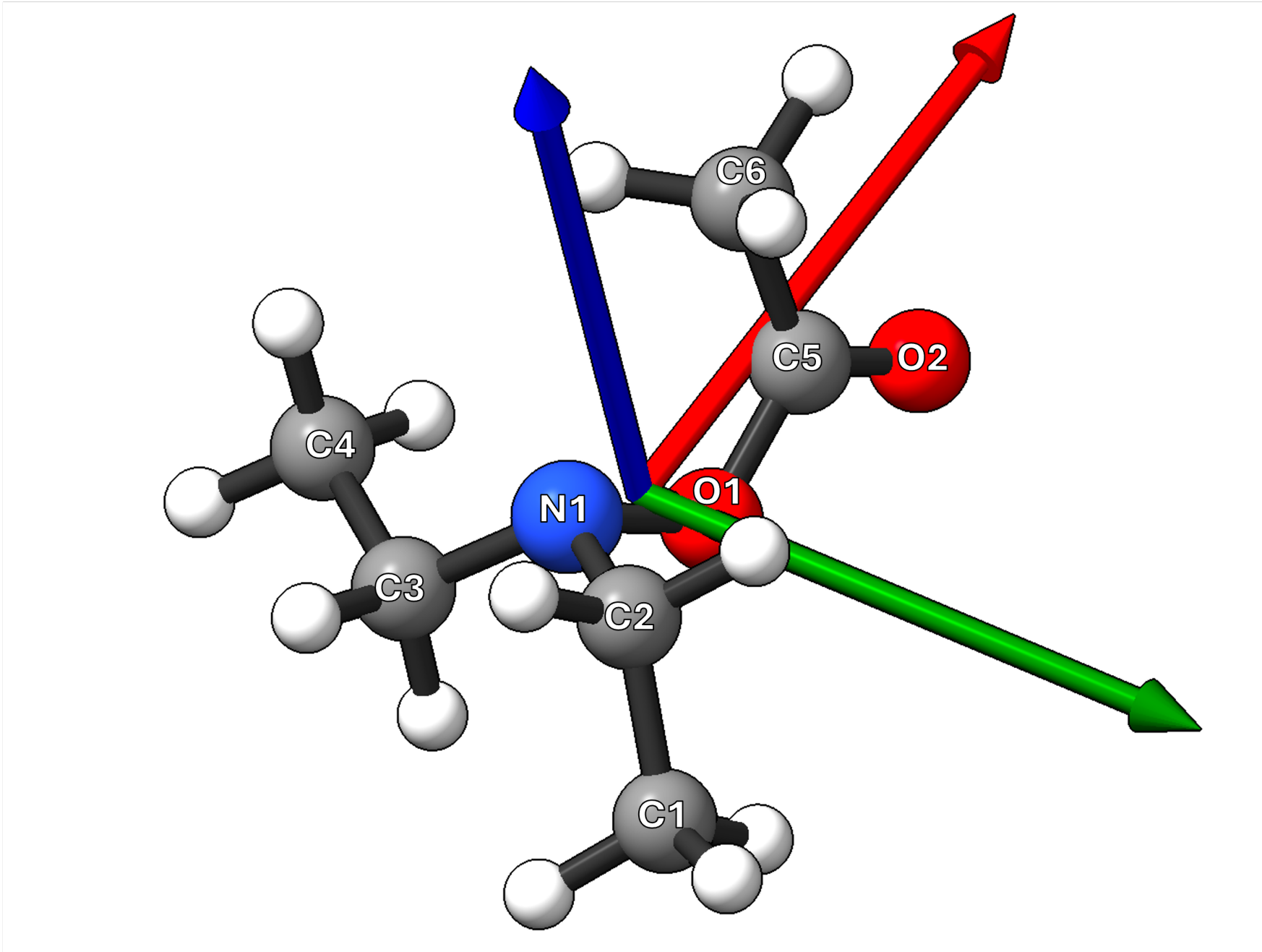


| atom | $a$/Å | $b$/Å | $c$/Å |
|---|---|---|---|
| C1 | −2.2628 | −1.5958 | 0.4267 |
| C2 | −1.4992 | −1.0034 | −0.7507 |
| N1 | −0.6428 | 0.1524 | −0.4596 |
| C3 | −1.3620 | 1.2472 | 0.1912 |
| C4 | −0.4888 | 2.4829 | 0.2918 |
| O1 | 0.3041 | −0.3056 | 0.5377 |
| C5 | 1.6054 | −0.3389 | 0.1028 |
| O2 | 2.4411 | −0.6926 | 0.8985 |
| C6 | 1.8818 | 0.0612 | −1.3170 |
| H1 | −0.1789 | 2.8153 | −0.6978 |
| H2 | 0.3985 | 2.2756 | 0.8883 |
| H3 | −1.0351 | 3.2931 | 0.7735 |
| H4 | −2.2346 | 1.4433 | −0.4356 |
| H5 | −1.7147 | 0.9474 | 1.1832 |
| H6 | −3.0154 | −0.9091 | 0.8097 |
| H7 | −1.5811 | −1.8541 | 1.2347 |
| H8 | −2.7738 | −2.5031 | 0.1060 |
| H9 | −2.1852 | −0.6652 | −1.5301 |
| H10 | −0.8516 | −1.7604 | −1.1955 |
| H11 | 1.3010 | −0.5427 | −2.0101 |
| H12 | 1.5949 | 1.0970 | −1.4799 |
| H13 | 2.9448 | −0.0723 | −1.4908 |

## 2.2 #1:1 water complex

Table S8: Principal axis system structure of the observed #1:1 water complex of #1-DEAcA (MP2/aug-cc-pVTZ). The *a*, *b*, and *c* axes are color-coded following the RGB scheme: red, green, and blue.

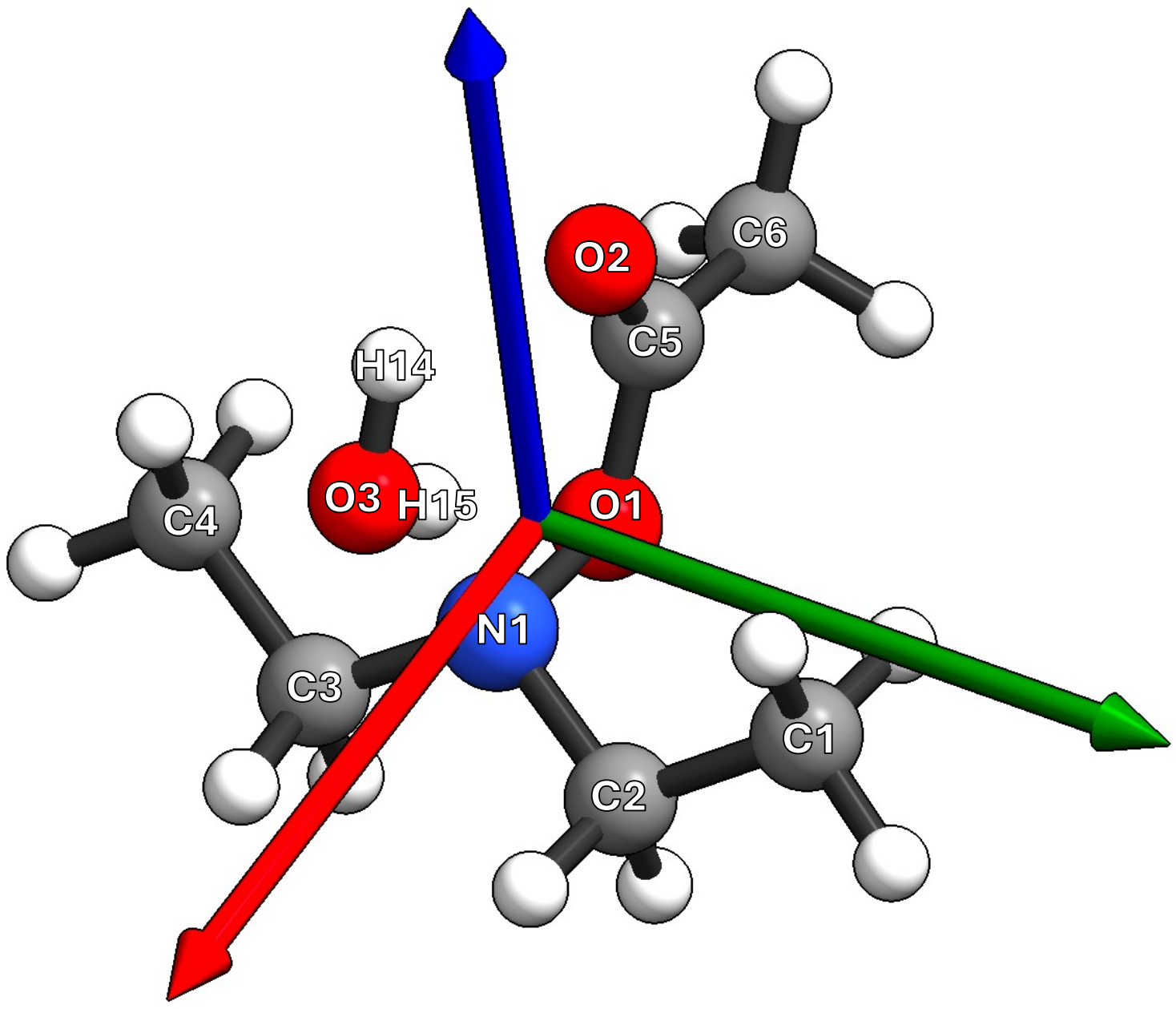


| atom | $a$/Å | $b$/Å | $c$/Å |
|---|---|---|---|
| C1 | 0.6435 | 2.4583 | 0.4163 |
| C2 | 1.1555 | 1.2125 | 1.1121 |
| N1 | 0.6320 | 0.0000 | 0.4819 |
| C3 | 1.1555 | −1.2125 | 1.1121 |
| C4 | 0.6435 | −2.4583 | 0.4163 |
| O1 | −0.7845 | 0.0000 | 0.7784 |
| C5 | −1.5590 | 0.0000 | −0.3498 |
| O2 | −1.1346 | 0.0000 | −1.4780 |
| C6 | −3.0030 | 0.0000 | 0.0618 |
| H1 | 1.0949 | 3.3442 | 0.8613 |
| H2 | 0.8977 | 2.4284 | −0.6417 |
| H3 | −0.4370 | 2.5456 | 0.5178 |
| H4 | 0.9104 | 1.2203 | 2.1817 |
| H5 | 2.2392 | 1.1421 | 1.0079 |
| H6 | 1.0949 | −3.3442 | 0.8613 |
| H7 | −0.4370 | −2.5456 | 0.5178 |
| H8 | 0.8977 | −2.4284 | −0.6417 |
| H9 | 2.2392 | −1.1421 | 1.0079 |
| H10 | 0.9104 | −1.2203 | 2.1817 |
| H11 | −3.6239 | 0.0000 | −0.8272 |
| H12 | −3.2135 | −0.8793 | 0.6676 |
| H13 | −3.2135 | 0.8793 | 0.6676 |
| O3 | 1.9578 | 0.0000 | −2.0587 |
| H14 | 1.2666 | 0.0000 | −1.3704 |
| H15 | 1.4559 | 0.0000 | −2.8786 |

Table S9: Principal axis system structure of the #1:1 (carbonyl) water complex of #1-DEAcA (MP2/aug-cc-pVTZ). The $a$, $b$, and $c$ axes are color-coded following the RGB scheme: red, green, and blue.

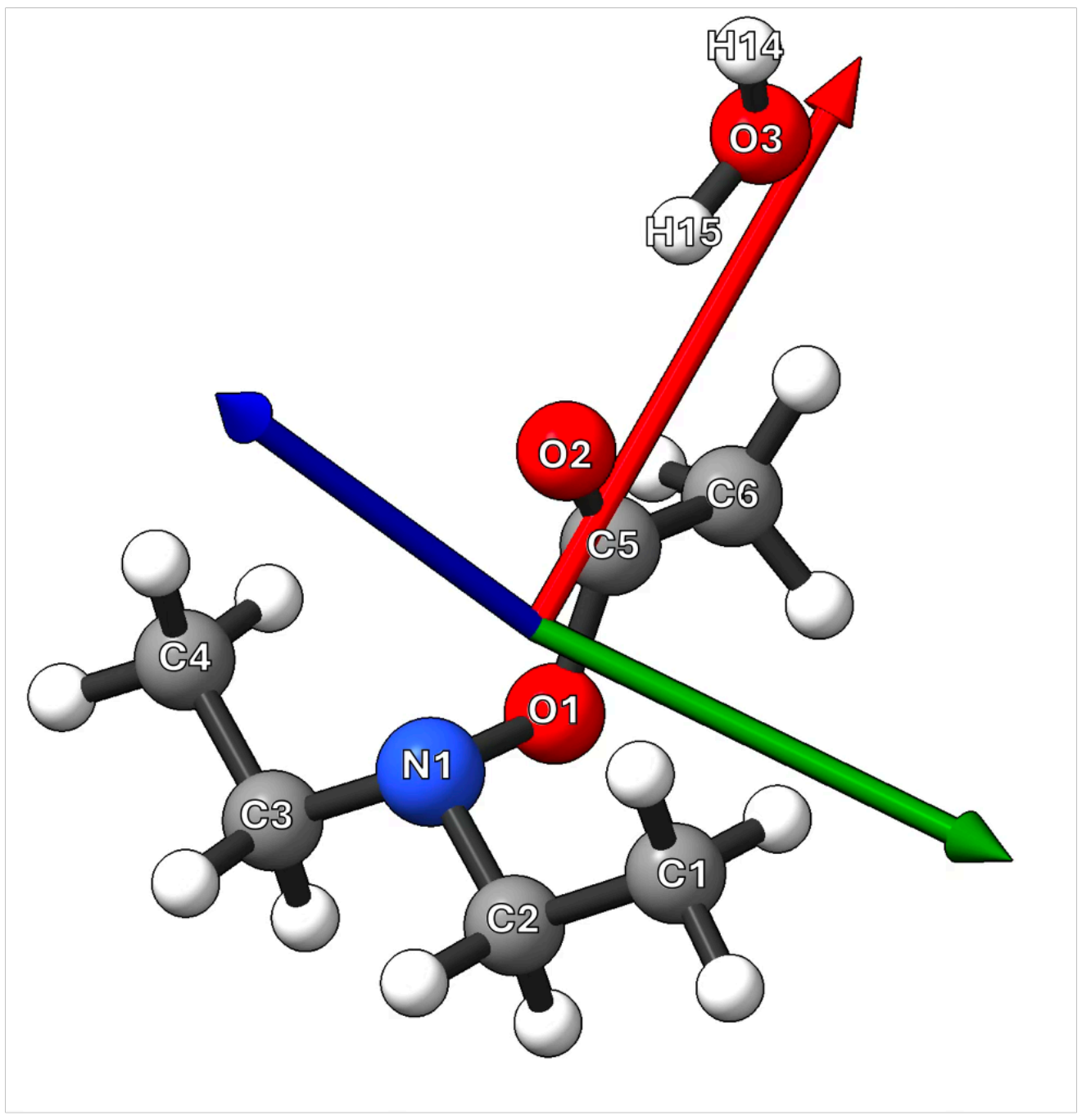


| atom | $a$/Å | $b$/Å | $c$/Å |
|---|---|---|---|
| C1 | −1.2480 | 2.4979 | −0.2646 |
| C2 | −2.1260 | 1.2771 | −0.0719 |
| N1 | −1.3792 | 0.0439 | −0.3098 |
| C3 | −2.2256 | −1.1369 | −0.1537 |
| C4 | −1.4491 | −2.4095 | −0.4297 |
| O1 | −0.4471 | −0.0330 | 0.8013 |
| C5 | 0.8559 | −0.0724 | 0.4117 |
| O2 | 1.2334 | −0.0256 | −0.7368 |
| C6 | 1.7268 | −0.2052 | 1.6273 |
| H1 | −2.1139 | −3.2716 | −0.3880 |
| H2 | −0.9892 | −2.3637 | −1.4150 |
| H3 | −0.6653 | −2.5548 | 0.3118 |
| H4 | −3.0261 | −1.0160 | −0.8853 |
| H5 | −2.6784 | −1.1581 | −0.8468 |
| H6 | −1.8405 | 3.4061 | −0.1609 |
| H7 | −0.7930 | 2.4819 | −1.2531 |
| H8 | −0.4549 | 2.5279 | 0.4805 |
| H9 | −2.5776 | 1.2676 | 0.9293 |
| H10 | −2.9324 | 1.2713 | −0.8070 |
| H11 | 2.7501 | 0.0492 | 1.3694 |
| H12 | 1.6907 | −1.2401 | 1.9667 |
| H13 | 1.3564 | 0.4254 | 2.4313 |
| O3 | 4.0651 | 0.0547 | −0.6948 |
| H14 | 4.5010 | 0.1871 | −1.5403 |
| H15 | 3.1168 | 0.0236 | −0.9050 |

Table S10: Principal axis system structure of the #1:1 (ester) water complex of #1-DEAcA (MP2/aug-cc-pVTZ). The $a$, $b$, and $c$ axes are color-coded following the RGB scheme: red, green, and blue.

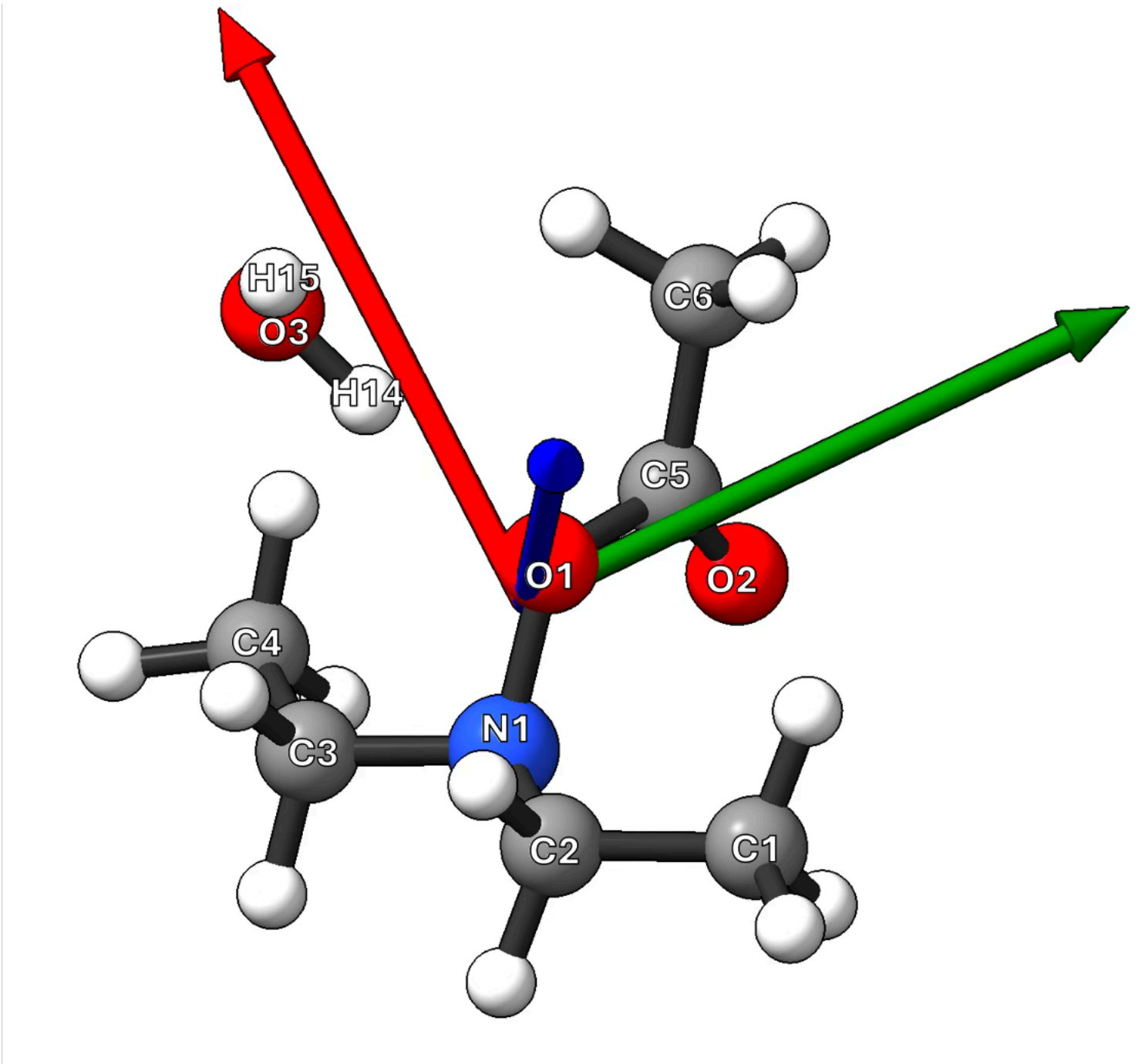


| atom | $a$/Å | $b$/Å | $c$/Å |
|---|---|---|---|
| C1 | −2.6142 | 0.5223 | −1.0487 |
| C2 | −1.8830 | −0.7790 | −0.7833 |
| N1 | −0.8904 | −0.6235 | 0.2762 |
| C3 | −0.2055 | −1.8835 | 0.5646 |
| C4 | 0.8408 | −1.7095 | 1.6478 |
| O1 | 0.1290 | 0.2336 | −0.3009 |
| C5 | 0.3394 | 1.3934 | 0.4211 |
| O2 | −0.2612 | 1.6978 | 1.4150 |
| C6 | 1.4242 | 2.1795 | −0.2577 |
| H1 | 0.3981 | −1.2448 | 2.5271 |
| H2 | 1.6656 | −1.0906 | 1.2988 |
| H3 | 1.2517 | −2.6791 | 1.9257 |
| H4 | −0.9920 | −2.5606 | 0.9019 |
| H5 | 0.2400 | −2.3070 | −0.3458 |
| H6 | −1.9343 | 1.2770 | −1.4403 |
| H7 | −3.4034 | 0.3668 | −1.7835 |
| H8 | −3.0552 | 0.8996 | −0.1281 |
| H9 | −1.4152 | −1.1657 | −1.6997 |
| H10 | −2.5781 | −1.5362 | −0.4171 |
| H11 | 1.6247 | 3.0728 | 0.3239 |
| H12 | 2.3240 | 1.5736 | −0.3507 |
| H13 | 1.1039 | 2.4542 | −1.2616 |
| O3 | 2.5236 | −0.8984 | −1.5006 |
| H14 | 1.6482 | −0.5574 | −1.2684 |
| H15 | 2.5244 | −0.9390 | −2.4610 |

## 2.3 #1:2 water complex

Table S11: Principal axis system structure of the observed #1:2 water complex of #1-DEAcA (MP2/aug-cc-pVTZ). The $a$, $b$, and $c$ axes are color-coded following the RGB scheme: red, green, and blue.

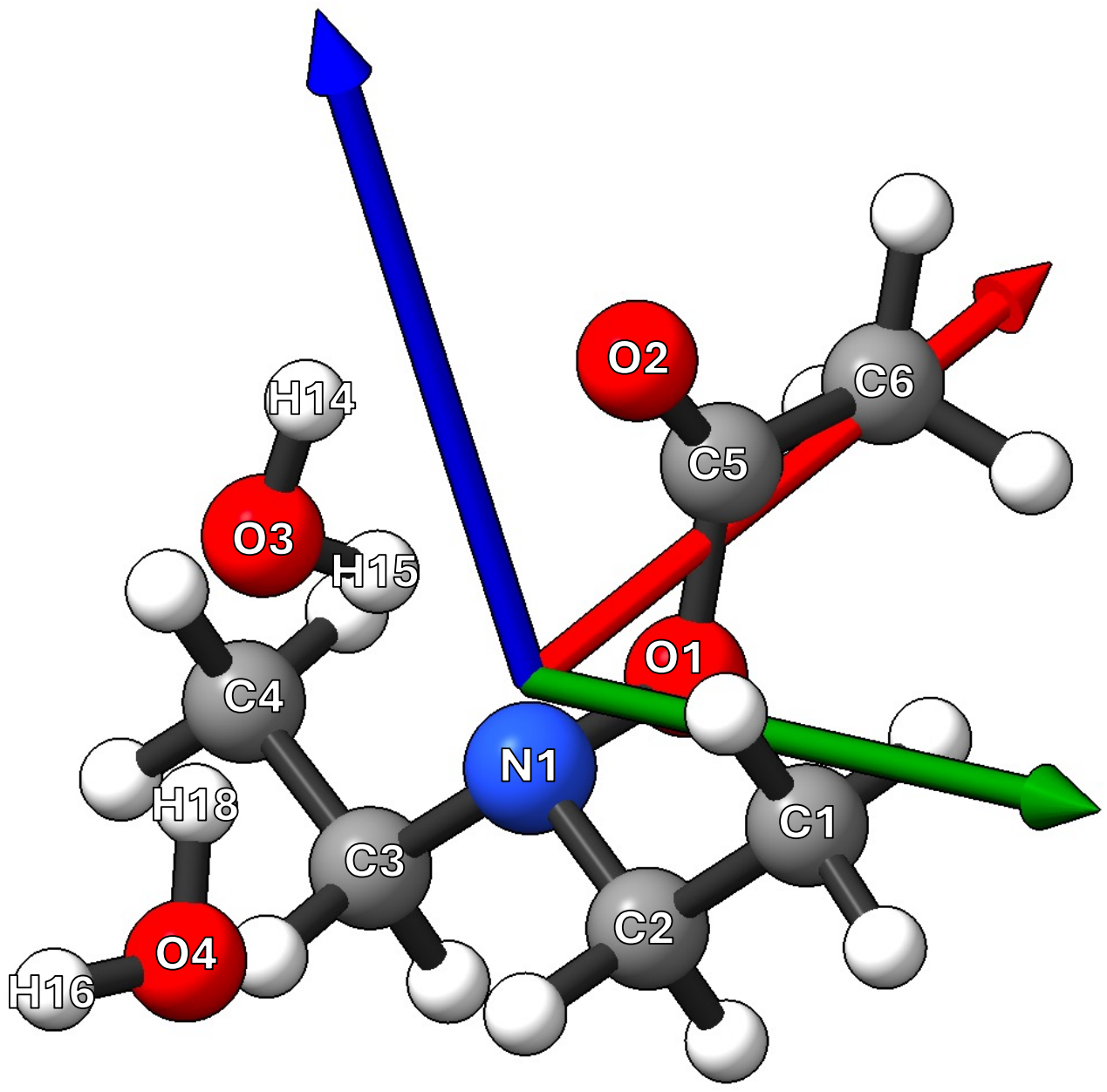


| atom | $a$/Å | $b$/Å | $c$/Å |
|---|---|---|---|
| C1 | −0.2357 | −1.2385 | 1.2161 |
| C2 | −0.6887 | 1.1810 | 1.2105 |
| N1 | −0.1947 | −0.0345 | 0.5427 |
| C3 | −0.6687 | −2.4954 | 0.4991 |
| C4 | −0.2036 | 2.4282 | 0.4853 |
| O1 | 1.2414 | 2.5144 | 0.4998 |
| C5 | 1.9456 | 0.8305 | 0.5756 |
| O2 | 1.4556 | −1.2387 | 2.2652 |
| C6 | 3.4124 | −1.1480 | 1.1826 |
| H1 | −0.5674 | 2.4123 | −0.5502 |
| H2 | 0.8490 | 3.3117 | 0.9706 |
| H3 | −0.6590 | 1.1106 | 1.1852 |
| H4 | −1.7753 | −0.9205 | 0.4119 |
| H5 | −0.3680 | 0.0940 | −1.0173 |
| H6 | 0.8821 | 1.1821 | 2.2565 |
| H7 | −0.5391 | −0.0522 | 0.7497 |
| H8 | −0.6167 | 0.0538 | −0.4133 |
| H9 | −1.7564 | 0.1669 | −1.5108 |
| H10 | −0.3380 | 0.0119 | −0.0951 |
| H11 | 3.9772 | −2.4796 | −0.5379 |
| H12 | 3.6667 | −2.5898 | 0.5213 |
| H13 | −1.7753 | −3.3726 | 0.9869 |
| O3 | −1.4991 | −0.1370 | −1.2812 |
| H14 | −1.0921 | 0.0592 | −2.7748 |
| H15 | −0.8010 | −0.2517 | −1.9616 |
| O4 | −3.5197 | −0.0050 | −0.4301 |
| H16 | −4.4052 | 0.2289 | −0.1402 |
| H17 | −2.9574 | 0.1008 | −0.9274 |

## 2.4 #1:3 water complex

Table S12: Principal axis system structure of the observed #1:3 water complex of #1-DEAcA (MP2/aug-cc-pVTZ). The $a$, $b$, and $c$ axes are color-coded following the RGB scheme: red, green, and blue.

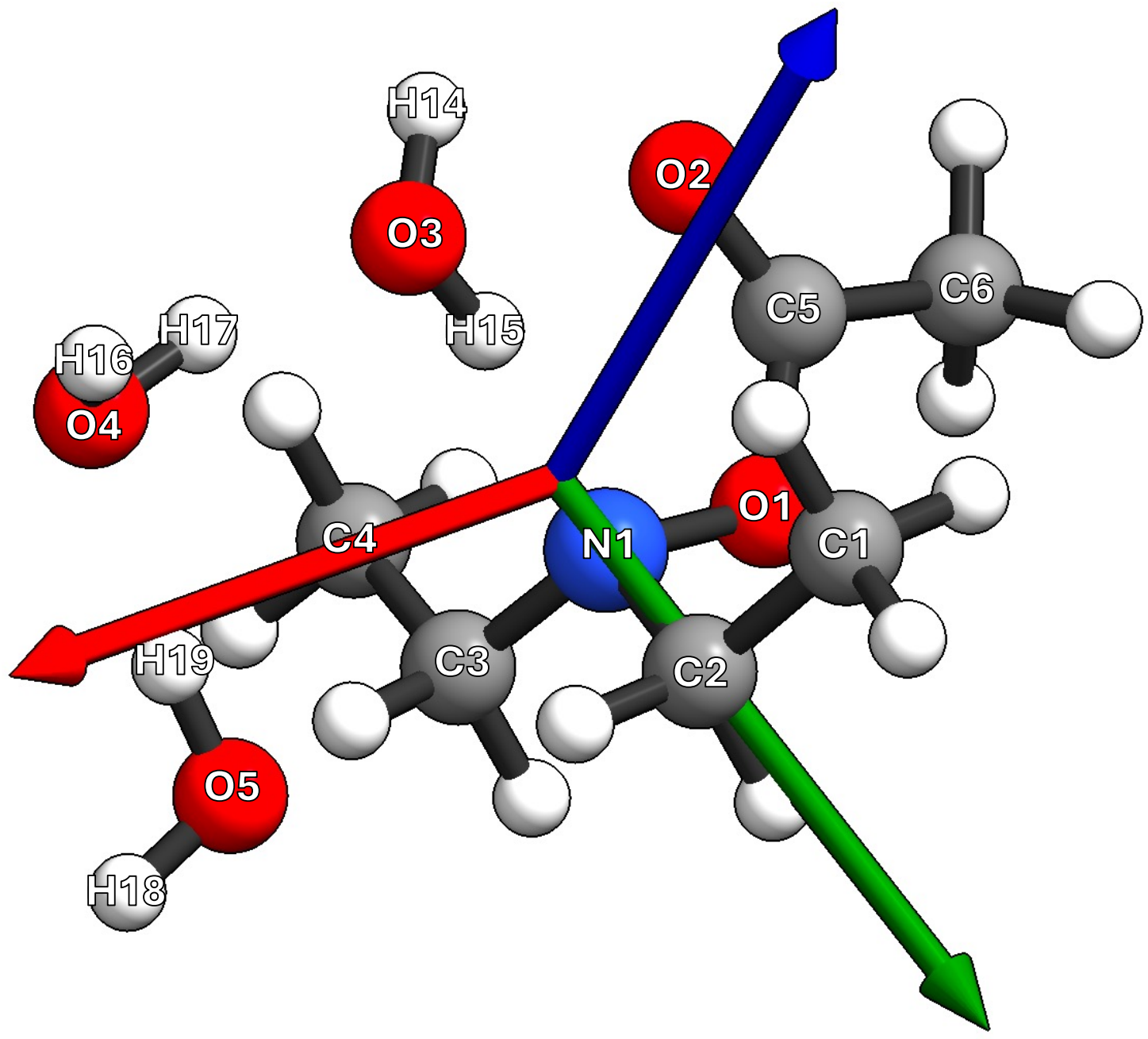


| atom | $a$/Å | $b$/Å | $c$/Å |
|---|---|---|---|
| C1 | 0.1884 | −1.4278 | 2.1383 |
| C2 | −0.2234 | −0.7687 | −1.5475 |
| N1 | −0.2394 | −0.5635 | −0.1665 |
| C3 | 0.2104 | −1.6681 | 0.6990 |
| C4 | −0.1730 | 0.3987 | −2.4352 |
| O1 | −1.6875 | 0.2419 | −3.4386 |
| C5 | −2.3406 | −0.4080 | 0.9115 |
| O2 | −1.8084 | −2.6129 | 0.3197 |
| C6 | −3.8194 | −1.7006 | 0.6016 |
| H1 | 0.1497 | 0.5011 | −2.5127 |
| H2 | 0.1872 | 1.3277 | −2.0425 |
| H3 | −1.3077 | −0.8688 | −1.4800 |
| H4 | 1.2745 | 1.1627 | 0.1222 |
| H5 | −0.2192 | −0.3173 | −0.8459 |
| H6 | 0.2211 | −1.7109 | −1.9300 |
| H7 | −1.2548 | −0.6786 | −0.2157 |
| H8 | 0.2369 | 0.4776 | 0.0889 |
| H9 | 1.2946 | 1.5305 | 0.3449 |
| H10 | −0.2006 | 0.2169 | 0.0606 |
| H11 | −4.0934 | −2.2339 | 2.7688 |
| H12 | −4.0872 | −0.4859 | 2.4989 |
| H13 | 1.2745 | −1.3986 | 2.2368 |
| O3 | 1.0486 | −1.7161 | −0.9153 |
| H14 | 0.4549 | −2.4728 | −0.9304 |
| H15 | 0.4844 | −1.0002 | −0.5418 |
| O4 | 3.6763 | −0.2872 | −0.1729 |
| H16 | 4.1541 | 0.2871 | 0.1729 |
| H17 | 2.7394 | −1.5192 | −0.8799 |
| O5 | 3.3828 | 1.0003 | 0.5418 |
| H18 | 4.1736 | −1.0002 | −0.5418 |
| H19 | 3.5895 | 2.4728 | 0.9304 |

## 2.5 #1:4 water complex

Table S13: Principal axis system structure of the observed #1:4 water complex of #1-DEAcA (MP2/aug-cc-pVTZ). The $a$, $b$, and $c$ axes are color-coded following the RGB scheme: red, green, and blue.

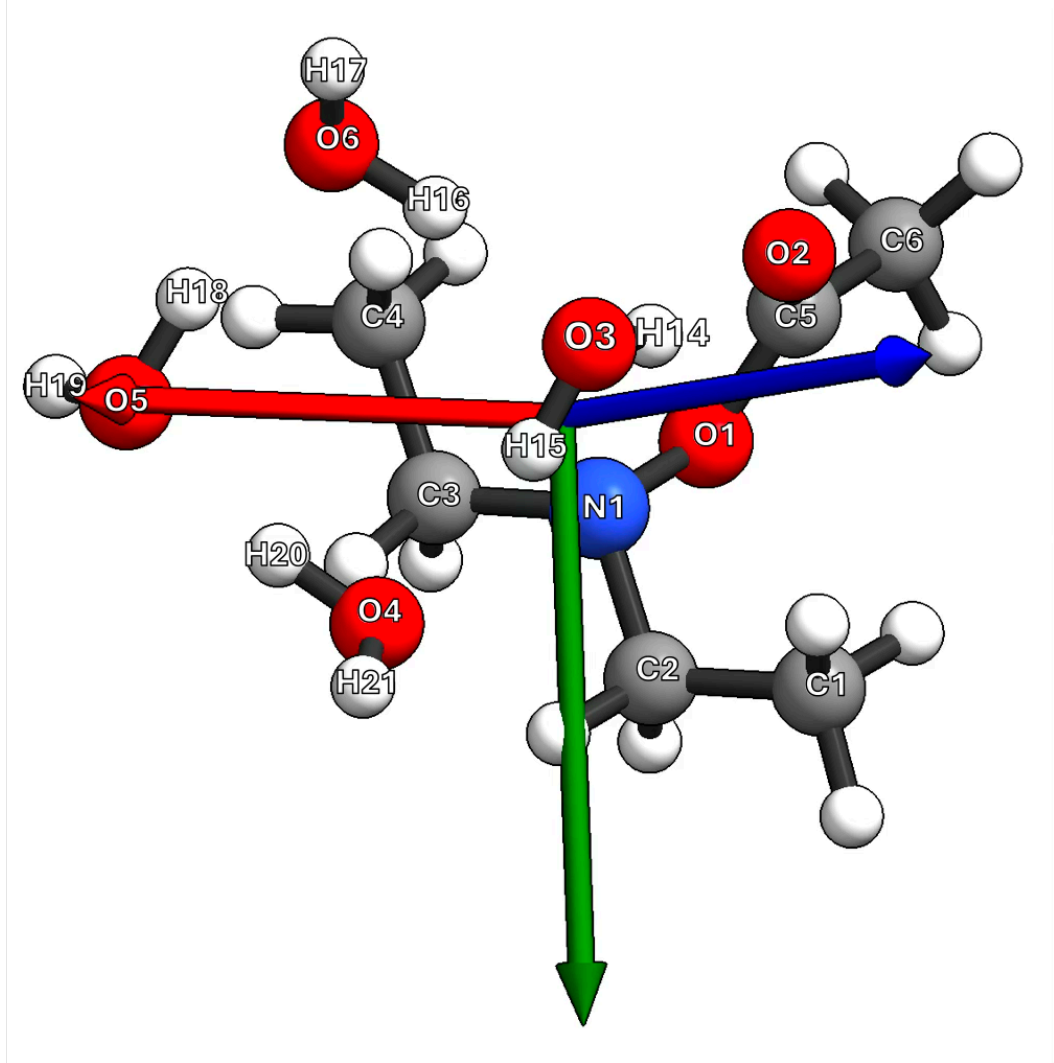


| atom | $a$/Å | $b$/Å | $c$/Å |
|---|---|---|---|
| C1 | 0.3650 | −0.9694 | 1.9462 |
| C2 | 0.0266 | 0.4910 | 1.7172 |
| N1 | −0.6967 | 0.6814 | 0.4611 |
| C3 | −0.9866 | 2.0937 | 0.2189 |
| C4 | −1.6836 | 2.2901 | −1.1132 |
| O1 | −1.9953 | 0.0664 | 0.6860 |
| C5 | −2.2667 | −0.9227 | −0.2048 |
| O2 | −1.5451 | −1.2598 | −1.1151 |
| C6 | −3.5918 | −1.5417 | 0.1334 |
| H1 | 0.9457 | −1.0766 | 2.8624 |
| H2 | −0.5394 | −1.5663 | 2.0575 |
| H3 | 0.9363 | 1.0824 | 1.6075 |
| H4 | −0.5524 | 0.9007 | 2.5559 |
| H5 | 0.9459 | −1.3675 | 1.1159 |
| H6 | −1.8352 | 3.3523 | −1.3021 |
| H7 | −2.6594 | 1.8063 | −1.1215 |
| H8 | −1.0824 | 1.8730 | −1.9198 |
| H9 | −1.5805 | 2.5083 | 1.0443 |
| H10 | −0.0106 | 2.5794 | 0.2122 |
| H11 | −3.9390 | −2.1209 | −0.7156 |
| H12 | −4.3157 | −0.7775 | 0.4037 |
| H13 | −3.4612 | −2.2017 | 0.9903 |
| O3 | 1.0893 | −0.3703 | −1.8413 |
| H14 | 0.1928 | −0.5061 | −1.4864 |
| H15 | 1.3878 | 0.4582 | −1.4274 |
| O4 | 2.3062 | 1.8186 | −0.4396 |
| H16 | 2.9308 | 1.2287 | 0.0366 |
| H17 | 2.8689 | 2.4023 | −0.9573 |
| O5 | 3.8972 | −0.0641 | 0.7358 |
| H18 | 3.5851 | −0.8743 | 0.2702 |
| H19 | 3.9052 | −0.2918 | 1.6691 |
| O6 | 2.7688 | −2.0748 | −0.6672 |
| H20 | 2.1216 | −1.5249 | −1.1810 |
| H21 | 3.2287 | −2.6096 | −1.3198 |

## 2.6 #2:4 water complex

Table S14: Principal axis system structure of #2:4 water complex of #2-DEAcA (MP2/aug-cc-pVTZ). The $a$, $b$, and $c$ axes are color-coded following the RGB scheme: red, green, and blue.

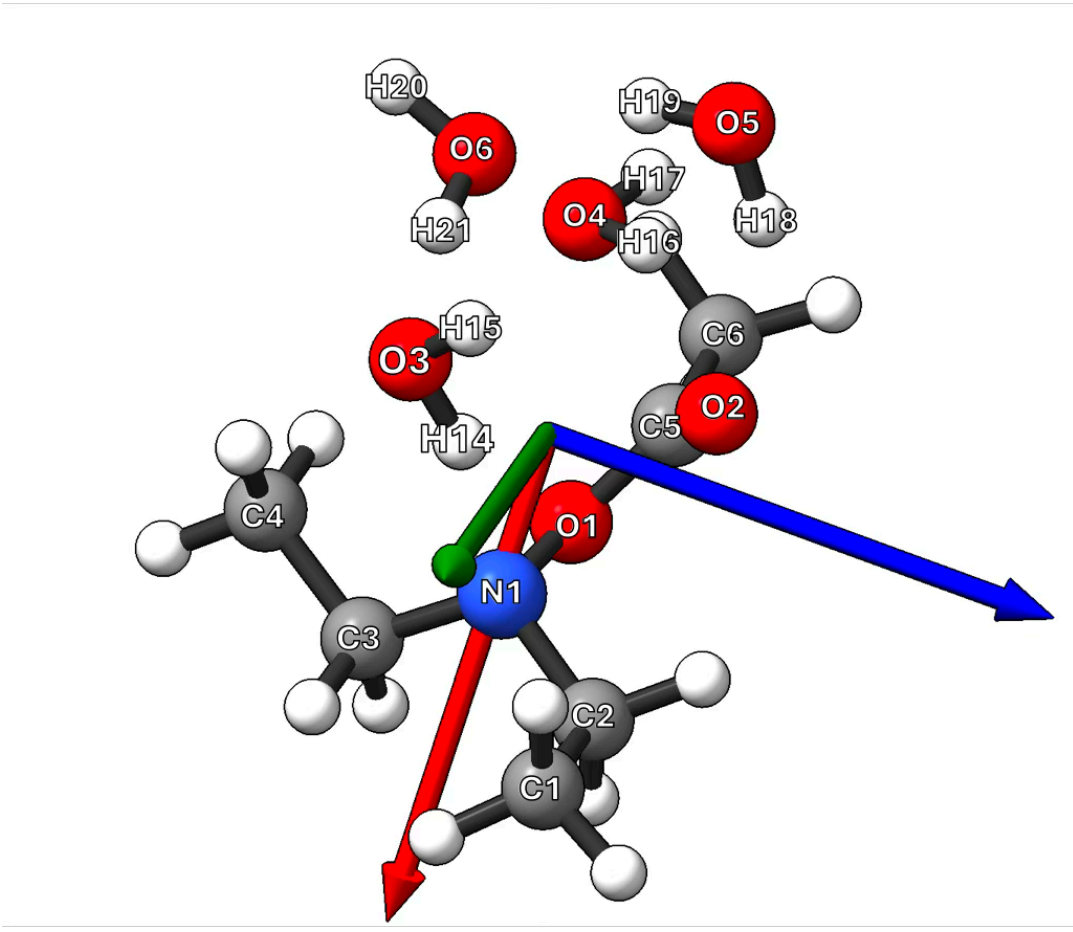


| atom | $a$/Å | $b$/Å | $c$/Å |
|---|---|---|---|
| C1 | 1.8444 | −0.2860 | 2.3618 |
| C2 | 2.5401 | 0.0881 | 1.0667 |
| N1 | 1.6056 | 0.1629 | −0.0625 |
| C3 | 2.2878 | 0.5640 | −1.3016 |
| C4 | 2.5354 | 2.0632 | −1.3059 |
| O1 | 1.2150 | −1.2287 | −0.2407 |
| C5 | 0.0096 | −1.3596 | −0.8514 |
| O2 | −0.5912 | −0.4361 | −1.3581 |
| C6 | −0.4499 | −2.7812 | −0.7832 |
| H1 | 3.2526 | 2.3677 | −0.5465 |
| H2 | 2.9339 | 2.3592 | −2.2754 |
| H3 | 1.6023 | 2.5989 | −1.1387 |
| H4 | 3.2206 | −0.0008 | −1.4185 |
| H5 | 1.6205 | 0.3074 | −2.1210 |
| H6 | 0.3896 | −3.4651 | −0.7012 |
| H7 | −1.0702 | −2.8647 | 0.1098 |
| H8 | −1.0536 | −3.0076 | −1.6568 |
| H9 | 2.9766 | 1.0830 | 1.1488 |
| H10 | 3.3448 | −0.6186 | 0.8279 |
| H11 | 2.5760 | −0.3241 | 3.1682 |
| H12 | 1.0828 | 0.4491 | 2.6129 |
| H13 | 1.3736 | −1.2632 | 2.2801 |
| H14 | −3.1500 | 2.9825 | −0.8152 |
| O3 | −2.9468 | 2.3828 | −0.0932 |
| H15 | −3.2350 | 1.4938 | −0.3962 |
| H16 | −3.1088 | −0.5979 | 0.1705 |
| O4 | −3.3738 | −0.2613 | −0.7049 |
| H17 | −2.5570 | −0.3838 | −1.2122 |
| H18 | −2.2293 | −1.0132 | 2.5397 |
| O5 | −1.9212 | −0.9407 | 1.6319 |
| H19 | −1.4682 | −0.0622 | 1.5840 |
| H20 | −0.0061 | 1.1421 | 0.5791 |
| O6 | −0.6887 | 1.4307 | 1.2139 |
| H21 | −1.3624 | 1.9083 | 0.6921 |

# 3 Experimental and ab initio spectroscopic parameters of the conformers of DEAcA.

Table S15: Experimental (S-reduction, $III^{\ell}$-representation) and theoretical (MP2/aug-cc-pVTZ) spectroscopic parameters for #1-DEAcA (aaAs) and its $^{13}$C1 and $^{13}$C2 isotopologues.

| Parameter | MP2 | Exp. | $^{13}$C1 | $^{13}$C2 |
|---|---|---|---|---|
| $A$ / MHz | 1734.11 | 1725.756570(85)[a] | 1691.0454(15) | 1717.2572(15) |
| $B$ / MHz | 1457.83 | 1445.783684(74) | 1441.8576(18) | 1434.4690(18) |
| $C$ / MHz | 964.51 | 953.26275(21) | 941.0354(14) | 945.9759(14) |
| $D_J$ / kHz | 3.3045 | 1.06184(42) | [ 1.06184][b] | [ 1.06184] |
| $D_{JK}$ / kHz | -2.7470 | -0.9920(19) | [-0.9920] | [-0.9920] |
| $D_K$ / kHz | -0.5131 | -0.0342(75) | [-0.0342] | [-0.0342] |
| $d_1$ / kHz | 0.0136 | 0.01062(35) | [ 0.01062] | [ 0.01062] |
| $d_2$ / kHz | 1.5802 | 0.43214(33) | [ 0.43214] | [ 0.43214] |
| 3/2 $\chi_{cc}$ / MHz | -7.4920 | -7.8028(24) | -7.872(16) | -7.788(17) |
| 1/4 $(\chi_{bb} - \chi_{aa})$ / MHz | -1.0035 | -1.01691(93) | -1.0095(54) | -1.0148(54) |
| $N^c$ | - | 396 | 18 | 19 |
| $N_{PJ-FTMW}$ | - | 98 | - | - |
| $N_{FJ-AMMW}$ | - | 148 | - | - |
| $N_{CP-FTMW}$ | - | 150 | 18 | 19 |
| $\sigma^d$ / kHz | - | 26 | 11 | 6 |
| $\sigma_{PJ-FTMW}$ / kHz | - | 3 | - | - |
| $\sigma_{FJ-AMMW}$ / kHz | - | 61 | - | - |
| $\sigma_{CP-FTMW}$ / kHz | - | 11 | 11 | 6 |
| $\mu_a$ / D | -0.98 | Y[e] | Y | Y |
| $\mu_b$ / D | 0.00 | N | N | N |
| $\mu_c$ / D | 2.40 | Y | Y | Y |
| $P_{aa}$ / uÅ$^2$ | 289.60 | 293.432933(60) | 294.34759(48) | 296.12872(48) |
| $P_{bb}$ / uÅ$^2$ | 234.37 | 236.724152(60) | 242.69807(48) | 238.11217(48) |
| $P_{cc}$ / uÅ$^2$ | 57.06 | 56.120742(60) | 56.15790(48) | 56.18212(48) |
| $\kappa$ | 0.281999 | 0.27514531(31) | 0.3355080(56) | 0.2667055(53) |
| $\chi_{aa}$ / MHz | 4.5043 | 4.6348(20) | 4.643(12) | 4.626(12) |
| $\chi_{bb}$ / MHz | 0.4904 | 0.5671(20) | 0.605(12) | 0.566(12) |
| $\chi_{cc}$ / MHz | -4.9947 | -5.2019(16) | -5.248(11) | -5.192(11) |
| $E_e$ / a.u. | -440.813824 | - | - | - |
| $E_0$ / a.u. | -440.622101 | - | - | - |

[a] Error in the unit of the last digit; [b] Values in square brackets are fixed to those of the normal species; [c] Total number of fitted transition lines; the contributions from each spectrometer are reported in the rows below; [d] Standard deviation of the global fit; the values for each spectrometer are reported in the rows below; [e] Y/N indicate whether that transition type has been observed.

Table S16: Experimental (S-reduction, $III^{\ell}$-representation) and theoretical (MP2/aug-cc-pVTZ) parameters of #1'-DEAcA (aaAa).

| Parameter | MP2 | Exp. |
|---|---|---|
| $A$ / MHz | 1699.30 | 1682.81373(58)[a] |
| $B$ / MHz | 1479.52 | 1463.58151(62) |
| $C$ / MHz | 978.54 | 966.35699(35) |
| $D_J$ / kHz | 0.6100 | - |
| $D_K$ / kHz | 0.2845 | - |
| $D_{JK}$ / kHz | -0.8550 | - |
| $d_1$ / kHz | 0.0132 | - |
| $d_2$ / kHz | 0.2405 | - |
| 3/2 $\chi_{cc}$ / MHz | -6.0966 | -6.3896(81) |
| 1/4 $(\chi_{bb} - \chi_{aa})$ / MHz | -0.7937 | -0.8030(42) |
| $N^b$ | - | 45 |
| $N_{PJ-FTMW}$ | - | 8 |
| $N_{CP-FTMW}$ | - | 37 |
| $\sigma^c$ / kHz | - | 31 |
| $\sigma_{PJ-FTMW}$ / kHz | - | 26 |
| $\sigma_{CP-FTMW}$ / kHz | - | 35 |
| $\mu_a$ / D | -3.57 | Y[d] |
| $\mu_b$ / D | -0.00 | N |
| $\mu_c$ / D | -2.24 | Y |
| $P_{aa}$ / uÅ$^2$ | 280.32 | 283.97925(13) |
| $P_{bb}$ / uÅ$^2$ | 236.14 | 238.99415(13) |
| $P_{cc}$ / uÅ$^2$ | 61.26 | 61.32370(13) |
| $\kappa$ | 0.390153 | 0.3880099(21) |
| $\chi_{aa}$ / MHz | 3.6196 | 3.7359(88) |
| $\chi_{bb}$ / MHz | 0.4448 | 0.5239(88) |
| $\chi_{cc}$ / MHz | -4.0644 | -4.2597(54) |
| $E_e$ / a.u. | -440.812830 | - |
| $E_0$ / a.u. | -440.620381 | - |

[a] Error in the unit of the last digit; [b] Total number of fitted transition lines; the contributions from each spectrometer are reported in the rows below; [c] Standard deviation of the global fit; the values for each spectrometer are reported in the rows below; [d] Y/N indicate whether that transition type has been observed.

Table S17: Experimental (S-reduction, $I^r$-representation) and theoretical (MP2/aug-cc-pVTZ) parameters of #2-DEAcA (gaAs).

| Parameter | MP2 | Exp. |
|---|---|---|
| $A$ / MHz | 2029.94 | 2023.06373(86)[a] |
| $B$ / MHz | 1194.84 | 1182.41101(72) |
| $C$ / MHz | 862.57 | 858.26207(73) |
| $D_J$ / kHz | 0.0983 | 0.250(26) |
| $D_{JK}$ / kHz | 0.8090 | 1.038(60) |
| $D_K$ / kHz | -0.2347 | - |
| $d_1$ / kHz | -0.0490 | -0.106(15) |
| $d_2$ / kHz | -0.0228 | -0.0258(45) |
| 3/2 $\chi_{aa}$ / MHz | 7.9451 | 8.1685(64)) |
| 1/4 $(\chi_{bb} - \chi_{cc})$ / MHz | 1.5689 | 1.6366(19) |
| $N^b$ | - | 141 |
| $\sigma^c$ / kHz | - | 9 |
| $\mu_a$ / D | -0.69 | Y[d] |
| $\mu_b$ / D | -1.21 | Y |
| $\mu_c$ / D | 2.01 | Y |
| $P_{aa}$ / uÅ$^2$ | 379.95 | 383.22260(29) |
| $P_{bb}$ / uÅ$^2$ | 205.95 | 205.61736(29) |
| $P_{cc}$ / uÅ$^2$ | 43.01 | 44.19138(29) |
| $\kappa$ | -0.430733 | -0.4434264(16) |
| $\chi_{aa}$ / MHz | 5.2967 | 5.4457(43) |
| $\chi_{bb}$ / MHz | 0.4895 | 0.5504(44) |
| $\chi_{cc}$ / MHz | -5.7862 | -5.9961(44) |
| $E_e$ / a.u. | -440.812466 | - |
| $E_0$ / a.u. | -440.620380 | - |

[a] Error in the unit of the last digit; [b] Number of transition lines recorded with the CP-FTMW spectrometer and included in the fit; [c] Standard deviation of the fit; [d] Y/N indicate whether that transition type has been observed.

Table S18: Experimental (S-reduction, $I^r$-representation) and theoretical (MP2/aug-cc-pVTZ) parameters of #3-DEAcA (agAs).

| Parameter | MP2 | Exp. |
|---|---|---|
| $A$ / MHz | 1904.60 | 1889.77526(84)[a] |
| $B$ / MHz | 1253.12 | 1245.01144(66) |
| $C$ / MHz | 946.51 | 937.99194(59) |
| $D_J$ / kHz | 0.0613 | - |
| $D_{JK}$ / kHz | 2.1325 | - |
| $D_K$ / kHz | -1.9194 | - |
| $d_1$ / kHz | -0.0325 | - |
| $d_2$ / kHz | -0.0294 | - |
| 3/2 $\chi_{aa}$ / MHz | 4.9387 | 5.183(14) |
| 1/4 $(\chi_{bb} - \chi_{cc})$ / MHz | 0.6371 | 0.6809(45) |
| $N^b$ | - | 42 |
| $\sigma^c$ / kHz | - | 17 |
| $\mu_a$ / D | -1.26 | Y[d] |
| $\mu_b$ / D | 0.06 | N |
| $\mu_c$ / D | 2.33 | Y |
| $P_{aa}$ / uÅ$^2$ | 335.95 | 338.64165(21) |
| $P_{bb}$ / uÅ$^2$ | 197.99 | 200.14657(21) |
| $P_{cc}$ / uÅ$^2$ | 67.35 | 67.28153(21) |
| $\kappa$ | -0.359963 | -0.3548542(17) |
| $\chi_{aa}$ / MHz | 3.2925 | 3.4555(91) |
| $\chi_{bb}$ / MHz | -0.3721 | -0.366(10) |
| $\chi_{cc}$ / MHz | -2.9204 | -3.090(10) |
| $E_e$ / a.u. | -440.811198 | - |
| $E_0$ / a.u. | -440.619110 | - |

[a] Error in the unit of the last digit; [b] Number of transition lines recorded with the CP-FTMW spectrometer and included in the fit; [c] Standard deviation of the fit; [d] Y/N indicate whether that transition type has been observed.

Table S19: Theoretical (MP2/aug-cc-pVTZ) parameters of #2'-DEAcA (gaAa) and #3'-DEAcA (agAa).

| Parameter | #2' | #3' |
|---|---|---|
| $A$ / MHz | 2106.83 | 1952.04 |
| $B$ / MHz | 1141.37 | 1242.75 |
| $C$ / MHz | 912.53 | 961.10 |
| $D_J$ / kHz | 0.1187 | 0.0967 |
| $D_K$ / kHz | 0.5696 | -0.6839 |
| $D_{JK}$ / kHz | 1.0459 | 1.4424 |
| $d_1$ / kHz | -0.0429 | -0.0381 |
| $d_2$ / kHz | -0.0047 | -0.0188 |
| 3/2 $\chi_{aa}$ / MHz | 6.6537 | 1.8754 |
| 1/4 $(\chi_{bb} - \chi_{cc})$ / MHz | 1.2910 | 0.5680 |
| $\mu_a$ / D | -3.93 | -3.20 |
| $\mu_b$ / D | 0.04 | 1.10 |
| $\mu_c$ / D | -1.99 | -2.52 |
| $P_{aa}$ uÅ$^2$ | 378.37 | 336.80 |
| $P_{bb}$ uÅ$^2$ | 175.46 | 189.03 |
| $P_{cc}$ uÅ$^2$ | 64.42 | 69.86 |
| $\kappa$ | -0.616780 | -0.431555 |
| $\chi_{aa}$ / MHz | 4.4358 | 1.2503 |
| $\chi_{bb}$ / MHz | 0.3641 | 0.5109 |
| $\chi_{cc}$ / MHz | -4.7998 | -1.7611 |
| $E_e$ / a.u. | -440.810911 | -440.810102 |
| $E_0$ / a.u. | -440.618630 | -440.617723 |

# 4 Experimental and ab initio spectroscopic parameters for the #1:1 water complex

Table S20: Experimental (S-reduction, $I^r$-representation) and theoretical (MP2/aug-cc-pVTZ) parameters of the #1:1 water complex of #1-DEAcA.

| Parameter | MP2 | Exp. |
|---|---|---|
| $A$ / MHz | 1225.56 | 1197.72315(39)[a] |
| $B$ / MHz | 994.70 | 976.77032(40) |
| $C$ / MHz | 893.37 | 886.78244(41) |
| $D_J$ / kHz | - | 0.263(13) |
| 3/2 $\chi_{aa}$ / MHz | 9.5095 | 9.7029(60) |
| 1/4 $(\chi_{bb} - \chi_{cc})$ / MHz | 1.7654 | 1.8383(21) |
| $N^b$ | - | 95 |
| $\sigma^c$ / kHz | - | 15 |
| $\mu_a$ / D | -2.25 | Y[d] |
| $\mu_b$ / D | 0.00 | N |
| $\mu_c$ / D | 3.04 | Y |
| $P_{aa}$ / uÅ$^2$ | 330.70 | 332.67506(18) |
| $P_{bb}$ / uÅ$^2$ | 235.00 | 237.22684(18) |
| $P_{cc}$ / uÅ$^2$ | 177.37 | 184.72293(18) |
| $\kappa$ | -0.389895 | -0.4211895(33) |
| $\chi_{aa}$ / MHz | 6.3397 | 6.4686(40) |
| $\chi_{bb}$ / MHz | 0.3610 | 0.4424(46) |
| $\chi_{cc}$ / MHz | -6.7008 | -6.9110(46) |
| $E_e$ / a.u. | -517.159041 | - |

[a] Error in the unit of the last digit; [b] Number of transition lines recorded with the CP-FTMW spectrometer and included in the fit; [c] Standard deviation of the fit; [d] Y/N indicate whether that transition type has been observed.

Table S21: Theoretical (MP2/aug-cc-pVTZ) parameters of the #1:1 (carbonyl) and #1:1 (ester) water complexes of #1-DEAcA.

| Parameter | #1:1 (carbonyl) | #1:1 (ester) |
|---|---|---|
| $A$ / MHz | 1567.64 | 1141.74 |
| $B$ / MHz | 729.75 | 1026.23 |
| $C$ / MHz | 601.47 | 824.90 |
| $(3/2)\chi_{aa}$ / MHz | 1.8699 | 3.8142 |
| $(1/4)(\chi_{bb} - \chi_{cc})$ / MHz | 0.5369 | 1.2735 |
| $\mu_a$ / D | -3.08 | -0.76 |
| $\mu_b$ / D | 0.04 | -0.77 |
| $\mu_c$ / D | -0.70 | -2.78 |
| $P_{aa}$ uÅ$^2$ | 605.20 | 331.24 |
| $P_{bb}$ uÅ$^2$ | 235.04 | 281.42 |
| $P_{cc}$ uÅ$^2$ | 87.34 | 161.22 |
| $\kappa$ | -0.734454 | 0.270866 |
| $\chi_{aa}$ / MHz | 1.2466 | 2.5428 |
| $\chi_{bb}$ / MHz | 0.4505 | 1.2756 |
| $\chi_{cc}$ / MHz | -1.6971 | -3.8184 |
| $E_e$ / a.u. | -517.154236 | -517.151489 |

# 5 Experimental and ab initio spectroscopic parameters of the #1:2 water complex

Table S22: Experimental (S-reduction, $I^r$-representation) and theoretical (MP2/aug-cc-pVTZ) parameters for the #1:2 water complex of #1-DEAcA.

| Parameter | MP2 | Exp. |
|---|---|---|
| $A$ / MHz | 1200.92 | 1167.99532(45)[a] |
| $B$ / MHz | 668.33 | 655.01417(36) |
| $C$ / MHz | 623.12 | 620.96580(34) |
| $D_J$ / kHz | - | 0.1181(58) |
| 3/2 $\chi_{aa}$ / MHz | 9.5745 | 9.7834(91) |
| 1/4 $(\chi_{bb} - \chi_{cc})$ / MHz | 1.6896 | 1.7807(27) |
| $N^b$ | - | 155 |
| $\sigma^c$ / kHz | - | 11 |
| $\mu_a$ / D | 1.89 | Y[d] |
| $\mu_b$ / D | 0.10 | N |
| $\mu_c$ / D | -1.16 | Y |
| $P_{aa}$ / uÅ$^2$ | 573.20 | 576.36235(32) |
| $P_{bb}$ / uÅ$^2$ | 237.84 | 237.49728(32) |
| $P_{cc}$ / uÅ$^2$ | 182.98 | 195.19195(32) |
| $\kappa$ | -0.843513 | -0.8755154(18) |
| $\chi_{aa}$ / MHz | 6.3830 | 6.5223(61) |
| $\chi_{bb}$ / MHz | 0.1877 | 0.3002(61) |
| $\chi_{cc}$ / MHz | -6.5707 | -6.8225(61) |
| $E_e$ / a.u. | -593.504804 | - |

[a] Error in the unit of the last digit; [b] Number of transition lines recorded with the CP-FTMW spectrometer and included in the fit; [c] Standard deviation of the fit; [d] Y/N indicate whether that transition type has been observed.

# 6 Experimental and ab initio spectroscopic parameters of the #1:3 water complex

Table S23: Experimental (S-reduction, $I^r$-representation) and theoretical (MP2/aug-cc-pVTZ) parameters for the #1:3 water complex of #1-DEAcA.

| Parameter | MP2 | Exp. |
|---|---|---|
| $A$ / MHz | 1016.04 | 999.477(19)[a] |
| $B$ / MHz | 455.81 | 445.10190(42) |
| $C$ / MHz | 444.80 | 434.86477(41) |
| $D_J$ / kHz | - | 0.0891(21) |
| $D_{JK}$ / kHz | - | 1.413(39) |
| $d_2$ / kHz | - | 0.0182(14) |
| 3/2 $\chi_{aa}$ / MHz | 9.4046 | 9.649(63) |
| 1/4 $(\chi_{bb} - \chi_{cc})$ / MHz | -1.1620 | -1.762(58) |
| $N$[b] | - | 102 |
| $\sigma$[c] / kHz | - | 11 |
| $\mu_a$ / D | 2.02 | Y[c] |
| $\mu_b$ / D | 0.94 | N |
| $\mu_c$ / D | -0.86 | N |
| $P_{aa}$ / uÅ$^2$ | 873.78 | 895.9659(49) |
| $P_{bb}$ / uÅ$^2$ | 262.43 | 266.1862(49) |
| $P_{cc}$ / uÅ$^2$ | 234.97 | 239.4572(49) |
| $\kappa$ | -0.961437 | -0.9637375(24) |
| $\chi_{aa}$ / MHz | 6.2697 | 6.433(42) |
| $\chi_{bb}$ / MHz | -5.4589 | -6.74(12) |
| $\chi_{cc}$ / MHz | -0.8109 | 0.31(12) |
| $E_e$ / a.u. | -669.848616 | - |

[a] Error in the unit of the last digit; [b] Number of transition lines recorded with the CP-FTMW spectrometer and included in the fit; [c] Standard deviation of the fit; [d] Y/N indicate whether that transition type has been observed.

# 7 Experimental and ab initio spectroscopic parameters of the #1:4 water complex

Table S24: Experimental (S-reduction, $I^r$-representation) and theoretical (MP2/aug-cc-pVTZ) parameters for the #1:4 water complex of #1-DEAcA.

| Parameter | MP2 | Exp. |
|---|---|---|
| $A$ / MHz | 795.56 | 783.79004(85)[a] |
| $B$ / MHz | 416.80 | 403.26052(29) |
| $C$ / MHz | 371.78 | 359.92882(36) |
| $D_J$ / kHz | - | 0.1191(26) |
| $D_{JK}$ / kHz | - | -0.115(18) |
| $D_K$ / kHz | - | 0.133(39) |
| 3/2 $\chi_{aa}$ / MHz | 6.1185 | 6.218(35) |
| 1/4 $(\chi_{bb} - \chi_{cc})$ / MHz | 1.0350 | 1.0594(43) |
| $N^b$ | - | 147 |
| $\sigma^c$ / kHz | - | 21 |
| $\mu_a$ / D | 1.29 | Y[d] |
| $\mu_b$ / D | -0.55 | N |
| $\mu_c$ / D | 2.37 | Y |
| $P_{aa}$ uÅ$^2$ | 968.32 | 1006.27573(91) |
| $P_{bb}$ uÅ$^2$ | 391.03 | 397.83245(91) |
| $P_{cc}$ uÅ$^2$ | 244.21 | 246.95630(91) |
| $\kappa$ | -0.787545 | -0.7955383(21) |
| $\chi_{aa}$ / MHz | 4.0790 | 4.145(23) |
| $\chi_{bb}$ / MHz | 0.0305 | 0.046(14) |
| $\chi_{cc}$ / MHz | -4.1096 | -4.191(14) |
| $E_e$ / a.u. | -746.195501 | - |

[a] Error in the unit of the last digit; [b] The number of transition lines fitted; [c] Number of transition lines recorded with the CP-FTMW spectrometer and included in the fit; [d] Y/N indicate whether that transition type has been observed.

# 8 Ab initio spectroscopic parameters for the #2:4 water complex

Table S25: Theoretical (MP2/aug-cc-pVTZ) parameters of the #2:4 water complex of #2-DEAcA.

| Parameter | MP2 |
|---|---|
| $A$ / MHz | 720.43 |
| $B$ / MHz | 450.37 |
| $C$ / MHz | 409.85 |
| 3/2 $\chi_{aa}$ / MHz | -6.0288 |
| 1/4 $(\chi_{bb} - \chi_{cc})$ / MHz | 0.9544 |
| $\mu_a$ / D | 4.17 |
| $\mu_b$ / D | -1.22 |
| $\mu_c$ / D | -0.83 |
| $P_{aa}$ uÅ$^2$ | 826.87 |
| $P_{bb}$ uÅ$^2$ | 406.22 |
| $P_{cc}$ uÅ$^2$ | 295.28 |
| $\kappa$ | -0.739074 |
| $\chi_{aa}$ / MHz | -4.0192 |
| $\chi_{bb}$ / MHz | 3.9184 |
| $\chi_{cc}$ / MHz | 0.1008 |
| $E_e$ / a.u. | -746.195679 |

## 9 Ab initio principal components of the $^{14}$N nuclear quadrupole tensor.

Table S26: Theoretical (MP2/aug-cc-pVTZ) $^{14}$N nuclear quadrupole coupling constants in the electric-field-gradient principal-axis system and the asymmetry parameter $\eta = (\chi_{xx} - \chi_{yy})/\chi_{zz}$ for the most stable conformers of DEAcA and its observed water complexes.

| Conformer | $\chi_{xx}$ / MHz | $\chi_{yy}$ / MHz | $\chi_{zz}$ / MHz | $\eta$ |
|---|---|---|---|---|
| #1 | 0.490 | 6.404 | -6.895 | 0.858 |
| #1’ | 0.445 | 6.160 | -6.605 | 0.865 |
| #2 | 0.425 | 6.399 | -6.824 | 0.875 |
| #2’ | 0.496 | 6.161 | -6.657 | 0.851 |
| #3 | 0.545 | 6.480 | -7.024 | 0.845 |
| #3’ | 0.617 | 6.236 | -6.853 | 0.820 |
| #1:1 water | 0.361 | 6.344 | -6.705 | 0.892 |
| #1:2 water | 0.187 | 6.403 | -6.590 | 0.943 |
| #1:3 water | 0.092 | 6.390 | -6.481 | 0.972 |
| #1:4 water | 0.322 | 6.576 | -6.897 | 0.907 |
| #2:4 water | 0.195 | 6.307 | -6.502 | 0.940 |

## 10 Kraitchman's substitution coordinates

Table S27: Principal axis system coordinates (in Å) of the ethyl carbon atoms of #1-DEAcA obtained with Kraitchman's substitution method ($r_s$) and ab initio calculations ($r_e$, MP2/aug-cc-pVTZ).

| | C1 | C2 |
|---|---|---|
| $\|a_s\|$ | 0.905(2) | 1.6249(9) |
| $\|b_s\|$ | 2.4688(6) | 1.208(1) |
| $\|c_s\|$ | 0.198(6) | 0.250(7) |
| $a_e$ | -0.892 | -1.622 |
| $b_e$ | ±2.456 | ±1.208 |
| $c_e$ | -0.211 | 0.245 |

# 11 Electron density topological analysis

Non-covalent interactions were explored by electron density topological analysis with Multiwfn on selected water clusters of DEAcA. The geometries and wavefunctions resulting from MP2/aug-cc-pVTZ level optimizations were used.

Intermolecular bond paths (yellow lines) and bond critical points (orange spheres) delineate primary connectivity, complemented by ring critical points (yellow spheres) for cyclic geometries and cage critical points (green spheres).

Critical points are related to troughs in the 2D plots of the reduced density gradient ($s$, dimensionless) against the electron density ($\rho$ in e/$a_0^3$) multiplied by the sign of the second derivative of the electron density along the second main axis of variation sign($\lambda_2$). Reduced electron density gradient isosurfaces ($s$=0.5) provide a visualization of the non-covalent interactions. A color-mapped scale distinguishes between strong attractive interactions (blue), weak van der Waals forces (green), and steric repulsion (red).

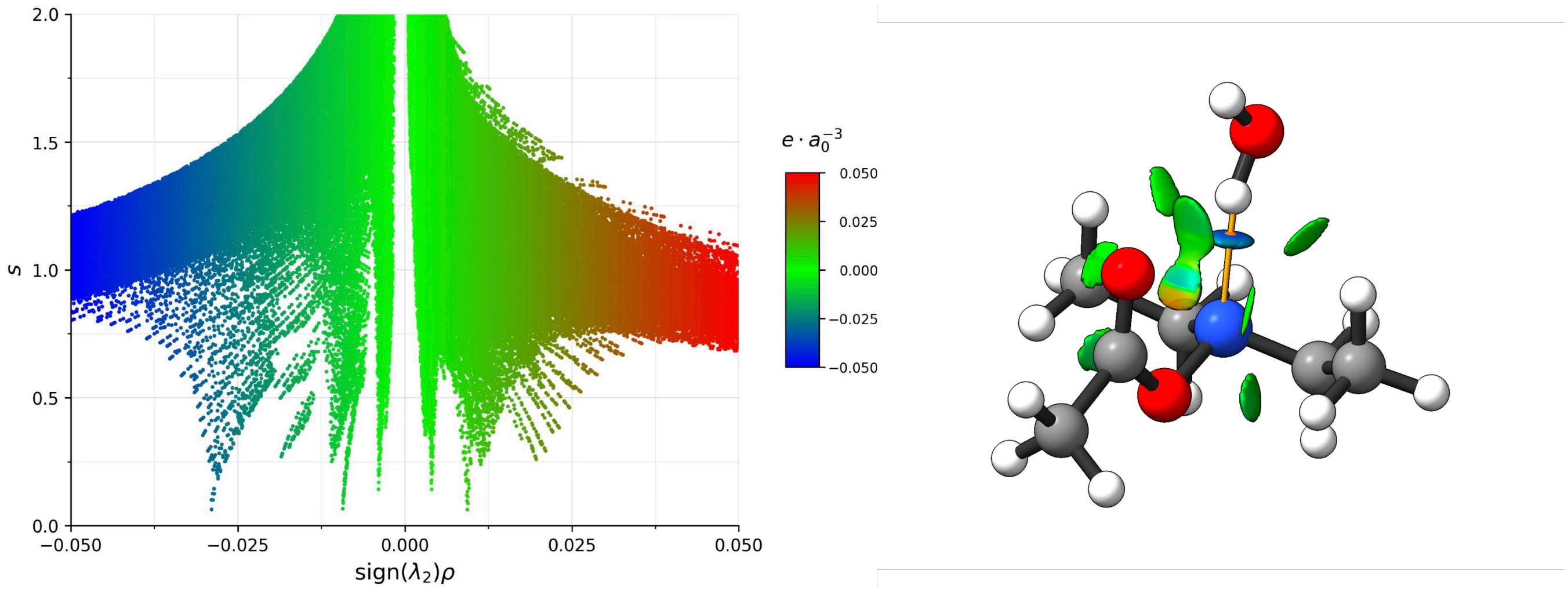


Figure S-II: Electron density topological analysis of #1:1 water complex of #1-DEAcA at the MP2/aug-cc-pVTZ level of calculation.

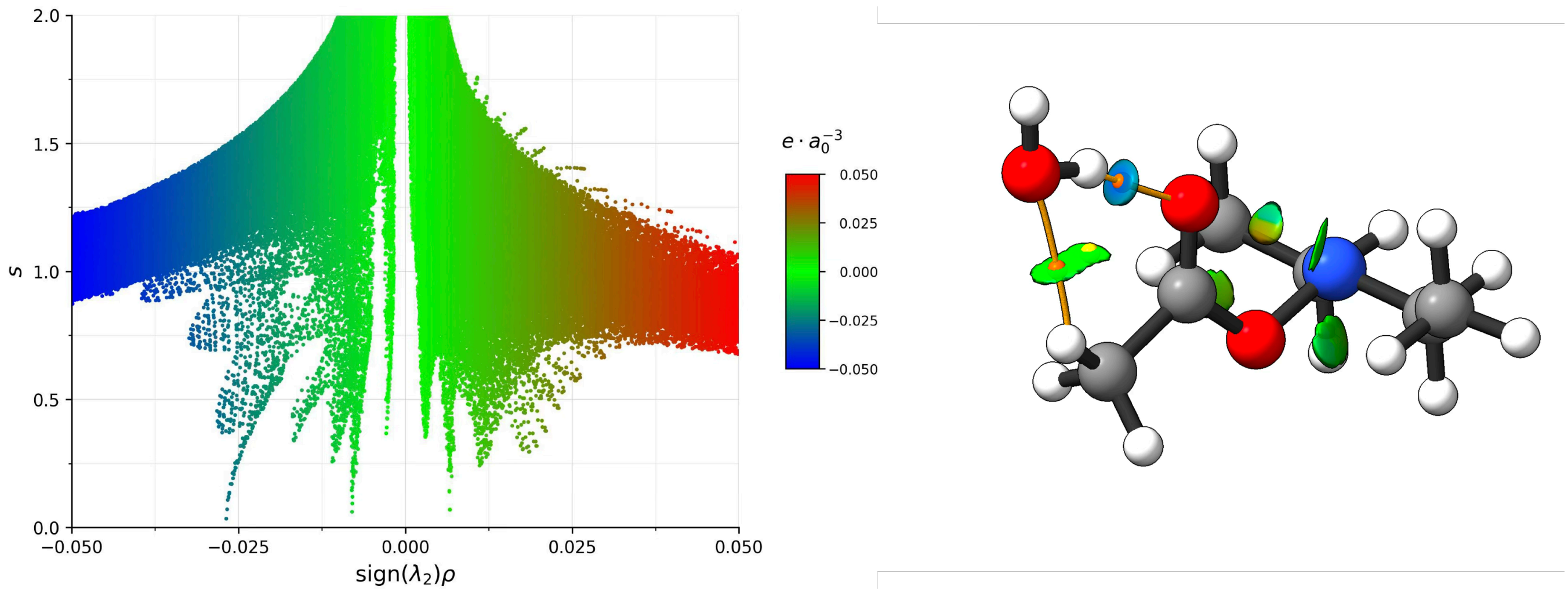


Figure S-III: Electron density topological analysis of #1:1 (carbonyl) water complex of #1-DEAcA at the MP2/aug-cc-pVTZ level of calculation.

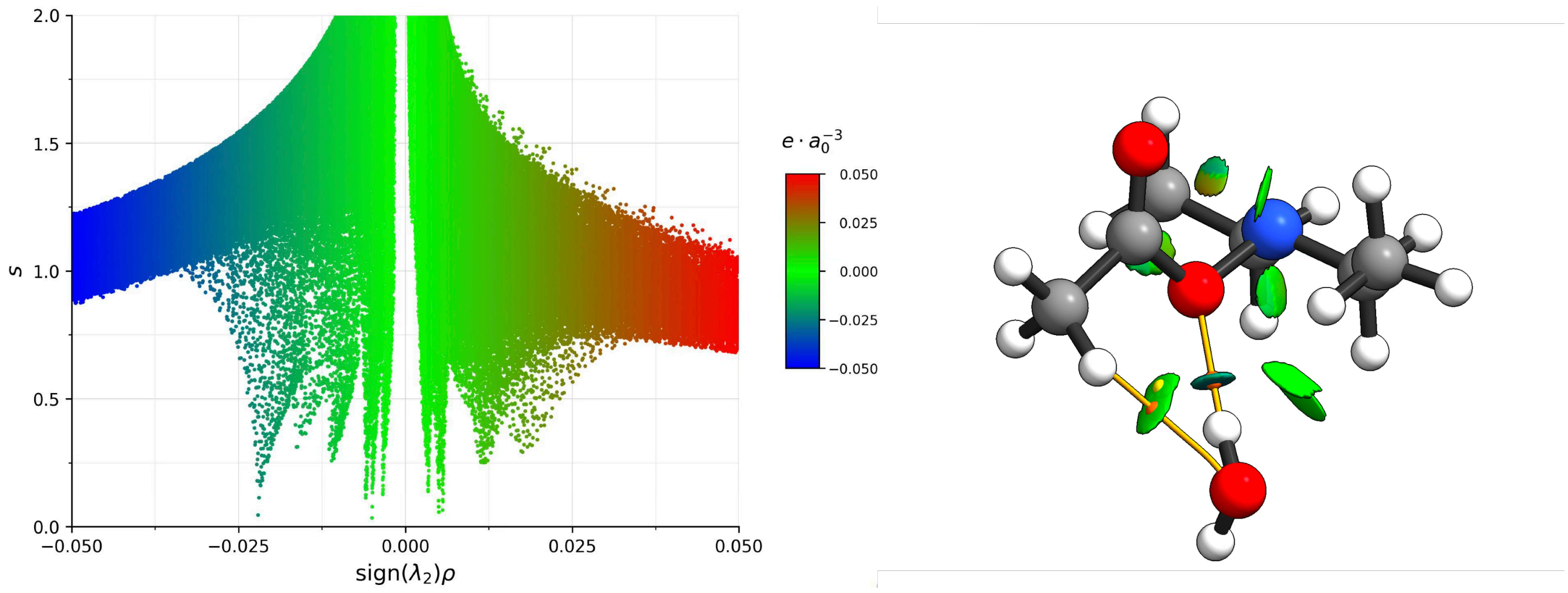


Figure S-IV: Electron density topological analysis of #1:1 (ester) water complex of #1-DEAcA at the MP2/aug-cc-pVTZ level of calculation.

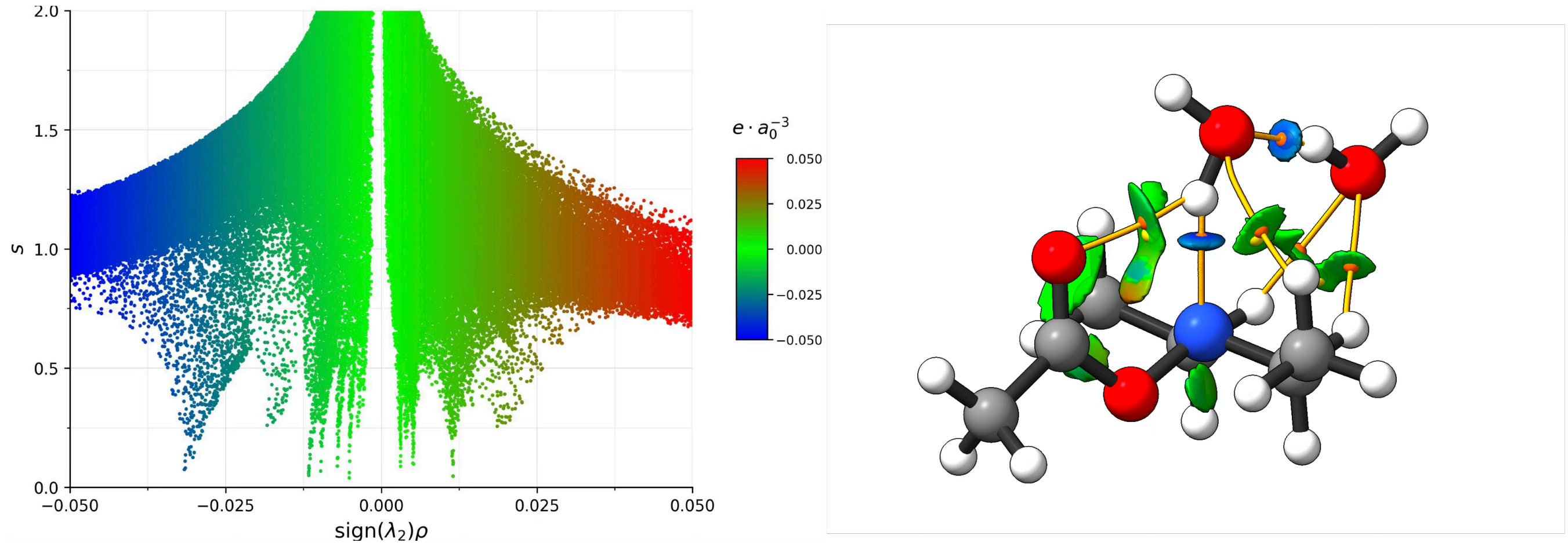


Figure S-V: Electron density topological analysis of #1:2 water complex of #1-DEAcA at the MP2/aug-cc-pVTZ level of calculation.

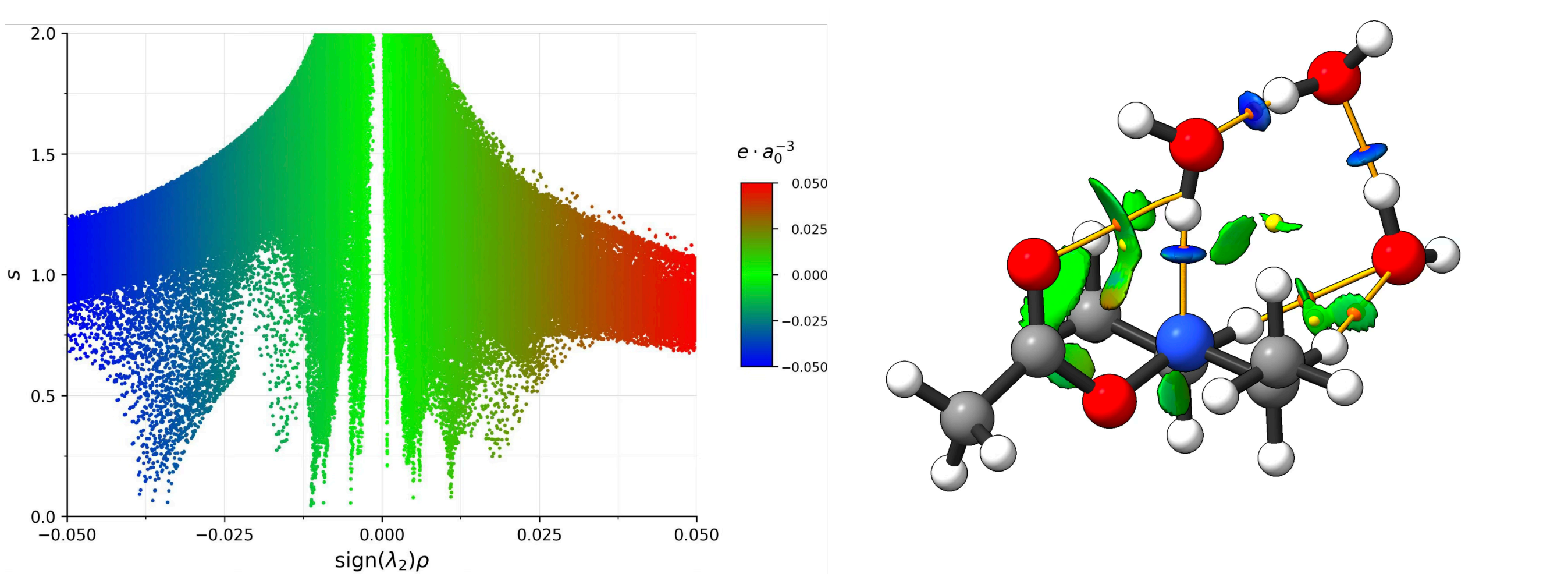


Figure S-VI: Electron density topological analysis of #1:3 water complex of #1-DEAcA at the MP2/aug-cc-pVTZ level of calculation.

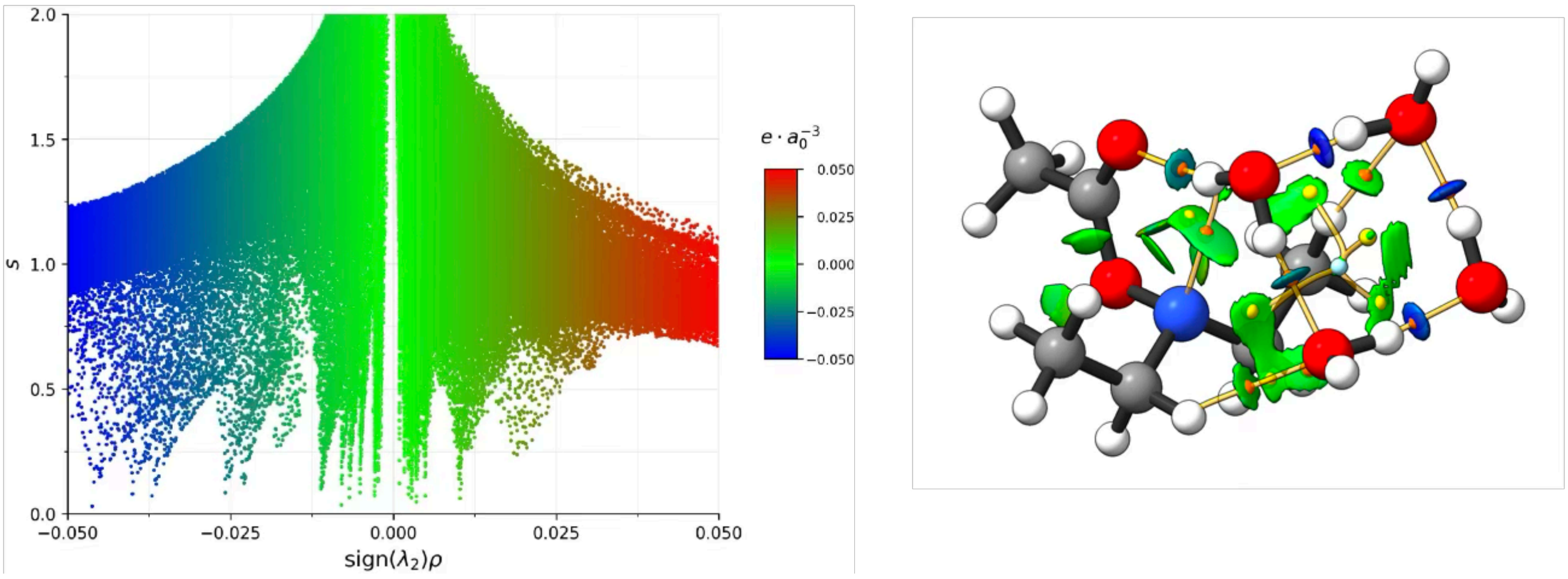


Figure S-VII: Electron density topological analysis of #1:4 water complex of #1-DEAcA at the MP2/aug-cc-pVTZ level of calculation.

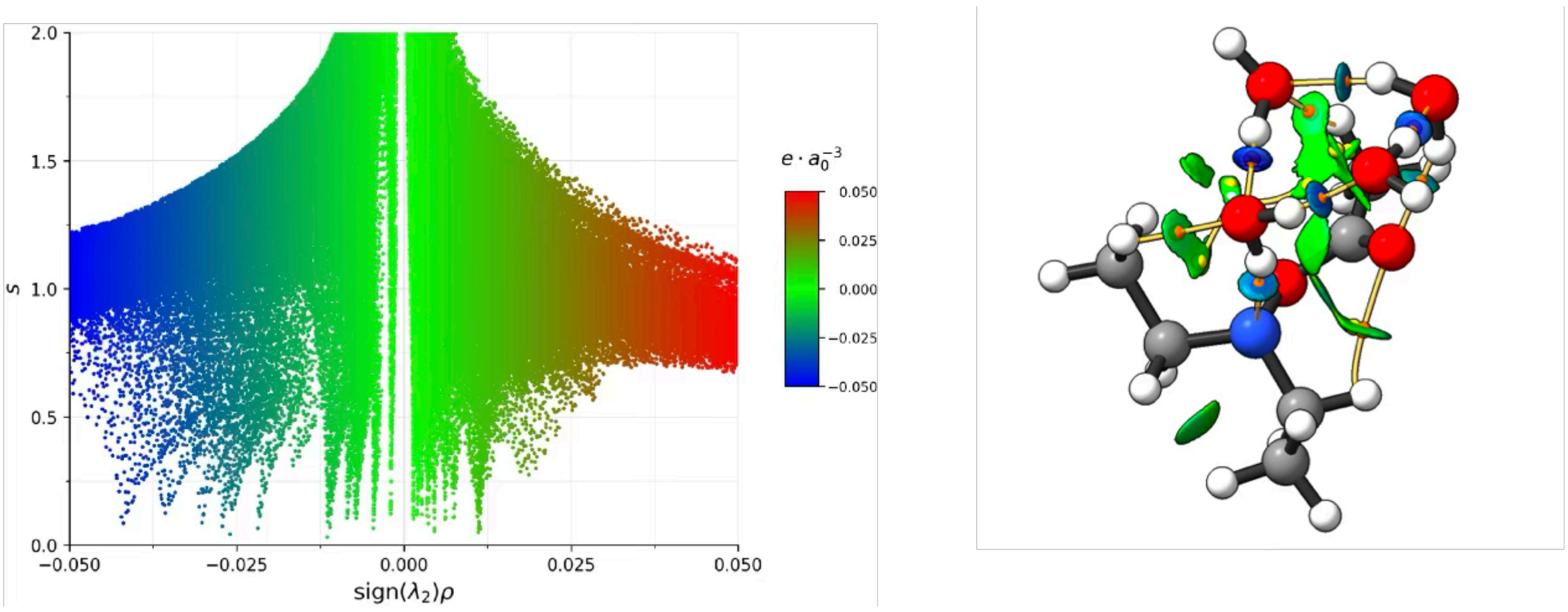


Figure S-VIII: Electron density topological analysis of #2:4 water complex of #2-DEAcA at the MP2/aug-cc-pVTZ level of calculation.

## 12 Conformational potential energy profiles for DEAcA.

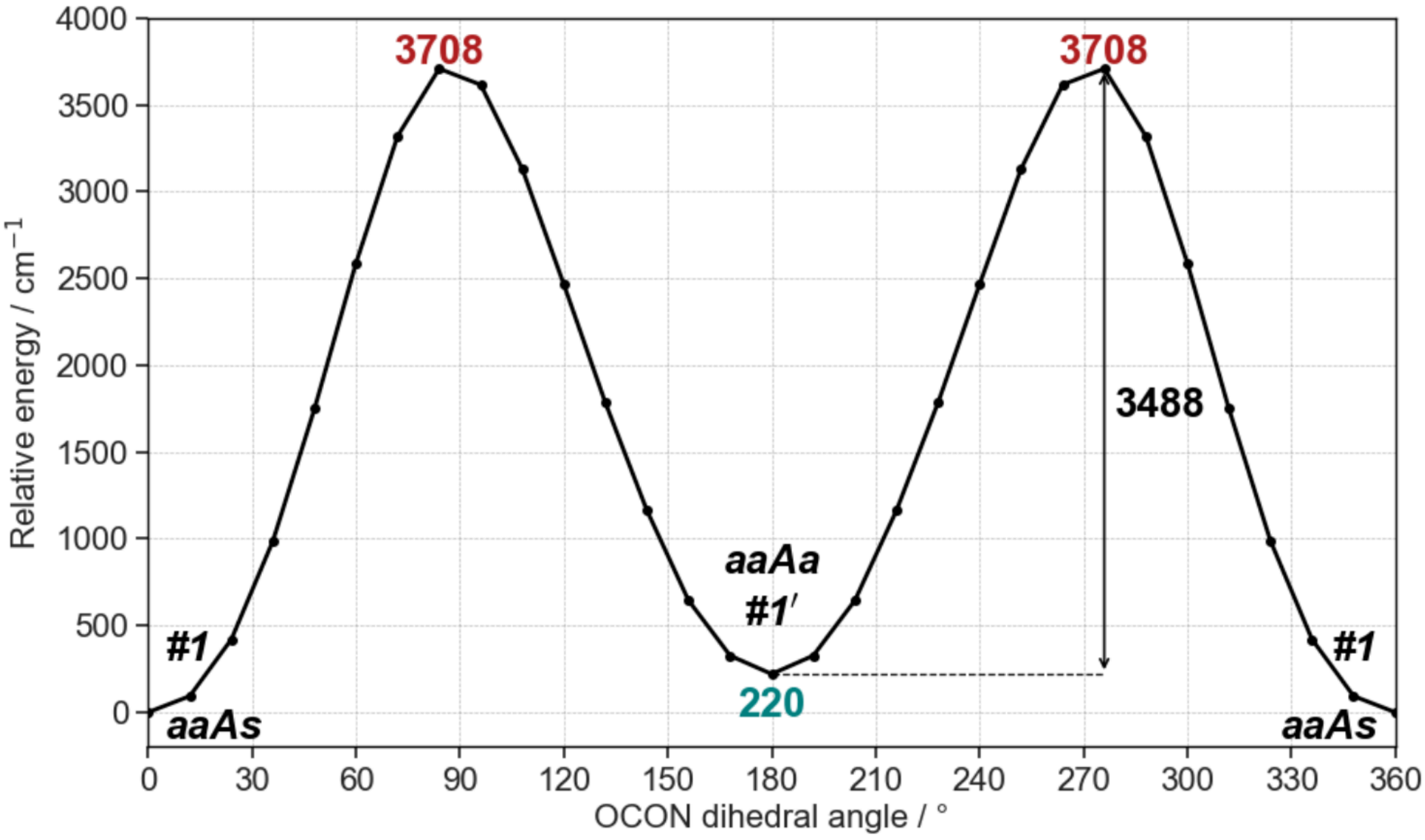


Figure S-IX: Relaxed potential energy scan for the acetyl torsion (O1N1-O2C5, step 12°) in #1-DEAcA at the B3LYP-D3(BJ)/def2-TZVP level of calculation.

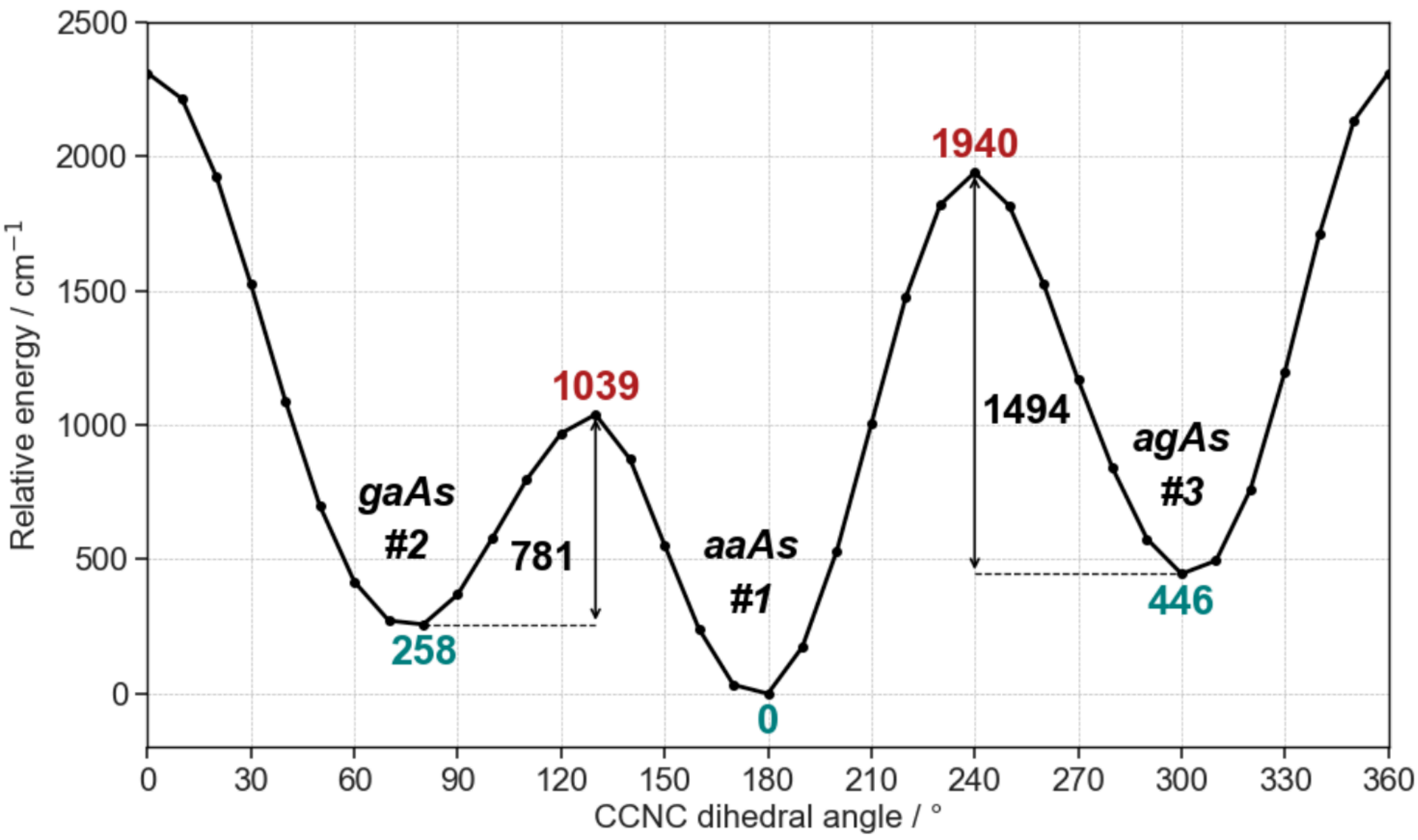


Figure S-X: Relaxed potential energy scan for the ethyl torsion (C1C2-N1C3, step 10°) in #1-DEAcA at the B3LYP-D3(BJ)/def2-TZVP level of calculation.

## 13 Conformers of DEAcA water complexes from DFT search

This section lists the water complexes of DEAcA ($n$=0-4) identified by a conformational search with CREST (using an energy threshold of 25 kJ·mol$^{-1}$) followed by B3LYP-D3(BJ)/def2-TZVP optimization. Every table reports the identification label for the involved DEAcA conformer, the relative electronic energy $E_e$, the binding energy $BE$, the rotational constants $A$, $B$, and $C$, and the electric dipole-moment components $\mu_a$, $\mu_b$, and $\mu_c$. DEAcA conformers are described according to the four-letter code introduced in Section 2.1.

Table S28: Theoretical (B3LYP-D3(BJ)/def2-TZVP) energy and spectroscopic parameters of the mono-hydrated species of DEAcA.

| Conformer | $E_e$ / kJ·mol$^{-1}$ | $BE$ / kJ·mol$^{-1}$ | $A$ / MHz | $B$ / MHz | $C$ / MHz | $\mu_a$ / D | $\mu_b$ / D | $\mu_c$ / D |
|---|---|---|---|---|---|---|---|---|
| aaAs:1w-1 | 0.00 | -45.69 | 1203.52 | 986.14 | 889.04 | 2.20 | 0.03 | 3.31 |
| aaAs:1w-2 | 13.23 | -32.46 | 1548.89 | 724.34 | 595.42 | -3.37 | 0.69 | -0.82 |
| aaAs:1w-3 | 21.15 | -24.54 | 1148.66 | 1008.09 | 802.01 | 0.96 | 2.10 | -0.84 |
| agAs:1w-1 | 15.80 | -37.93 | 1125.13 | 1001.45 | 775.53 | 3.06 | 4.08 | 1.82 |
| agAs:1w-2 | 7.35 | -43.69 | 1153.56 | 982.34 | 818.01 | -0.90 | -3.60 | -1.80 |
| agAs:1w-3 | 18.49 | -32.54 | 1725.56 | 643.78 | 563.89 | -3.53 | -0.37 | -0.64 |
| agAs:1w-4 | 25.80 | -25.23 | 1122.66 | 929.74 | 863.73 | -2.40 | 0.74 | 0.62 |
| gaAs:1w-1 | 3.70 | -48.58 | 1342.35 | 860.67 | 831.62 | -1.56 | -1.85 | -2.99 |
| gaAs:1w-2 | 3.74 | -48.53 | 1341.47 | 857.66 | 832.02 | -1.61 | -1.80 | -3.05 |
| gaAs:1w-3 | 16.41 | -32.39 | 1676.66 | 640.23 | 544.05 | -3.38 | -0.86 | -0.51 |
| gaAs:1w-4 | 24.98 | -23.82 | 1170.79 | 855.64 | 778.65 | -0.57 | 2.07 | -1.53 |
| agAs:1w-5 | 32.01 | -19.02 | 1167.29 | 1109.55 | 681.87 | -1.93 | -2.10 | 0.81 |
| ggAs':1w-1 | 21.70 | -32.60 | 2081.64 | 596.93 | 505.45 | 3.58 | 0.93 | -0.10 |
| ggAs':1w-2 | 31.76 | -22.54 | 1360.80 | 967.34 | 614.90 | -2.29 | -2.09 | 1.03 |
| ggAs':1w-3 | 42.73 | -11.57 | 2278.96 | 592.32 | 508.32 | -3.37 | -2.74 | 2.09 |
| ggAs":1w-1 | 10.11 | -44.31 | 1387.72 | 911.37 | 709.62 | -1.00 | -3.83 | -0.94 |
| ggAs":1w-2 | 22.15 | -32.26 | 2156.77 | 557.51 | 539.50 | -3.43 | 0.74 | -0.87 |
| ggAs":1w-3 | 29.39 | -25.02 | 1336.86 | 864.66 | 714.06 | 1.46 | -1.33 | -1.87 |
| ggAs":1w-4 | 42.52 | -11.90 | 2092.50 | 590.17 | 533.23 | -1.41 | -1.44 | 1.83 |
| gg'As":1w-1 | 15.50 | -44.54 | 1526.94 | 816.36 | 754.88 | -1.45 | -3.58 | 0.82 |

Table S29: Theoretical (B3LYP-D3(BJ)/def2-TZVP) energy and spectroscopic parameters of the di-hydrated species of DEAcA.

| Conformer | $E_e$ / kJ·mol$^{-1}$ | $BE$ / kJ·mol$^{-1}$ | $A$ / MHz | $B$ / MHz | $C$ / MHz | $\mu_a$ / D | $\mu_b$ / D | $\mu_c$ / D |
|---|---|---|---|---|---|---|---|---|
| aaAs:2w-1 | 0.00 | -94.06 | 1190.33 | 662.89 | 616.20 | 1.95 | 0.50 | -1.26 |
| | | | | | | | | |
| agAs:2w-1 | 7.84 | -94.27 | 1111.75 | 680.58 | 575.12 | 2.00 | -1.62 | 0.13 |
| agAs:2w-2 | 13.91 | -88.20 | 852.82 | 787.41 | 689.34 | 3.83 | -3.46 | 0.02 |
| agAs:2w-3 | 19.36 | -82.75 | 958.77 | 760.50 | 561.15 | 3.11 | -3.83 | -0.78 |
| agAs:2w-4 | 22.50 | -79.61 | 1410.32 | 542.60 | 490.39 | 1.66 | 0.53 | -0.42 |
| agAs:2w-5 | 6.33 | -93.08 | 1103.16 | 666.41 | 594.82 | 1.81 | -1.27 | 0.98 |
| agAs:2w-6 | 9.04 | -90.37 | 1083.35 | 783.17 | 632.15 | 2.82 | 0.42 | -1.57 |
| agAs:2w-7 | 17.22 | -82.18 | 823.95 | 789.31 | 635.62 | 4.16 | 2.09 | -1.36 |
| agAs:2w-8 | 18.87 | -80.53 | 930.95 | 692.28 | 567.45 | 3.68 | -4.01 | 0.69 |
| agAs:2w-9 | 21.22 | -78.18 | 1283.84 | 614.06 | 512.33 | -0.76 | -1.00 | 0.48 |
| agAs:2w-10 | 23.49 | -75.91 | 1368.56 | 463.73 | 417.23 | -2.67 | -0.56 | 0.35 |
| agAs:2w-11 | 34.35 | -65.05 | 953.72 | 711.61 | 629.91 | 0.73 | 2.84 | 1.61 |
| agAs:2w-12 | 36.81 | -62.59 | 1037.38 | 649.43 | 590.95 | 0.50 | -1.88 | -0.01 |
| | | | | | | | | |
| gaAs:2w-1 | 12.02 | -85.15 | 960.18 | 792.42 | 702.84 | 1.59 | -0.21 | -1.52 |
| | | | | | | | | |
| ggAs':2w-1 | 10.32 | -92.36 | 1071.78 | 691.62 | 683.45 | 1.45 | 1.19 | -1.18 |
| ggAs':2w-2 | 13.57 | -89.10 | 1153.77 | 644.88 | 533.92 | 2.43 | -1.94 | -0.33 |
| ggAs':2w-3 | 21.71 | -80.97 | 980.60 | 705.96 | 504.20 | 2.72 | -4.03 | -2.41 |
| ggAs':2w-4 | 22.19 | -80.48 | 1211.80 | 589.47 | 549.40 | 2.33 | -2.14 | -2.55 |
| ggAs':2w-5 | 26.37 | -76.31 | 1321.41 | 565.22 | 485.67 | 1.16 | 0.24 | -0.77 |
| ggAs':2w-6 | 27.17 | -75.50 | 1512.27 | 430.94 | 387.17 | 2.87 | -0.30 | 0.19 |
| | | | | | | | | |
| ggAs":2w-1 | 9.80 | -92.99 | 1060.31 | 803.46 | 605.42 | 1.49 | -0.96 | -1.15 |
| ggAs":2w-2 | 11.58 | -91.21 | 1131.47 | 652.01 | 545.73 | 2.25 | -2.39 | 1.09 |
| ggAs":2w-3 | 20.44 | -82.35 | 888.87 | 777.94 | 574.12 | -1.56 | -4.39 | 1.07 |
| ggAs":2w-4 | 24.43 | -78.36 | 1391.07 | 559.16 | 503.15 | -0.97 | -0.79 | 0.55 |
| ggAs":2w-5 | 27.01 | -75.78 | 1565.02 | 400.80 | 389.38 | -2.87 | -0.81 | 0.24 |
| ggAs":2w-6 | 37.83 | -64.96 | 1052.98 | 674.69 | 553.24 | 1.20 | -2.75 | -1.75 |

Table S30: Theoretical (B3LYP-D3(BJ)/def2-TZVP) energy and spectroscopic parameters of the tri-hydrated species of DEAcA.

| Conformer | $E_e$ / kJ·mol$^{-1}$ | $BE$ / kJ·mol$^{-1}$ | $A$ / MHz | $B$ / MHz | $C$ / MHz | $\mu_a$ / D | $\mu_b$ / D | $\mu_c$ / D |
|---|---|---|---|---|---|---|---|---|
| aaAs:3w-1 | 0.00 | -140.94 | 1006.84 | 459.16 | 445.71 | 1.93 | 1.19 | 0.50 |
| aaAs:3w-2 | 1.64 | -139.30 | 989.72 | 524.51 | 467.75 | -3.32 | 1.51 | -0.07 |
| aaAs:3w-3 | 2.20 | -138.74 | 793.42 | 550.01 | 502.67 | -4.68 | -1.13 | -2.21 |
| aaAs:3w-4 | 2.28 | -138.66 | 796.84 | 549.38 | 499.71 | 4.72 | 1.27 | -2.04 |
| aaAs:3w-5 | 3.56 | -137.38 | 925.89 | 527.57 | 483.42 | -0.52 | -1.15 | -1.44 |
| aaAs:3w-6 | 3.89 | -137.06 | 784.77 | 609.05 | 487.56 | 3.34 | 1.35 | -0.18 |
| aaAs:3w-7 | 4.57 | -136.37 | 993.85 | 515.07 | 461.10 | -4.94 | 1.74 | -1.47 |
| aaAs:3w-8 | 5.93 | -135.01 | 817.46 | 612.31 | 520.86 | -3.68 | 0.76 | -0.16 |
| aaAs:3w-9 | 6.69 | -134.26 | 919.29 | 521.69 | 478.48 | 2.21 | 0.76 | 3.18 |
| aaAs:3w-10 | 8.33 | -132.62 | 769.38 | 568.22 | 546.47 | -7.27 | -2.12 | 0.50 |
| aaAs:3w-11 | 10.99 | -129.95 | 783.65 | 619.52 | 497.79 | -0.98 | -3.91 | -0.35 |
| aaAs:3w-12 | 16.72 | -124.22 | 943.09 | 458.24 | 436.59 | 0.84 | -1.25 | -1.20 |
| aaAs:3w-13 | 17.11 | -123.84 | 945.18 | 459.44 | 432.93 | 0.78 | -1.14 | -0.90 |
| aaAs:3w-14 | 20.55 | -120.38 | 1065.40 | 441.85 | 360.17 | 2.00 | -2.55 | 0.02 |
| aaAs:3w-15 | 23.23 | -117.71 | 1133.84 | 367.44 | 325.40 | 2.88 | -2.19 | -1.68 |
| gaAa:3w-1 | 9.83 | -134.22 | 767.06 | 644.67 | 518.81 | -1.53 | 0.51 | -2.15 |
| gaAa:3w-2 | 10.40 | -133.66 | 1007.10 | 470.97 | 450.55 | -3.79 | 0.59 | -0.58 |
| gaAs:3w-1 | 1.18 | -142.87 | 880.82 | 547.55 | 540.20 | -3.45 | 0.28 | -3.52 |
| gaAs:3w-2 | 4.71 | -139.34 | 1051.80 | 452.62 | 424.09 | 2.38 | 1.12 | 0.17 |
| gaAs:3w-3 | 4.98 | -139.07 | 867.87 | 602.33 | 496.68 | -3.47 | -0.94 | -3.23 |
| gaAs:3w-4 | 5.05 | -139.00 | 794.00 | 641.52 | 545.33 | -2.20 | -2.03 | -1.26 |
| gaAs:3w-5 | 5.36 | -138.70 | 902.97 | 557.36 | 521.18 | -2.79 | -2.09 | -0.50 |
| gaAs:3w-6 | 5.72 | -138.33 | 827.95 | 576.14 | 500.14 | 1.76 | 0.09 | 1.79 |
| gaAs:3w-7 | 5.84 | -138.22 | 1028.56 | 460.22 | 429.90 | 2.86 | 0.75 | 0.82 |
| gaAs:3w-8 | 6.10 | -137.96 | 754.11 | 708.33 | 498.64 | 2.39 | -1.26 | -0.08 |
| gaAs:3w-9 | 6.53 | -137.52 | 757.75 | 677.23 | 522.41 | -1.08 | 0.65 | -2.10 |
| gaAs:3w-10 | 6.63 | -137.43 | 739.01 | 662.67 | 577.49 | 0.87 | 1.71 | 2.15 |
| gaAs:3w-11 | 7.27 | -136.79 | 873.56 | 553.26 | 549.67 | 2.10 | 2.02 | -3.24 |
| gaAs:3w-12 | 7.66 | -136.39 | 739.44 | 589.45 | 501.69 | -3.37 | -2.14 | -2.26 |
| gaAs:3w-13 | 9.24 | -134.82 | 848.78 | 581.07 | 469.63 | -3.85 | -1.21 | -0.40 |
| gaAs:3w-14 | 10.14 | -133.91 | 787.22 | 602.52 | 537.73 | -1.58 | -2.44 | -0.04 |
| gaAs:3w-15 | 10.85 | -133.20 | 780.57 | 592.01 | 449.17 | -3.41 | -2.52 | 1.49 |
| gaAs:3w-16 | 12.85 | -131.20 | 865.71 | 535.11 | 436.67 | -2.52 | -1.63 | -2.55 |
| gaAs:3w-17 | 13.66 | -130.39 | 960.51 | 478.81 | 454.36 | 0.64 | 1.63 | 0.74 |
| gaAs:3w-18 | 14.49 | -129.56 | 893.35 | 603.91 | 510.72 | 0.53 | -0.55 | -1.70 |
| gaAs:3w-19 | 15.52 | -128.54 | 884.45 | 519.65 | 454.04 | 3.30 | 3.78 | -0.14 |
| gaAs:3w-20 | 17.27 | -126.78 | 807.20 | 556.20 | 455.90 | -3.46 | 1.52 | -3.97 |
| gaAs:3w-21 | 20.03 | -124.03 | 901.35 | 482.44 | 417.18 | 0.83 | 0.85 | 1.35 |
| gaAs:3w-22 | 20.17 | -123.88 | 1037.61 | 415.80 | 368.66 | -0.88 | -0.05 | -0.67 |
| gaAs:3w-23 | 20.98 | -123.07 | 1002.53 | 435.80 | 370.81 | -1.46 | -0.26 | 0.51 |
| gaAs:3w-24 | 21.19 | -122.87 | 1268.08 | 317.56 | 286.49 | 0.83 | -0.98 | 1.81 |
| gaAs:3w-25 | 21.63 | -122.43 | 1135.90 | 382.32 | 348.08 | -1.50 | 0.12 | -0.22 |
| gaAs:3w-26 | 21.87 | -122.19 | 1007.08 | 425.19 | 370.19 | 0.99 | -0.48 | -1.19 |
| gaAs:3w-27 | 22.19 | -121.86 | 1048.57 | 407.11 | 369.64 | -2.28 | 0.34 | 0.59 |
| gaAs:3w-28 | 23.29 | -120.76 | 1377.11 | 360.30 | 335.48 | 0.74 | 0.98 | -2.56 |
| gaAs:3w-29 | 23.63 | -120.43 | 908.65 | 464.63 | 413.05 | 0.27 | -0.88 | 2.11 |
| gaAs:3w-30 | 27.02 | -117.03 | 1212.18 | 330.78 | 298.73 | -3.07 | -3.22 | -0.32 |
| gaAs:3w-31 | 27.51 | -116.54 | 882.83 | 471.90 | 461.76 | 0.79 | -0.11 | -2.12 |
| gaAs:3w-32 | 33.81 | -110.25 | 1028.08 | 364.85 | 316.01 | 0.92 | 0.88 | 0.10 |
| gaAs:3w-33 | 11.07 | -132.99 | 877.82 | 534.45 | 442.78 | 0.90 | 1.65 | 0.69 |
| gaSs:3w-1 | 10.96 | -143.93 | 872.53 | 538.52 | 443.88 | 0.82 | 1.56 | 0.82 |
| gaSs:3w-2 | 14.43 | -140.47 | 972.65 | 469.40 | 446.32 | 1.97 | 3.15 | 0.80 |

Table S30: Theoretical (B3LYP-D3(BJ)/def2-TZVP) energy and spectroscopic parameters of the tri-hydrated species of DEAcA. *(continued)*

| Conformer | $E_e$ / kJ·mol$^{-1}$ | $BE$ / kJ·mol$^{-1}$ | $A$ / MHz | $B$ / MHz | $C$ / MHz | $\mu_a$ / D | $\mu_b$ / D | $\mu_c$ / D |
|---|---|---|---|---|---|---|---|---|
| gg'As':3w-1 | 17.67 | -136.41 | 727.22 | 632.95 | 436.49 | -4.18 | -0.09 | 1.30 |
| gg'As':3w-2 | 28.51 | -125.57 | 1128.36 | 398.54 | 375.45 | -0.69 | -0.33 | -0.76 |
| gg'As':3w-3 | 29.12 | -124.96 | 1088.48 | 420.44 | 379.07 | -1.52 | 0.21 | 0.39 |
| gg'As':3w-4 | 29.94 | -124.14 | 1094.67 | 442.81 | 376.40 | -0.12 | 0.08 | -3.07 |
| gg'As':3w-5 | 30.90 | -123.17 | 1182.98 | 396.26 | 383.13 | -0.10 | -0.23 | -1.02 |
| gg'As':3w-6 | 32.30 | -121.78 | 1248.29 | 399.06 | 363.48 | -0.33 | -2.49 | -0.96 |
| gg'As':3w-7 | 33.59 | -120.48 | 1052.28 | 460.62 | 382.40 | -1.69 | 1.57 | 1.44 |
| gg'As':3w-8 | 36.95 | -117.13 | 1411.26 | 311.70 | 291.10 | -2.99 | 0.47 | 2.49 |
| gg'As":3w-1 | 20.36 | -134.94 | 785.15 | 630.09 | 506.54 | -1.54 | -1.04 | -1.95 |
| gg'As":3w-2 | 33.21 | -122.10 | 1065.86 | 402.02 | 361.51 | -1.57 | -0.10 | 0.43 |
| gg'As":3w-3 | 38.27 | -117.03 | 1351.70 | 303.21 | 288.24 | 3.15 | -3.19 | 0.34 |

Table S31: Theoretical (B3LYP-D3(BJ)/def2-TZVP) energy and spectroscopic parameters of the tetra-hydrated species of DEAcA.

| Conformer | $E_e$ / kJ·mol$^{-1}$ | $BE$ / kJ·mol$^{-1}$ | $A$ / MHz | $B$ / MHz | $C$ / MHz | $\mu_a$ / D | $\mu_b$ / D | $\mu_c$ / D |
|---|---|---|---|---|---|---|---|---|
| aaAs:4w-1 | 0.12 | -197.68 | 790.27 | 416.45 | 373.17 | 1.29 | -0.80 | 2.22 |
| aaAs:4w-2 | 1.66 | -196.14 | 782.05 | 425.11 | 383.83 | 0.70 | -0.60 | 2.57 |
| aaAs:4w-3 | 3.45 | -194.36 | 790.66 | 414.45 | 371.44 | 1.21 | -0.97 | -2.37 |
| aaAs:4w-4 | 7.07 | -190.74 | 818.24 | 414.33 | 377.11 | -1.83 | 2.13 | 0.73 |
| aaAs:4w-5 | 9.45 | -188.34 | 752.47 | 466.84 | 426.71 | -4.65 | 2.25 | 2.45 |
| aaAs:4w-6 | 11.59 | -186.21 | 669.33 | 483.32 | 464.13 | -3.08 | -0.21 | 2.62 |
| aaAs:4w-7 | 12.08 | -185.72 | 683.09 | 472.70 | 396.65 | 3.65 | 0.11 | 0.97 |
| aaAs:4w-8 | 12.51 | -185.29 | 887.94 | 364.76 | 350.20 | -1.04 | 0.32 | 3.20 |
| aaAs:4w-9 | 12.55 | -185.25 | 737.24 | 446.09 | 397.28 | 3.15 | -1.44 | 1.09 |
| aaAs:4w-10 | 14.31 | -183.49 | 763.71 | 413.23 | 384.96 | -3.41 | 1.26 | -0.20 |
| aaAs:4w-11 | 14.92 | -182.89 | 669.69 | 441.37 | 355.81 | 3.77 | -1.66 | 0.12 |
| aaAs:4w-12 | 19.01 | -178.79 | 774.55 | 406.14 | 348.26 | -1.49 | 1.23 | 1.37 |
| aaAs:4w-13 | 19.82 | -177.98 | 775.34 | 405.09 | 346.87 | -1.54 | 1.09 | 1.35 |
| aaAs:4w-14 | 22.00 | -175.80 | 639.19 | 542.48 | 404.04 | 2.38 | -0.75 | 0.56 |
| aaAs:4w-15 | 27.49 | -170.31 | 788.77 | 357.22 | 319.85 | -0.71 | -1.50 | 0.97 |
| aaAs:4w-16 | 31.19 | -166.61 | 834.98 | 338.69 | 297.24 | 1.92 | -1.96 | -0.36 |
| agAs:4w-1 | 2.27 | -200.87 | 772.73 | 419.19 | 399.02 | 3.56 | 0.02 | -1.67 |
| agAs:4w-2 | 2.84 | -200.31 | 783.27 | 416.18 | 394.18 | 4.83 | -0.93 | -0.21 |
| agAs:4w-3 | 4.39 | -198.75 | 762.01 | 413.97 | 392.06 | 3.66 | -0.19 | -1.72 |
| agAs:4w-4 | 4.73 | -198.42 | 687.61 | 449.08 | 390.14 | 1.85 | -1.90 | -2.46 |
| agAs:4w-5 | 4.77 | -198.38 | 668.73 | 459.12 | 395.34 | 2.34 | -2.37 | -2.36 |
| agAs:4w-6 | 5.14 | -198.01 | 775.87 | 409.58 | 386.15 | 4.96 | -0.72 | -0.13 |
| agAs:4w-7 | 5.89 | -197.25 | 862.13 | 410.95 | 376.96 | 3.21 | 1.74 | -3.01 |
| agAs:4w-8 | 6.63 | -196.51 | 724.59 | 463.02 | 407.36 | -2.19 | -1.74 | 3.10 |
| agAs:4w-9 | 7.35 | -195.80 | 667.02 | 450.42 | 385.02 | 4.46 | -2.82 | -2.98 |
| agAs:4w-10 | 7.42 | -195.73 | 664.86 | 451.11 | 385.50 | -4.44 | 2.72 | -3.19 |
| agAs:4w-11 | 7.46 | -195.68 | 775.80 | 409.15 | 397.86 | 4.39 | -1.02 | -0.10 |
| agAs:4w-12 | 7.79 | -195.36 | 672.95 | 419.28 | 390.68 | 4.01 | 0.21 | 0.25 |
| agAs:4w-13 | 8.10 | -195.05 | 753.18 | 418.37 | 348.16 | 1.73 | -1.58 | 1.62 |
| agAs:4w-14 | 8.12 | -195.03 | 747.79 | 421.04 | 349.23 | 1.66 | -1.49 | 1.76 |
| agAs:4w-15 | 8.52 | -194.62 | 844.65 | 401.27 | 379.07 | -3.67 | -0.48 | 3.84 |
| agAs:4w-16 | 8.77 | -194.37 | 748.33 | 402.24 | 372.29 | 1.11 | -1.63 | -2.11 |
| agAs:4w-17 | 8.80 | -194.35 | 710.53 | 407.37 | 376.08 | 4.06 | 0.11 | -0.66 |
| agAs:4w-18 | 9.57 | -193.58 | 766.00 | 408.25 | 392.04 | -4.50 | 0.89 | -0.04 |
| agAs:4w-19 | 9.64 | -193.50 | 710.93 | 406.73 | 376.47 | -5.51 | 1.05 | 0.20 |
| agAs:4w-20 | 9.67 | -193.48 | 775.80 | 444.52 | 381.58 | 3.83 | -0.34 | -4.27 |
| agAs:4w-21 | 9.99 | -193.15 | 722.67 | 411.96 | 392.47 | 4.69 | -1.13 | -0.06 |
| agAs:4w-22 | 10.06 | -193.09 | 720.51 | 414.13 | 395.03 | 3.81 | -0.18 | 1.53 |
| agAs:4w-23 | 10.19 | -192.95 | 744.10 | 427.77 | 353.82 | 1.14 | -1.58 | 2.00 |
| agAs:4w-24 | 10.69 | -192.45 | 622.99 | 529.66 | 446.58 | 2.30 | -0.25 | 0.45 |
| agAs:4w-25 | 10.89 | -192.26 | 734.95 | 410.23 | 383.83 | -0.79 | -1.32 | -1.22 |
| agAs:4w-26 | 11.01 | -192.14 | 718.76 | 393.86 | 343.90 | 4.56 | -0.37 | -3.53 |
| agAs:4w-27 | 11.20 | -191.94 | 747.37 | 400.40 | 380.05 | 0.96 | -1.99 | -2.00 |
| agAs:4w-28 | 11.24 | -191.91 | 751.89 | 414.63 | 348.62 | 1.75 | -1.76 | 1.66 |
| agAs:4w-29 | 11.26 | -191.89 | 737.42 | 430.86 | 365.56 | -0.83 | -1.42 | 1.23 |
| agAs:4w-30 | 11.56 | -191.59 | 749.82 | 419.38 | 345.55 | 3.34 | 1.42 | -2.93 |
| agAs:4w-31 | 11.68 | -191.47 | 807.38 | 445.64 | 398.03 | 5.12 | -1.89 | -1.86 |
| agAs:4w-32 | 12.12 | -191.02 | 730.12 | 411.91 | 401.76 | -1.05 | -2.16 | 0.17 |
| agAs:4w-33 | 12.49 | -190.65 | 742.09 | 424.45 | 355.55 | 1.24 | -2.03 | 2.03 |
| agAs:4w-34 | 12.87 | -190.27 | 757.58 | 380.29 | 363.25 | -5.95 | 1.46 | 1.28 |
| agAs:4w-35 | 12.92 | -190.23 | 734.57 | 456.38 | 441.03 | -4.56 | 2.00 | 2.18 |
| agAs:4w-36 | 13.30 | -189.84 | 787.37 | 406.75 | 356.81 | 3.35 | 1.76 | 2.69 |
| agAs:4w-37 | 13.33 | -189.82 | 756.22 | 381.57 | 364.92 | -5.91 | 1.85 | 0.52 |
| agAs:4w-38 | 13.42 | -189.73 | 623.63 | 519.21 | 439.43 | 3.39 | -0.88 | 1.59 |

Table S31: Theoretical (B3LYP-D3(BJ)/def2-TZVP) energy and spectroscopic parameters of the tetra-hydrated species of DEAcA. *(continued)*

| Conformer | $E_e$ / kJ·mol$^{-1}$ | $BE$ / kJ·mol$^{-1}$ | $A$ / MHz | $B$ / MHz | $C$ / MHz | $\mu_a$ / D | $\mu_b$ / D | $\mu_c$ / D |
|---|---|---|---|---|---|---|---|---|
| agAs:4w-39 | 13.58 | -189.58 | 796.39 | 448.00 | 395.55 | 5.09 | -2.40 | -1.68 |
| agAs:4w-40 | 13.86 | -189.28 | 782.35 | 409.42 | 364.01 | 1.94 | 1.33 | 1.83 |
| agAs:4w-41 | 14.22 | -188.92 | 677.60 | 473.10 | 454.11 | -2.26 | 0.11 | 3.05 |
| agAs:4w-42 | 14.39 | -188.75 | 787.64 | 375.11 | 356.94 | 5.97 | -1.98 | 0.06 |
| agAs:4w-43 | 14.51 | -188.64 | 714.44 | 440.71 | 439.73 | 3.02 | 2.70 | 0.85 |
| agAs:4w-44 | 14.65 | -188.50 | 686.72 | 478.84 | 388.91 | 4.08 | -0.25 | -1.13 |
| agAs:4w-45 | 15.12 | -188.03 | 707.22 | 441.29 | 437.03 | 2.44 | -0.65 | -3.12 |
| agAs:4w-46 | 15.34 | -187.81 | 764.73 | 396.16 | 375.59 | -3.67 | -0.85 | -2.81 |
| agAs:4w-47 | 15.53 | -187.61 | 791.32 | 402.16 | 350.04 | 3.92 | 1.68 | 2.19 |
| agAs:4w-48 | 15.65 | -187.50 | 808.36 | 387.71 | 356.05 | -4.41 | 2.85 | 0.07 |
| agAs:4w-49 | 15.83 | -187.32 | 664.69 | 479.84 | 442.14 | 2.45 | -1.98 | 0.96 |
| agAs:4w-50 | 16.38 | -186.76 | 721.31 | 462.91 | 441.53 | -4.61 | -1.03 | -0.68 |
| agAs:4w-51 | 16.86 | -186.29 | 797.76 | 395.64 | 335.73 | 0.95 | 1.38 | 2.42 |
| agAs:4w-52 | 17.11 | -186.04 | 755.36 | 446.04 | 414.40 | 3.59 | -2.85 | 0.21 |
| agAs:4w-53 | 17.12 | -186.03 | 780.25 | 389.09 | 370.76 | 3.76 | 1.45 | -2.78 |
| agAs:4w-54 | 17.57 | -185.58 | 725.05 | 422.34 | 347.18 | 3.34 | 0.50 | 0.58 |
| agAs:4w-55 | 17.57 | -185.58 | 677.45 | 466.09 | 445.22 | 2.14 | -3.25 | 0.77 |
| agAs:4w-56 | 17.60 | -185.55 | 654.38 | 486.77 | 422.21 | 3.14 | 0.17 | 0.98 |
| agAs:4w-57 | 17.64 | -185.50 | 634.59 | 514.21 | 455.83 | -2.65 | 1.44 | 2.43 |
| agAs:4w-58 | 17.68 | -185.46 | 795.03 | 396.86 | 333.25 | -0.90 | -1.24 | 2.49 |
| agAs:4w-59 | 17.72 | -185.43 | 856.01 | 426.48 | 399.78 | 4.32 | 0.30 | 0.90 |
| agAs:4w-60 | 18.28 | -184.87 | 595.17 | 566.47 | 459.16 | 2.25 | 0.28 | -2.83 |
| agAs:4w-61 | 18.37 | -184.78 | 656.29 | 520.73 | 414.83 | 1.68 | -1.92 | -2.40 |
| agAs:4w-62 | 18.41 | -184.74 | 730.25 | 432.46 | 387.64 | -3.58 | 1.24 | -0.58 |
| agAs:4w-63 | 18.77 | -184.37 | 835.09 | 366.80 | 347.97 | -1.01 | 3.28 | 0.39 |
| agAs:4w-64 | 18.96 | -184.18 | 774.57 | 443.25 | 414.31 | -3.73 | 1.05 | -0.20 |
| agAs:4w-65 | 19.02 | -184.13 | 799.95 | 433.69 | 361.23 | -4.41 | -1.89 | 1.52 |
| agAs:4w-66 | 19.06 | -184.09 | 653.62 | 484.04 | 441.90 | 2.59 | -2.01 | 0.91 |
| agAs:4w-67 | 19.19 | -183.95 | 757.38 | 435.64 | 419.74 | -5.82 | 0.33 | 2.83 |
| agAs:4w-68 | 19.25 | -183.90 | 773.37 | 335.16 | 287.79 | 3.17 | 0.94 | 0.77 |
| agAs:4w-69 | 19.33 | -183.82 | 741.06 | 444.32 | 425.64 | -4.59 | 0.70 | 0.50 |
| agAs:4w-70 | 19.42 | -183.73 | 624.99 | 458.42 | 343.47 | 2.45 | -2.28 | -0.91 |
| agAs:4w-71 | 19.45 | -183.69 | 730.87 | 388.70 | 332.53 | 1.50 | -0.87 | -0.13 |
| agAs:4w-72 | 19.61 | -183.54 | 720.64 | 444.71 | 370.29 | 3.96 | 0.53 | -0.39 |
| agAs:4w-73 | 19.63 | -183.52 | 657.86 | 444.69 | 433.01 | 4.58 | 0.20 | 0.18 |
| agAs:4w-74 | 19.81 | -183.34 | 783.67 | 413.75 | 334.51 | 0.30 | 0.14 | -0.79 |
| agAs:4w-75 | 19.88 | -183.27 | 647.82 | 445.17 | 350.48 | 3.60 | -1.49 | 0.80 |
| agAs:4w-76 | 19.95 | -183.19 | 730.85 | 415.20 | 367.62 | 3.17 | 0.24 | -0.76 |
| agAs:4w-77 | 20.01 | -183.13 | 785.30 | 418.17 | 357.86 | 3.13 | 3.41 | -0.28 |
| agAs:4w-78 | 20.17 | -182.98 | 646.90 | 486.44 | 394.88 | -4.54 | -0.72 | 1.64 |
| agAs:4w-79 | 20.36 | -182.78 | 1002.14 | 259.29 | 239.27 | 1.98 | -0.09 | -2.69 |
| agAs:4w-80 | 20.42 | -182.73 | 862.21 | 402.41 | 380.63 | 5.60 | -0.62 | 0.22 |
| agAs:4w-81 | 20.42 | -182.73 | 855.76 | 364.32 | 338.95 | 4.25 | -3.00 | 1.36 |
| agAs:4w-82 | 20.44 | -182.70 | 850.58 | 365.29 | 339.79 | 4.17 | -2.90 | 1.51 |
| agAs:4w-83 | 20.70 | -182.45 | 634.24 | 456.08 | 345.01 | 3.83 | -1.49 | -0.15 |
| agAs:4w-84 | 20.97 | -182.18 | 797.45 | 384.98 | 339.20 | 5.21 | -0.70 | 1.19 |
| agAs:4w-85 | 20.97 | -182.18 | 570.76 | 527.38 | 402.14 | 2.66 | -1.57 | 1.40 |
| agAs:4w-86 | 20.98 | -182.16 | 649.19 | 487.77 | 442.87 | 2.78 | -2.17 | 2.72 |
| agAs:4w-87 | 21.03 | -182.11 | 659.95 | 491.64 | 417.42 | -4.86 | 0.18 | 1.05 |
| agAs:4w-88 | 21.20 | -181.95 | 757.56 | 404.64 | 324.08 | 1.68 | -0.30 | 0.88 |
| agAs:4w-89 | 21.25 | -181.90 | 826.17 | 376.91 | 351.38 | -1.44 | -1.35 | 1.83 |
| agAs:4w-90 | 21.28 | -181.86 | 824.04 | 425.29 | 379.92 | 6.23 | -1.41 | -2.35 |
| agAs:4w-91 | 21.44 | -184.41 | 683.03 | 467.25 | 424.30 | -0.30 | -0.52 | 3.12 |
| agAs:4w-92 | 21.56 | -181.59 | 838.99 | 393.39 | 339.19 | -0.69 | 2.21 | 3.25 |
| agAs:4w-93 | 21.65 | -181.49 | 666.19 | 491.47 | 439.12 | -0.15 | -1.51 | -0.21 |

Table S31: Theoretical (B3LYP-D3(BJ)/def2-TZVP) energy and spectroscopic parameters of the tetra-hydrated species of DEAcA. *(continued)*

| Conformer | $E_e$ / kJ·mol$^{-1}$ | $BE$ / kJ·mol$^{-1}$ | $A$ / MHz | $B$ / MHz | $C$ / MHz | $\mu_a$ / D | $\mu_b$ / D | $\mu_c$ / D |
|---|---|---|---|---|---|---|---|---|
| agAs:4w-94 | 21.82 | -181.32 | 831.60 | 364.25 | 347.53 | -3.10 | 3.46 | -0.11 |
| agAs:4w-95 | 22.27 | -180.87 | 661.91 | 463.44 | 331.36 | 3.23 | -1.78 | -0.63 |
| agAs:4w-96 | 22.37 | -180.78 | 683.93 | 421.32 | 361.18 | 1.69 | 0.53 | -1.54 |
| agAs:4w-97 | 22.59 | -180.56 | 639.20 | 466.14 | 327.06 | 2.59 | -2.41 | -1.44 |
| agAs:4w-98 | 22.65 | -180.50 | 786.57 | 422.18 | 361.33 | 4.27 | -1.66 | -3.76 |
| agAs:4w-99 | 22.66 | -180.49 | 903.02 | 329.82 | 305.98 | 3.85 | -0.94 | 0.89 |
| agAs:4w-100 | 22.67 | -180.48 | 759.35 | 396.81 | 333.70 | 0.94 | -1.01 | -0.35 |
| agAs:4w-101 | 22.68 | -180.47 | 897.63 | 331.21 | 306.31 | 3.80 | -0.84 | 1.00 |
| agAs:4w-102 | 22.79 | -180.36 | 592.56 | 513.72 | 382.96 | 3.97 | -3.87 | 0.12 |
| agAs:4w-103 | 22.82 | -180.33 | 766.58 | 328.93 | 278.02 | -3.16 | -0.57 | -0.55 |
| agAs:4w-104 | 22.97 | -180.18 | 725.62 | 490.71 | 411.36 | 5.06 | 1.98 | 1.94 |
| agAs:4w-105 | 23.08 | -180.08 | 612.24 | 480.32 | 373.21 | 4.68 | 0.03 | -2.37 |
| agAs:4w-106 | 23.21 | -179.94 | 628.45 | 486.53 | 331.95 | 0.51 | -4.31 | -0.58 |
| agAs:4w-107 | 23.22 | -179.92 | 1005.85 | 260.37 | 240.12 | 0.10 | -0.29 | -1.56 |
| agAs:4w-108 | 23.36 | -179.79 | 582.86 | 470.75 | 335.08 | 0.78 | -4.30 | 0.27 |
| agAs:4w-109 | 23.47 | -179.67 | 849.13 | 391.04 | 331.45 | 5.12 | -0.02 | -0.33 |
| agAs:4w-110 | 23.66 | -179.48 | 766.23 | 398.86 | 339.56 | 1.47 | 0.53 | 0.07 |
| agAs:4w-111 | 23.79 | -187.96 | 671.98 | 487.81 | 374.58 | 5.33 | 1.03 | -1.37 |
| agAs:4w-112 | 24.03 | -179.12 | 667.02 | 480.66 | 447.49 | 2.04 | -1.28 | -0.14 |
| agAs:4w-113 | 24.18 | -178.97 | 741.47 | 402.59 | 390.66 | -2.33 | 1.66 | 0.89 |
| agAs:4w-114 | 24.33 | -178.81 | 642.17 | 530.82 | 389.06 | 4.19 | -0.78 | -1.09 |
| agAs:4w-115 | 24.49 | -178.65 | 701.64 | 484.22 | 375.35 | 0.47 | -0.12 | 0.25 |
| agAs:4w-116 | 24.54 | -178.61 | 874.79 | 260.04 | 247.49 | 2.62 | 0.88 | -1.68 |
| agAs:4w-117 | 24.98 | -178.17 | 657.14 | 448.27 | 343.51 | 4.69 | -2.16 | -1.11 |
| agAs:4w-118 | 25.13 | -180.72 | 638.98 | 494.25 | 417.37 | 0.42 | -2.79 | 0.41 |
| agAs:4w-119 | 25.32 | -177.82 | 854.90 | 388.21 | 358.10 | 5.96 | 2.09 | 1.14 |
| agAs:4w-120 | 25.56 | -177.58 | 830.84 | 347.12 | 324.07 | 3.62 | -0.28 | 0.02 |
| agAs:4w-121 | 25.58 | -177.57 | 730.20 | 466.54 | 431.34 | -2.85 | 0.72 | 2.59 |
| agAs:4w-122 | 26.37 | -176.78 | 651.82 | 451.86 | 384.75 | -2.51 | 0.11 | -0.43 |
| agAs:4w-123 | 26.81 | -176.34 | 752.92 | 404.63 | 388.28 | -1.50 | 3.57 | -0.75 |
| agAs:4w-124 | 27.14 | -176.00 | 875.27 | 374.61 | 349.67 | -4.57 | -0.56 | 0.84 |
| agAs:4w-125 | 27.49 | -178.36 | 621.94 | 524.71 | 448.10 | 1.65 | -3.91 | 2.90 |
| agAs:4w-126 | 27.74 | -175.40 | 765.13 | 391.84 | 386.90 | 3.92 | 0.58 | 0.09 |
| agAs:4w-127 | 27.86 | -175.29 | 601.34 | 537.46 | 440.00 | -0.29 | -1.49 | -0.95 |
| agAs:4w-128 | 28.99 | -174.16 | 657.05 | 488.43 | 426.16 | 2.15 | -0.53 | -0.23 |
| agAs:4w-129 | 30.24 | -172.91 | 697.77 | 445.10 | 425.51 | 3.00 | 2.35 | 3.22 |
| agAs:4w-130 | 30.25 | -172.89 | 696.04 | 440.56 | 398.86 | 6.50 | -1.86 | -2.59 |
| agAs:4w-131 | 30.55 | -172.60 | 1058.77 | 194.26 | 179.37 | -4.48 | -0.26 | -0.52 |
| agAs:4w-132 | 33.73 | -169.41 | 774.01 | 398.27 | 341.26 | 2.58 | 0.98 | -2.78 |
| agAs:4w-133 | 33.85 | -169.29 | 701.09 | 416.91 | 397.14 | -0.58 | 0.02 | -1.99 |
| agAs:4w-134 | 34.02 | -169.13 | 940.86 | 340.88 | 294.17 | 0.35 | 1.49 | 0.42 |
| agAs:4w-135 | 34.20 | -168.95 | 961.99 | 329.19 | 300.41 | -0.32 | 2.09 | -0.40 |
| agAs:4w-136 | 34.85 | -168.30 | 741.03 | 410.14 | 394.74 | -1.55 | -1.03 | -0.72 |
| agAs:4w-137 | 34.99 | -168.15 | 750.90 | 402.21 | 391.78 | -1.22 | -0.54 | -0.10 |
| agAs:4w-138 | 35.09 | -168.06 | 744.52 | 373.08 | 349.62 | 0.61 | 0.18 | 1.92 |
| agAs:4w-139 | 35.41 | -170.44 | 704.50 | 398.54 | 382.19 | 4.92 | 0.64 | -2.85 |
| agAs:4w-140 | 36.21 | -166.93 | 845.94 | 357.90 | 302.94 | -3.24 | -0.46 | 1.05 |
| agAs:4w-141 | 36.55 | -166.60 | 963.08 | 329.90 | 299.39 | 4.67 | -0.87 | -0.03 |
| agAs:4w-142 | 36.86 | -166.28 | 796.25 | 341.69 | 317.09 | 0.37 | -0.47 | -0.95 |
| agAs:4w-143 | 36.88 | -166.27 | 989.09 | 265.76 | 247.75 | 3.08 | 3.06 | 0.77 |
| agAs:4w-144 | 36.96 | -166.18 | 846.72 | 350.02 | 294.52 | -3.04 | 1.22 | 0.10 |
| agAs:4w-145 | 37.65 | -168.21 | 812.90 | 384.02 | 341.28 | 0.01 | -2.56 | 1.03 |
| agAs:4w-146 | 38.54 | -164.60 | 658.40 | 432.97 | 302.55 | 0.10 | 2.45 | -0.22 |
| agSs:4w-1 | 38.47 | -182.13 | 828.90 | 371.85 | 332.59 | 0.26 | 0.41 | -1.15 |

Table S31: Theoretical (B3LYP-D3(BJ)/def2-TZVP) energy and spectroscopic parameters of the tetra-hydrated species of DEAcA. *(continued)*

| Conformer | $E_e$ / kJ·mol$^{-1}$ | $BE$ / kJ·mol$^{-1}$ | $A$ / MHz | $B$ / MHz | $C$ / MHz | $\mu_a$ / D | $\mu_b$ / D | $\mu_c$ / D |
|---|---|---|---|---|---|---|---|---|
| agSs:4w-2 | 41.65 | -178.94 | 820.39 | 357.68 | 343.41 | -2.46 | 0.16 | -0.34 |
| agSs:4w-3 | 42.01 | -178.58 | 824.63 | 375.53 | 330.79 | 2.75 | 0.74 | -1.84 |
| agSs:4w-4 | 44.63 | -175.96 | 870.99 | 407.88 | 354.97 | 0.19 | 0.65 | -0.58 |
| gaAa:4w-1 | 10.61 | -193.79 | 836.79 | 374.45 | 337.32 | 1.40 | -1.66 | -1.48 |
| gaAs:4w-1 | 0.00 | -200.91 | 714.33 | 450.34 | 409.26 | 4.42 | -1.32 | -0.87 |
| gaAs:4w-2 | 4.73 | -196.19 | 777.22 | 435.52 | 405.18 | -1.32 | -1.19 | 2.93 |
| gaAs:4w-3 | 13.48 | -187.44 | 677.82 | 504.59 | 457.28 | 1.50 | 1.43 | -3.02 |
| gaAs:4w-4 | 14.73 | -186.18 | 643.57 | 500.84 | 410.39 | -3.20 | -0.23 | -0.91 |
| gaAs:4w-5 | 16.53 | -184.38 | 734.06 | 423.82 | 336.29 | 0.73 | 0.34 | 2.63 |
| gaAs:4w-6 | 19.57 | -181.34 | 781.58 | 364.32 | 325.17 | 2.30 | -0.26 | 0.60 |
| gaAs:4w-7 | 21.26 | -179.66 | 882.32 | 368.62 | 344.45 | -5.00 | 0.25 | 0.69 |
| gaAs:4w-8 | 21.80 | -179.12 | 787.05 | 380.93 | 306.69 | 2.18 | 0.70 | -2.36 |
| gaAs:4w-9 | 23.71 | -177.20 | 710.65 | 483.15 | 445.43 | -3.36 | -0.72 | 1.47 |
| gaAs:4w-10 | 25.19 | -175.72 | 626.61 | 453.77 | 381.02 | -1.58 | 1.46 | -1.19 |
| gaAs:4w-11 | 26.01 | -183.29 | 765.50 | 372.15 | 335.75 | -1.79 | 0.88 | 3.10 |
| gaAs:4w-12 | 31.15 | -169.77 | 772.82 | 363.42 | 320.98 | 1.75 | 1.50 | -1.31 |
| gaAs:4w-13 | 43.30 | -157.60 | 835.77 | 368.24 | 324.83 | 0.74 | 0.10 | -2.13 |
| gaAs:4w-14 | 43.52 | -157.39 | 955.72 | 329.26 | 307.99 | 0.59 | 0.78 | -2.30 |
| ggAs1:4w-1 | 2.94 | -203.47 | 757.74 | 432.17 | 409.99 | -2.15 | -0.51 | 3.48 |
| ggAs1:4w-2 | 3.27 | -203.15 | 729.98 | 448.11 | 388.78 | 3.97 | -1.15 | -0.48 |
| ggAs1:4w-3 | 4.17 | -202.24 | 750.85 | 437.44 | 412.17 | -1.43 | -0.32 | 3.46 |
| ggAs1:4w-4 | 4.79 | -201.63 | 778.46 | 423.09 | 366.25 | 3.53 | -1.29 | 1.20 |
| ggAs1:4w-5 | 5.40 | -201.02 | 787.22 | 416.41 | 365.06 | 4.67 | -1.18 | -0.62 |
| ggAs1:4w-6 | 6.95 | -199.47 | 782.09 | 413.47 | 356.74 | 4.87 | -1.06 | -0.46 |
| ggAs1:4w-7 | 7.20 | -199.22 | 848.81 | 370.20 | 337.26 | 1.66 | -1.73 | 1.36 |
| ggAs1:4w-8 | 7.48 | -198.94 | 721.32 | 421.11 | 345.56 | 1.72 | -1.07 | 1.87 |
| ggAs1:4w-9 | 11.47 | -194.94 | 620.30 | 536.96 | 422.70 | 3.28 | 0.08 | -1.80 |
| ggAs1:4w-10 | 12.93 | -193.49 | 638.16 | 503.88 | 458.04 | 2.25 | 0.51 | -3.69 |
| ggAs1:4w-11 | 13.21 | -193.21 | 702.29 | 517.98 | 426.46 | 4.38 | 0.60 | 0.53 |
| ggAs1:4w-12 | 13.33 | -193.08 | 681.73 | 443.44 | 349.06 | 5.29 | -1.51 | 0.16 |
| ggAs1:4w-13 | 14.21 | -192.20 | 733.08 | 423.15 | 371.54 | 0.80 | 0.73 | 1.85 |
| ggAs1:4w-14 | 14.75 | -191.67 | 784.43 | 381.87 | 311.36 | 1.92 | -2.01 | 0.90 |
| ggAs1:4w-15 | 14.81 | -191.60 | 794.15 | 398.51 | 327.33 | 5.78 | -1.65 | -1.45 |
| ggAs1:4w-16 | 15.61 | -190.80 | 786.83 | 423.67 | 421.33 | 3.76 | -1.36 | -0.98 |
| ggAs1:4w-17 | 16.27 | -190.15 | 739.19 | 363.23 | 314.06 | 5.04 | -2.40 | 0.60 |
| ggAs1:4w-18 | 17.26 | -189.16 | 875.76 | 358.57 | 339.28 | 1.06 | -0.27 | 2.56 |
| ggAs1:4w-19 | 17.32 | -189.09 | 711.01 | 470.02 | 423.27 | 4.30 | -1.42 | -0.32 |
| ggAs1:4w-20 | 18.43 | -187.98 | 995.94 | 326.06 | 319.30 | -4.00 | 3.30 | 0.52 |
| ggAs1:4w-21 | 18.84 | -187.57 | 696.26 | 463.56 | 411.10 | 1.85 | -2.67 | 2.12 |
| ggAs1:4w-22 | 19.57 | -186.84 | 822.19 | 392.67 | 370.79 | -4.35 | 1.66 | 2.20 |
| ggAs1:4w-23 | 20.58 | -185.84 | 771.40 | 415.07 | 359.88 | 1.77 | -1.48 | -3.35 |
| ggAs1:4w-24 | 21.65 | -184.76 | 762.17 | 435.21 | 417.54 | -2.61 | 0.84 | 2.83 |
| ggAs1:4w-25 | 22.49 | -183.92 | 676.04 | 495.73 | 433.43 | -5.39 | -0.61 | -1.67 |
| ggAs1:4w-26 | 22.71 | -183.71 | 672.88 | 484.19 | 383.51 | 3.62 | 0.56 | -0.24 |
| ggAs1:4w-27 | 23.12 | -183.29 | 845.85 | 365.93 | 344.63 | 2.40 | -2.58 | -1.20 |
| ggAs1:4w-28 | 23.75 | -182.67 | 1130.25 | 237.52 | 224.30 | 2.04 | -1.99 | -1.86 |
| ggAs1:4w-29 | 23.84 | -182.58 | 728.94 | 343.93 | 274.63 | 3.17 | 0.95 | -0.57 |
| ggAs1:4w-30 | 25.48 | -180.94 | 758.56 | 382.25 | 334.14 | -3.53 | 0.59 | -0.69 |
| ggAs1:4w-31 | 25.95 | -180.47 | 680.89 | 488.70 | 386.09 | -0.25 | 1.95 | 2.00 |
| ggAs1:4w-32 | 26.02 | -180.40 | 752.10 | 398.49 | 374.54 | 1.61 | -0.25 | -3.76 |
| ggAs1:4w-33 | 26.03 | -180.38 | 756.57 | 438.41 | 413.28 | -5.17 | -0.69 | -0.09 |
| ggAs1:4w-34 | 26.83 | -179.58 | 610.02 | 541.29 | 441.90 | -1.19 | -1.19 | 1.26 |
| ggAs1:4w-35 | 26.86 | -179.55 | 624.55 | 534.90 | 411.78 | -1.26 | 3.63 | 2.69 |

Table S31: Theoretical (B3LYP-D3(BJ)/def2-TZVP) energy and spectroscopic parameters of the tetra-hydrated species of DEAcA. *(continued)*

| Conformer | $E_e$ / kJ·mol$^{-1}$ | $BE$ / kJ·mol$^{-1}$ | $A$ / MHz | $B$ / MHz | $C$ / MHz | $\mu_a$ / D | $\mu_b$ / D | $\mu_c$ / D |
|---|---|---|---|---|---|---|---|---|
| ggAs1:4w-36 | 26.89 | -179.53 | 626.33 | 512.94 | 409.39 | -0.57 | -1.67 | -0.85 |
| ggAs1:4w-37 | 28.33 | -178.08 | 782.33 | 352.66 | 312.44 | 3.92 | -0.86 | -1.03 |
| ggAs1:4w-38 | 28.44 | -177.98 | 824.74 | 395.94 | 349.25 | 5.85 | 1.63 | -0.69 |
| ggAs1:4w-39 | 28.73 | -177.68 | 716.86 | 493.08 | 417.40 | 2.44 | -5.28 | 0.00 |
| ggAs1:4w-40 | 28.96 | -177.46 | 601.78 | 543.86 | 408.60 | 3.47 | 0.61 | 1.31 |
| ggAs1:4w-41 | 30.31 | -176.10 | 640.16 | 514.03 | 428.62 | -1.85 | -1.18 | -1.69 |
| ggAs1:4w-42 | 31.84 | -174.57 | 716.39 | 432.08 | 377.55 | 0.83 | 1.88 | 0.75 |
| | | | | | | | | |
| ggAs2:4w-1 | 1.89 | -204.64 | 766.08 | 469.09 | 381.21 | 2.15 | -0.01 | -3.49 |
| ggAs2:4w-2 | 5.55 | -200.98 | 781.20 | 420.67 | 352.25 | 4.83 | -1.06 | -0.28 |
| ggAs2:4w-3 | 7.66 | -198.87 | 634.61 | 500.10 | 379.89 | 2.34 | -2.58 | -2.19 |
| ggAs2:4w-4 | 8.36 | -198.17 | 649.39 | 487.64 | 375.31 | 1.93 | -2.06 | -2.42 |
| ggAs2:4w-5 | 9.31 | -197.22 | 861.98 | 393.25 | 354.99 | 3.33 | -3.01 | -2.72 |
| ggAs2:4w-6 | 9.88 | -196.64 | 636.04 | 500.34 | 378.00 | 4.89 | -1.18 | -0.40 |
| ggAs2:4w-7 | 9.98 | -196.55 | 727.16 | 493.66 | 394.70 | 2.43 | -1.27 | 1.50 |
| ggAs2:4w-8 | 12.19 | -194.34 | 638.33 | 476.66 | 370.53 | 4.93 | -1.05 | -0.38 |
| ggAs2:4w-9 | 13.37 | -193.15 | 629.20 | 531.54 | 432.54 | 2.03 | -1.26 | -0.07 |
| ggAs2:4w-10 | 14.83 | -191.70 | 769.56 | 449.35 | 408.63 | 4.22 | -3.38 | 0.48 |
| ggAs2:4w-11 | 14.94 | -191.59 | 782.50 | 473.04 | 386.61 | 4.58 | -2.59 | -1.53 |
| ggAs2:4w-12 | 15.70 | -190.84 | 642.67 | 529.30 | 403.83 | 2.70 | -0.04 | 0.14 |
| ggAs2:4w-13 | 16.01 | -190.53 | 675.81 | 440.65 | 368.84 | 1.08 | -1.47 | 2.41 |
| ggAs2:4w-14 | 16.17 | -190.35 | 624.48 | 524.67 | 423.90 | 3.11 | -2.15 | 0.85 |
| ggAs2:4w-15 | 16.36 | -190.16 | 747.70 | 489.62 | 392.24 | 4.44 | -3.02 | -1.50 |
| ggAs2:4w-16 | 16.45 | -190.08 | 716.20 | 469.62 | 409.90 | 2.14 | -3.16 | 0.62 |
| ggAs2:4w-17 | 17.72 | -188.81 | 700.56 | 470.00 | 410.04 | 2.80 | -0.14 | -3.51 |
| ggAs2:4w-18 | 18.54 | -187.98 | 779.10 | 451.84 | 393.98 | -2.90 | 2.97 | -1.21 |
| ggAs2:4w-19 | 18.63 | -187.91 | 641.85 | 480.88 | 442.88 | 2.20 | -2.19 | 0.77 |
| ggAs2:4w-20 | 18.65 | -187.88 | 765.34 | 483.95 | 387.95 | 4.56 | 0.73 | 1.09 |
| ggAs2:4w-21 | 18.98 | -187.54 | 824.25 | 411.94 | 348.20 | 4.04 | -2.44 | -1.16 |
| ggAs2:4w-22 | 19.58 | -186.95 | 792.30 | 380.11 | 324.61 | 0.29 | -0.82 | -0.16 |
| ggAs2:4w-23 | 19.76 | -186.77 | 671.12 | 503.11 | 396.54 | 0.23 | 2.05 | -0.27 |
| ggAs2:4w-24 | 20.06 | -186.47 | 769.21 | 398.03 | 334.01 | -1.19 | -1.10 | 2.48 |
| ggAs2:4w-25 | 20.30 | -186.23 | 635.26 | 499.58 | 429.69 | 3.09 | -1.11 | -1.21 |
| ggAs2:4w-26 | 20.56 | -185.96 | 673.01 | 477.59 | 395.26 | 0.90 | -0.35 | -3.35 |
| ggAs2:4w-27 | 20.92 | -185.61 | 639.43 | 501.48 | 410.89 | 3.32 | -0.60 | 0.39 |
| ggAs2:4w-28 | 21.84 | -184.68 | 860.24 | 390.25 | 321.56 | 4.44 | 0.17 | 0.90 |
| ggAs2:4w-29 | 22.10 | -184.43 | 672.36 | 456.39 | 359.32 | 4.11 | -0.99 | -0.94 |
| ggAs2:4w-30 | 22.12 | -184.41 | 819.19 | 414.32 | 352.81 | 1.55 | -2.77 | 2.49 |
| ggAs2:4w-31 | 22.23 | -184.31 | 733.41 | 412.82 | 363.69 | -2.40 | -2.00 | 0.19 |
| ggAs2:4w-32 | 22.32 | -184.20 | 806.73 | 400.60 | 347.33 | 3.71 | -2.99 | -3.13 |
| ggAs2:4w-33 | 22.39 | -184.14 | 792.08 | 427.90 | 391.35 | 5.77 | -2.61 | 1.75 |
| ggAs2:4w-34 | 22.39 | -184.14 | 753.41 | 454.50 | 399.54 | 2.41 | -2.64 | -1.36 |
| ggAs2:4w-35 | 22.42 | -184.12 | 657.95 | 471.62 | 406.15 | 2.00 | -0.81 | -0.22 |
| ggAs2:4w-36 | 22.99 | -183.53 | 692.38 | 474.21 | 387.85 | 0.52 | -1.16 | 0.83 |
| ggAs2:4w-37 | 23.08 | -183.46 | 715.59 | 464.46 | 409.39 | 5.93 | 0.04 | -0.30 |
| ggAs2:4w-38 | 23.69 | -182.85 | 783.91 | 442.42 | 387.19 | 4.68 | -1.04 | 0.39 |
| ggAs2:4w-39 | 23.84 | -182.69 | 739.61 | 403.81 | 332.22 | 3.89 | 0.72 | 0.77 |
| ggAs2:4w-40 | 24.50 | -182.02 | 927.77 | 341.70 | 304.35 | 3.76 | -3.61 | 0.91 |
| ggAs2:4w-41 | 25.42 | -181.11 | 616.94 | 520.18 | 355.24 | 1.05 | -3.05 | -3.26 |
| ggAs2:4w-42 | 25.94 | -180.60 | 608.75 | 518.07 | 366.14 | 4.88 | -1.40 | -1.35 |
| ggAs2:4w-43 | 26.01 | -180.52 | 688.87 | 471.53 | 373.85 | 4.62 | -0.94 | 1.11 |
| ggAs2:4w-44 | 26.02 | -180.51 | 653.92 | 444.79 | 324.22 | 4.19 | -1.64 | 1.07 |
| ggAs2:4w-45 | 26.31 | -180.23 | 608.81 | 474.51 | 428.21 | 4.13 | -0.14 | 0.38 |
| ggAs2:4w-46 | 26.58 | -179.96 | 647.53 | 493.65 | 425.17 | 3.88 | 0.29 | -2.70 |
| ggAs2:4w-47 | 27.71 | -178.82 | 781.81 | 358.59 | 312.04 | 4.30 | -2.63 | 2.18 |

Table S31: Theoretical (B3LYP-D3(BJ)/def2-TZVP) energy and spectroscopic parameters of the tetra-hydrated species of DEAcA. *(continued)*

| Conformer | $E_e$ / kJ·mol$^{-1}$ | $BE$ / kJ·mol$^{-1}$ | $A$ / MHz | $B$ / MHz | $C$ / MHz | $\mu_a$ / D | $\mu_b$ / D | $\mu_c$ / D |
|---|---|---|---|---|---|---|---|---|
| ggAs2:4w-48 | 29.58 | -176.95 | 875.49 | 319.97 | 285.87 | 4.82 | -1.41 | -0.63 |
| ggAs2:4w-49 | 30.13 | -176.40 | 631.67 | 485.93 | 367.57 | 6.66 | 3.11 | 2.24 |
| ggAs2:4w-50 | 30.34 | -176.19 | 894.68 | 358.94 | 306.48 | 0.42 | -1.72 | -1.78 |
| ggAs2:4w-51 | 34.56 | -171.97 | 769.72 | 400.46 | 347.32 | 7.89 | -0.73 | -1.21 |
| ggAs2:4w-52 | 35.97 | -170.56 | 831.66 | 363.22 | 310.78 | -1.95 | -1.60 | 0.83 |
| ggAs2:4w-53 | 36.28 | -170.25 | 621.64 | 462.87 | 362.16 | -1.46 | 1.97 | -0.78 |
| ggAs2:4w-54 | 40.77 | -165.76 | 1090.13 | 240.60 | 232.78 | -5.37 | -0.16 | 0.33 |

# 14 Experimental rotational transition frequencies

In the following section, the observed rotational transition lines are listed for each assigned species. For each transition, the tables report the quantum numbers of the upper and lower rotational levels involved, ($J'$ $K'_a$ $K'_c$ $F'$ - $J''$ $K''_a$ $K''_c$ $F''$), the measured frequency $\nu_{\rm obs}$, the residual $\nu_{\rm obs} - \nu_{\rm calc}$ obtained from the fitted spectroscopic parameters, and the experimental uncertainty (Err.) associated with the spectrometer used for the measurement (Table S1). Frequencies, residuals, and uncertainties are reported in MHz.

Table S32: Experimental rotational transition frequencies (MHz) for #1-DEAcA (aaAs).

| $J'$ | $K'_a$ | $K'_c$ | $F'$ | - | $J''$ | $K''_a$ | $K''_c$ | $F''$ | $\nu_{\rm obs}$ | $\nu_{\rm obs} - \nu_{\rm calc}$ | Err. |
|---|---|---|---|---|---|---|---|---|---|---|---|
| 1 | 0 | 1 | 0 | | 0 | 0 | 0 | 1 | 2396.720 | -0.007 | 0.010 |
| 1 | 0 | 1 | 2 | | 0 | 0 | 0 | 1 | 2398.812 | -0.001 | 0.010 |
| 1 | 0 | 1 | 1 | | 0 | 0 | 0 | 1 | 2400.200 | -0.003 | 0.010 |
| 2 | 2 | 1 | 2 | | 2 | 0 | 2 | 2 | 2404.629 | -0.002 | 0.010 |
| 2 | 2 | 1 | 3 | | 2 | 0 | 2 | 3 | 2407.945 | -0.007 | 0.010 |
| 4 | 2 | 2 | 4 | | 4 | 2 | 3 | 4 | 2412.258 | -0.003 | 0.010 |
| 4 | 2 | 2 | 5 | | 4 | 2 | 3 | 5 | 2413.192 | -0.000 | 0.010 |
| 4 | 2 | 2 | 3 | | 4 | 2 | 3 | 3 | 2413.440 | 0.008 | 0.010 |
| 2 | 2 | 0 | 2 | | 2 | 1 | 2 | 2 | 2617.054 | 0.001 | 0.010 |
| 2 | 2 | 0 | 3 | | 2 | 1 | 2 | 3 | 2620.557 | 0.001 | 0.010 |
| 2 | 2 | 0 | 1 | | 2 | 1 | 2 | 1 | 2622.504 | 0.001 | 0.010 |
| 4 | 4 | 1 | 4 | | 4 | 3 | 1 | 4 | 2673.747 | -0.005 | 0.010 |
| 4 | 4 | 1 | 5 | | 4 | 3 | 1 | 5 | 2675.059 | -0.000 | 0.010 |
| 4 | 4 | 1 | 3 | | 4 | 3 | 1 | 3 | 2675.389 | -0.006 | 0.010 |
| 3 | 1 | 2 | 3 | | 3 | 1 | 3 | 3 | 2809.418 | 0.004 | 0.010 |
| 3 | 1 | 2 | 4 | | 3 | 1 | 3 | 4 | 2811.262 | -0.003 | 0.010 |
| 3 | 1 | 2 | 2 | | 3 | 1 | 3 | 2 | 2811.912 | -0.002 | 0.010 |
| 3 | 3 | 0 | 3 | | 3 | 2 | 2 | 3 | 2929.597 | -0.005 | 0.010 |
| 3 | 3 | 0 | 4 | | 3 | 2 | 2 | 4 | 2931.696 | -0.001 | 0.010 |
| 3 | 3 | 0 | 2 | | 3 | 2 | 2 | 2 | 2932.430 | 0.001 | 0.010 |
| 6 | 5 | 2 | 6 | | 6 | 4 | 2 | 6 | 2995.542 | -0.003 | 0.010 |
| 6 | 5 | 2 | 7 | | 6 | 4 | 2 | 7 | 2996.295 | -0.001 | 0.010 |
| 6 | 5 | 2 | 5 | | 6 | 4 | 2 | 5 | 2996.408 | -0.015 | 0.010 |
| 1 | 1 | 0 | 1 | | 0 | 0 | 0 | 1 | 3170.237 | 0.001 | 0.010 |
| 1 | 1 | 0 | 2 | | 0 | 0 | 0 | 1 | 3171.797 | 0.001 | 0.010 |
| 1 | 1 | 0 | 0 | | 0 | 0 | 0 | 1 | 3174.145 | 0.007 | 0.010 |
| 10 | 7 | 4 | 10 | | 10 | 6 | 4 | 10 | 3185.039 | 0.001 | 0.010 |
| 10 | 7 | 4 | 11 | | 10 | 6 | 4 | 11 | 3185.392 | 0.001 | 0.010 |
| 3 | 3 | 1 | 4 | | 3 | 1 | 2 | 4 | 3188.507 | 0.005 | 0.010 |
| 3 | 3 | 1 | 2 | | 3 | 1 | 2 | 2 | 3189.320 | -0.016 | 0.010 |
| 3 | 2 | 2 | 3 | | 3 | 0 | 3 | 3 | 3229.280 | 0.000 | 0.010 |
| 3 | 2 | 2 | 4 | | 3 | 0 | 3 | 4 | 3231.514 | 0.002 | 0.010 |
| 3 | 2 | 2 | 2 | | 3 | 0 | 3 | 2 | 3232.295 | 0.001 | 0.010 |
| 4 | 4 | 0 | 4 | | 4 | 3 | 2 | 4 | 3467.759 | -0.006 | 0.010 |
| 4 | 4 | 0 | 5 | | 4 | 3 | 2 | 5 | 3469.332 | -0.000 | 0.010 |
| 4 | 4 | 0 | 3 | | 4 | 3 | 2 | 3 | 3469.735 | 0.000 | 0.010 |
| 4 | 3 | 2 | 4 | | 4 | 1 | 3 | 4 | 3543.584 | -0.002 | 0.010 |
| 4 | 3 | 2 | 5 | | 4 | 1 | 3 | 5 | 3545.137 | -0.001 | 0.010 |
| 4 | 3 | 2 | 3 | | 4 | 1 | 3 | 3 | 3545.537 | -0.001 | 0.010 |
| 2 | 0 | 2 | 1 | | 1 | 1 | 0 | 0 | 3719.256 | -0.009 | 0.010 |
| 2 | 0 | 2 | 3 | | 1 | 1 | 0 | 2 | 3722.617 | -0.006 | 0.010 |
| 2 | 0 | 2 | 1 | | 1 | 1 | 0 | 1 | 3723.158 | -0.008 | 0.010 |
| 2 | 0 | 2 | 2 | | 1 | 1 | 0 | 2 | 3724.452 | -0.002 | 0.010 |
| 2 | 0 | 2 | 2 | | 1 | 1 | 0 | 1 | 3726.008 | -0.007 | 0.010 |
| 5 | 5 | 1 | 5 | | 5 | 4 | 1 | 5 | 3762.138 | -0.000 | 0.010 |
| 5 | 5 | 1 | 6 | | 5 | 4 | 1 | 6 | 3763.364 | -0.001 | 0.010 |
| 5 | 5 | 1 | 4 | | 5 | 4 | 1 | 4 | 3763.618 | 0.003 | 0.010 |
| 5 | 2 | 3 | 5 | | 5 | 2 | 4 | 5 | 3911.449 | -0.002 | 0.010 |
| 5 | 2 | 3 | 6 | | 5 | 2 | 4 | 6 | 3912.495 | -0.012 | 0.010 |

Table S32: Experimental rotational transition frequencies (MHz) for #1-DEAcA *(continued)*

| $J'$ | $K_a'$ | $K_c'$ | $F'$ | - | $J''$ | $K_a''$ | $K_c''$ | $F''$ | $\nu_{\text{obs}}$ | $\nu_{\text{obs}} - \nu_{\text{calc}}$ | Err. |
|---|---|---|---|---|---|---|---|---|---|---|---|
| 5 | 2 | 3 | 4 | | 5 | 2 | 4 | 4 | 3912.719 | -0.003 | 0.010 |
| 4 | 3 | 1 | 4 | | 4 | 2 | 3 | 4 | 4155.418 | -0.002 | 0.010 |
| 4 | 3 | 1 | 5 | | 4 | 2 | 3 | 5 | 4157.131 | -0.007 | 0.010 |
| 4 | 3 | 1 | 3 | | 4 | 2 | 3 | 3 | 4157.585 | 0.005 | 0.010 |
| 5 | 4 | 1 | 5 | | 5 | 3 | 3 | 5 | 4180.536 | -0.006 | 0.010 |
| 5 | 4 | 1 | 6 | | 5 | 3 | 3 | 6 | 4181.736 | -0.011 | 0.010 |
| 5 | 4 | 1 | 4 | | 5 | 3 | 3 | 4 | 4181.984 | -0.008 | 0.010 |
| 5 | 5 | 0 | 5 | | 5 | 4 | 2 | 5 | 4184.041 | 0.005 | 0.010 |
| 5 | 5 | 0 | 6 | | 5 | 4 | 2 | 6 | 4185.328 | -0.012 | 0.010 |
| 5 | 5 | 0 | 4 | | 5 | 4 | 2 | 4 | 4185.607 | 0.001 | 0.010 |
| 4 | 1 | 3 | 4 | | 4 | 1 | 4 | 4 | 4186.935 | -0.002 | 0.010 |
| 4 | 1 | 3 | 5 | | 4 | 1 | 4 | 5 | 4188.642 | -0.016 | 0.010 |
| 4 | 1 | 3 | 3 | | 4 | 1 | 4 | 3 | 4189.088 | -0.013 | 0.010 |
| 7 | 6 | 2 | 6 | | 7 | 5 | 2 | 6 | 4196.372 | -0.005 | 0.010 |
| 5 | 4 | 2 | 5 | | 5 | 2 | 3 | 5 | 4214.797 | -0.011 | 0.010 |
| 5 | 4 | 2 | 6 | | 5 | 2 | 3 | 6 | 4216.121 | 0.002 | 0.010 |
| 5 | 4 | 2 | 4 | | 5 | 2 | 3 | 4 | 4216.364 | -0.022 | 0.010 |
| 2 | 1 | 2 | 1 | | 1 | 1 | 1 | 1 | 4304.109 | -0.000 | 0.010 |
| 2 | 1 | 2 | 1 | | 1 | 1 | 1 | 2 | 4304.299 | 0.021 | 0.010 |
| 2 | 1 | 2 | 1 | | 1 | 1 | 1 | 0 | 4304.532 | -0.001 | 0.010 |
| 2 | 1 | 2 | 3 | | 1 | 1 | 1 | 2 | 4305.206 | -0.001 | 0.010 |
| 2 | 1 | 2 | 2 | | 1 | 1 | 1 | 1 | 4306.710 | 0.001 | 0.010 |
| 2 | 1 | 2 | 2 | | 1 | 1 | 1 | 2 | 4306.875 | -0.004 | 0.010 |
| 4 | 2 | 3 | 4 | | 4 | 0 | 4 | 4 | 4315.235 | 0.003 | 0.010 |
| 4 | 2 | 3 | 5 | | 4 | 0 | 4 | 5 | 4317.038 | 0.003 | 0.010 |
| 4 | 2 | 3 | 3 | | 4 | 0 | 4 | 3 | 4317.494 | -0.004 | 0.010 |
| 3 | 2 | 1 | 3 | | 3 | 1 | 3 | 3 | 4335.813 | -0.002 | 0.010 |
| 3 | 2 | 1 | 4 | | 3 | 1 | 3 | 4 | 4338.718 | 0.003 | 0.010 |
| 3 | 2 | 1 | 2 | | 3 | 1 | 3 | 2 | 4339.721 | -0.009 | 0.010 |
| 5 | 3 | 3 | 5 | | 5 | 1 | 4 | 5 | 4376.146 | 0.002 | 0.010 |
| 5 | 3 | 3 | 6 | | 5 | 1 | 4 | 6 | 4377.399 | 0.004 | 0.010 |
| 5 | 3 | 3 | 4 | | 5 | 1 | 4 | 4 | 4377.657 | 0.007 | 0.010 |
| 6 | 5 | 1 | 6 | | 6 | 4 | 3 | 6 | 4487.577 | -0.014 | 0.010 |
| 6 | 5 | 1 | 7 | | 6 | 4 | 3 | 7 | 4488.574 | 0.012 | 0.010 |
| 6 | 5 | 1 | 5 | | 6 | 4 | 3 | 5 | 4488.759 | 0.033 | 0.010 |
| 2 | 0 | 2 | 1 | | 1 | 0 | 1 | 1 | 4493.195 | -0.004 | 0.010 |
| 2 | 0 | 2 | 3 | | 1 | 0 | 1 | 2 | 4495.603 | -0.004 | 0.010 |
| 2 | 0 | 2 | 2 | | 1 | 0 | 1 | 1 | 4496.046 | -0.002 | 0.010 |
| 2 | 0 | 2 | 1 | | 1 | 0 | 1 | 0 | 4496.681 | 0.006 | 0.010 |
| 2 | 0 | 2 | 2 | | 1 | 0 | 1 | 2 | 4497.435 | -0.003 | 0.010 |
| 8 | 4 | 4 | 8 | | 8 | 4 | 5 | 8 | 4498.188 | 0.003 | 0.010 |
| 8 | 4 | 4 | 9 | | 8 | 4 | 5 | 9 | 4498.674 | 0.003 | 0.010 |
| 8 | 4 | 4 | 7 | | 8 | 4 | 5 | 7 | 4498.735 | 0.003 | 0.010 |
| 3 | 0 | 3 | 2 | | 2 | 1 | 1 | 1 | 4804.485 | -0.012 | 0.010 |
| 3 | 0 | 3 | 2 | | 2 | 1 | 1 | 2 | 4804.777 | -0.005 | 0.010 |
| 3 | 0 | 3 | 4 | | 2 | 1 | 1 | 3 | 4805.372 | -0.009 | 0.010 |
| 3 | 0 | 3 | 3 | | 2 | 1 | 1 | 2 | 4807.788 | -0.007 | 0.010 |
| 6 | 6 | 1 | 7 | | 6 | 5 | 1 | 6 | 4824.435 | 0.003 | 0.010 |
| 6 | 6 | 1 | 5 | | 6 | 5 | 1 | 6 | 4824.877 | 0.003 | 0.010 |
| 6 | 6 | 0 | 6 | | 6 | 5 | 2 | 6 | 5012.866 | -0.023 | 0.010 |
| 6 | 6 | 0 | 7 | | 6 | 5 | 2 | 7 | 5013.993 | -0.035 | 0.010 |
| 6 | 6 | 0 | 5 | | 6 | 5 | 2 | 5 | 5014.206 | -0.015 | 0.010 |
| 2 | 1 | 1 | 1 | | 1 | 1 | 0 | 0 | 5288.120 | -0.008 | 0.010 |
| 2 | 1 | 1 | 2 | | 1 | 1 | 0 | 2 | 5290.185 | 0.000 | 0.010 |
| 2 | 1 | 1 | 3 | | 1 | 1 | 0 | 2 | 5290.364 | -0.003 | 0.010 |
| 2 | 1 | 1 | 2 | | 1 | 1 | 0 | 1 | 5291.746 | 0.001 | 0.010 |
| 2 | 1 | 1 | 1 | | 1 | 1 | 0 | 1 | 5292.026 | -0.004 | 0.010 |

Table S32: Experimental rotational transition frequencies (MHz) for #1-DEAcA *(continued)*

| $J'$ | $K_a'$ | $K_c'$ | $F'$ | - | $J''$ | $K_a''$ | $K_c''$ | $F''$ | $\nu_{\rm obs}$ | $\nu_{\rm obs}-\nu_{\rm calc}$ | Err. |
|---|---|---|---|---|---|---|---|---|---|---|---|
| 6 | 3 | 4 | 6 | | 6 | 1 | 5 | 6 | 5474.649 | -0.030 | 0.010 |
| 6 | 3 | 4 | 7 | | 6 | 1 | 5 | 7 | 5475.771 | -0.022 | 0.010 |
| 6 | 3 | 4 | 5 | | 6 | 1 | 5 | 5 | 5475.919 | -0.062 | 0.010 |
| 2 | 1 | 1 | 2 | | 1 | 0 | 1 | 1 | 6061.773 | -0.005 | 0.010 |
| 2 | 1 | 1 | 1 | | 1 | 0 | 1 | 1 | 6062.055 | -0.007 | 0.010 |
| 2 | 1 | 1 | 2 | | 1 | 0 | 1 | 2 | 6063.156 | -0.012 | 0.010 |
| 2 | 1 | 1 | 3 | | 1 | 0 | 1 | 2 | 6063.343 | -0.007 | 0.010 |
| 2 | 1 | 1 | 1 | | 1 | 0 | 1 | 0 | 6065.540 | 0.002 | 0.010 |
| 5 | 3 | 2 | 5 | | 5 | 2 | 4 | 5 | 6232.238 | -0.035 | 0.010 |
| 5 | 3 | 2 | 6 | | 5 | 2 | 4 | 6 | 6233.940 | -0.027 | 0.010 |
| 5 | 3 | 2 | 4 | | 5 | 2 | 4 | 4 | 6234.260 | -0.052 | 0.010 |
| 3 | 1 | 3 | 2 | | 2 | 1 | 2 | 2 | 6300.014 | -0.008 | 0.010 |
| 3 | 1 | 3 | 4 | | 2 | 1 | 2 | 3 | 6302.464 | -0.004 | 0.010 |
| 3 | 1 | 3 | 2 | | 2 | 1 | 2 | 1 | 6302.619 | -0.004 | 0.010 |
| 3 | 1 | 3 | 3 | | 2 | 1 | 2 | 2 | 6303.004 | -0.005 | 0.010 |
| 3 | 1 | 3 | 3 | | 2 | 1 | 2 | 3 | 6304.674 | -0.007 | 0.010 |
| 3 | 0 | 3 | 2 | | 2 | 0 | 2 | 2 | 6370.507 | -0.005 | 0.010 |
| 3 | 0 | 3 | 4 | | 2 | 0 | 2 | 3 | 6373.118 | -0.006 | 0.010 |
| 3 | 0 | 3 | 2 | | 2 | 0 | 2 | 1 | 6373.352 | -0.009 | 0.010 |
| 3 | 0 | 3 | 3 | | 2 | 0 | 2 | 2 | 6373.518 | -0.007 | 0.010 |
| 3 | 0 | 3 | 3 | | 2 | 0 | 2 | 3 | 6375.350 | -0.007 | 0.010 |
| 2 | 2 | 0 | 2 | | 1 | 1 | 0 | 2 | 6431.122 | -0.002 | 0.010 |
| 2 | 2 | 0 | 1 | | 1 | 1 | 0 | 0 | 6431.623 | -0.009 | 0.010 |
| 2 | 2 | 0 | 2 | | 1 | 1 | 0 | 1 | 6432.680 | -0.004 | 0.010 |
| 2 | 2 | 0 | 3 | | 1 | 1 | 0 | 2 | 6432.953 | -0.002 | 0.010 |
| 2 | 2 | 0 | 1 | | 1 | 1 | 0 | 2 | 6433.967 | -0.006 | 0.010 |
| 2 | 2 | 0 | 1 | | 1 | 1 | 0 | 1 | 6435.531 | -0.003 | 0.010 |
| 3 | 1 | 2 | 2 | | 2 | 2 | 0 | 1 | 6492.027 | -0.007 | 0.010 |
| 3 | 1 | 2 | 4 | | 2 | 2 | 0 | 3 | 6493.177 | -0.001 | 0.010 |
| 3 | 1 | 2 | 3 | | 2 | 2 | 0 | 3 | 6493.526 | -0.013 | 0.010 |
| 3 | 1 | 2 | 2 | | 2 | 2 | 0 | 2 | 6494.877 | -0.007 | 0.010 |
| 3 | 1 | 2 | 3 | | 2 | 2 | 0 | 2 | 6495.365 | -0.005 | 0.010 |
| 2 | 2 | 1 | 2 | | 1 | 1 | 1 | 1 | 6621.720 | -0.003 | 0.010 |
| 2 | 2 | 1 | 2 | | 1 | 1 | 1 | 2 | 6621.893 | 0.000 | 0.010 |
| 2 | 2 | 1 | 3 | | 1 | 1 | 1 | 2 | 6623.379 | -0.004 | 0.010 |
| 2 | 2 | 1 | 1 | | 1 | 1 | 1 | 1 | 6624.040 | -0.001 | 0.010 |
| 2 | 2 | 1 | 1 | | 1 | 1 | 1 | 0 | 6624.462 | -0.004 | 0.010 |
| 3 | 2 | 2 | 2 | | 2 | 2 | 1 | 1 | 7195.855 | -0.002 | 0.003 |
| 3 | 2 | 2 | 4 | | 2 | 2 | 1 | 3 | 7196.685 | 0.001 | 0.003 |
| 3 | 2 | 2 | 3 | | 2 | 2 | 1 | 2 | 7198.173 | -0.001 | 0.003 |
| 2 | 2 | 0 | 2 | | 1 | 0 | 1 | 1 | 7202.708 | -0.009 | 0.010 |
| 2 | 2 | 0 | 2 | | 1 | 0 | 1 | 2 | 7204.084 | -0.024 | 0.010 |
| 2 | 2 | 0 | 1 | | 1 | 0 | 1 | 1 | 7205.564 | -0.002 | 0.010 |
| 2 | 2 | 0 | 3 | | 1 | 0 | 1 | 2 | 7205.930 | -0.009 | 0.010 |
| 2 | 2 | 0 | 1 | | 1 | 0 | 1 | 0 | 7209.024 | -0.018 | 0.010 |
| 3 | 1 | 2 | 2 | | 2 | 1 | 1 | 1 | 7635.538 | -0.000 | 0.003 |
| 3 | 1 | 2 | 4 | | 2 | 1 | 1 | 3 | 7635.767 | 0.000 | 0.003 |
| 3 | 1 | 2 | 3 | | 2 | 1 | 1 | 2 | 7636.309 | -0.001 | 0.003 |
| 3 | 2 | 1 | 3 | | 2 | 2 | 0 | 3 | 8019.936 | -0.004 | 0.003 |
| 3 | 2 | 1 | 4 | | 2 | 2 | 0 | 3 | 8020.626 | -0.001 | 0.003 |
| 3 | 2 | 1 | 3 | | 2 | 2 | 0 | 2 | 8021.765 | -0.006 | 0.003 |
| 3 | 2 | 1 | 2 | | 2 | 2 | 0 | 2 | 8022.709 | 0.009 | 0.003 |
| 4 | 1 | 4 | 3 | | 3 | 1 | 3 | 3 | 8233.372 | 0.002 | 0.003 |
| 4 | 1 | 4 | 5 | | 3 | 1 | 3 | 4 | 8236.231 | 0.000 | 0.003 |
| 4 | 1 | 4 | 3 | | 3 | 1 | 3 | 2 | 8236.355 | -0.001 | 0.003 |
| 4 | 1 | 4 | 4 | | 3 | 1 | 3 | 3 | 8236.542 | 0.000 | 0.003 |
| 4 | 1 | 4 | 4 | | 3 | 1 | 3 | 4 | 8238.754 | -0.000 | 0.003 |

Table S32: Experimental rotational transition frequencies (MHz) for #1-DEAcA *(continued)*

| $J'$ | $K_a'$ | $K_c'$ | $F'$ | - | $J''$ | $K_a''$ | $K_c''$ | $F''$ | $\nu_{\text{obs}}$ | $\nu_{\text{obs}} - \nu_{\text{calc}}$ | Err. |
|---|---|---|---|---|---|---|---|---|---|---|---|
| 4 | 0 | 4 | 5 | | 3 | 0 | 3 | 4 | 8252.004 | -0.001 | 0.003 |
| 4 | 0 | 4 | 3 | | 3 | 0 | 3 | 2 | 8252.133 | -0.004 | 0.003 |
| 4 | 0 | 4 | 4 | | 3 | 0 | 3 | 3 | 8252.295 | -0.004 | 0.003 |
| 3 | 2 | 1 | 3 | | 2 | 1 | 1 | 3 | 9162.528 | -0.001 | 0.003 |
| 3 | 2 | 1 | 3 | | 2 | 1 | 1 | 2 | 9162.710 | -0.000 | 0.003 |
| 3 | 2 | 1 | 4 | | 2 | 1 | 1 | 3 | 9163.216 | 0.000 | 0.003 |
| 3 | 2 | 1 | 2 | | 2 | 1 | 1 | 1 | 9163.351 | -0.004 | 0.003 |
| 3 | 2 | 1 | 2 | | 2 | 1 | 1 | 2 | 9163.636 | -0.003 | 0.003 |
| 3 | 1 | 2 | 2 | | 2 | 0 | 2 | 2 | 9201.551 | -0.002 | 0.003 |
| 3 | 1 | 2 | 3 | | 2 | 0 | 2 | 2 | 9202.041 | 0.001 | 0.003 |
| 3 | 1 | 2 | 4 | | 2 | 0 | 2 | 3 | 9203.511 | 0.001 | 0.003 |
| 3 | 1 | 2 | 3 | | 2 | 0 | 2 | 3 | 9203.872 | 0.001 | 0.003 |
| 3 | 1 | 2 | 2 | | 2 | 0 | 2 | 1 | 9204.403 | 0.001 | 0.003 |
| 3 | 2 | 2 | 3 | | 2 | 1 | 2 | 2 | 9513.189 | 0.001 | 0.003 |
| 3 | 2 | 2 | 4 | | 2 | 1 | 2 | 3 | 9514.861 | 0.001 | 0.003 |
| 3 | 2 | 2 | 2 | | 2 | 1 | 2 | 1 | 9515.789 | -0.000 | 0.003 |
| 4 | 1 | 3 | 3 | | 3 | 1 | 2 | 2 | 9613.541 | -0.002 | 0.003 |
| 4 | 1 | 3 | 5 | | 3 | 1 | 2 | 4 | 9613.623 | -0.000 | 0.003 |
| 4 | 1 | 3 | 4 | | 3 | 1 | 2 | 3 | 9614.064 | -0.001 | 0.003 |
| 3 | 3 | 0 | 3 | | 2 | 2 | 0 | 3 | 9823.906 | 0.000 | 0.003 |
| 3 | 3 | 0 | 2 | | 2 | 2 | 0 | 1 | 9825.712 | -0.004 | 0.003 |
| 3 | 3 | 0 | 3 | | 2 | 2 | 0 | 2 | 9825.739 | 0.002 | 0.003 |
| 3 | 3 | 0 | 4 | | 2 | 2 | 0 | 3 | 9826.002 | 0.001 | 0.003 |
| 3 | 3 | 0 | 2 | | 2 | 2 | 0 | 2 | 9828.565 | -0.001 | 0.003 |
| 3 | 3 | 1 | 3 | | 2 | 2 | 1 | 3 | 9982.038 | 0.001 | 0.003 |
| 3 | 3 | 1 | 3 | | 2 | 2 | 1 | 2 | 9983.527 | 0.000 | 0.003 |
| 3 | 3 | 1 | 2 | | 2 | 2 | 1 | 1 | 9983.942 | 0.001 | 0.003 |
| 3 | 3 | 1 | 4 | | 2 | 2 | 1 | 3 | 9984.061 | 0.001 | 0.003 |
| 3 | 3 | 1 | 2 | | 2 | 2 | 1 | 2 | 9986.260 | 0.001 | 0.003 |
| 5 | 1 | 5 | 6 | | 4 | 1 | 4 | 5 | 10148.554 | -0.000 | 0.003 |
| 5 | 1 | 5 | 4 | | 4 | 1 | 4 | 3 | 10148.640 | -0.006 | 0.003 |
| 5 | 1 | 5 | 5 | | 4 | 1 | 4 | 4 | 10148.763 | -0.003 | 0.003 |
| 5 | 1 | 4 | 4 | | 4 | 1 | 3 | 3 | 11442.474 | -0.002 | 0.003 |
| 5 | 1 | 4 | 6 | | 4 | 1 | 3 | 5 | 11442.512 | -0.002 | 0.003 |
| 5 | 1 | 4 | 5 | | 4 | 1 | 3 | 4 | 11442.907 | -0.002 | 0.003 |
| 4 | 2 | 2 | 4 | | 3 | 1 | 2 | 3 | 12151.276 | -0.001 | 0.003 |
| 4 | 2 | 2 | 3 | | 3 | 1 | 2 | 3 | 12151.543 | 0.003 | 0.003 |
| 4 | 2 | 2 | 4 | | 3 | 1 | 2 | 4 | 12151.638 | 0.000 | 0.003 |
| 4 | 2 | 2 | 5 | | 3 | 1 | 2 | 4 | 12151.846 | -0.000 | 0.003 |
| 4 | 2 | 2 | 3 | | 3 | 1 | 2 | 2 | 12152.023 | -0.003 | 0.003 |
| 4 | 3 | 1 | 4 | | 3 | 2 | 1 | 4 | 12367.349 | 0.001 | 0.003 |
| 4 | 3 | 1 | 4 | | 3 | 2 | 1 | 3 | 12368.034 | -0.001 | 0.003 |
| 4 | 3 | 1 | 5 | | 3 | 2 | 1 | 4 | 12368.343 | -0.000 | 0.003 |
| 4 | 3 | 1 | 3 | | 3 | 2 | 1 | 2 | 12368.361 | 0.003 | 0.003 |
| 4 | 3 | 1 | 3 | | 3 | 2 | 1 | 3 | 12369.288 | 0.001 | 0.003 |
| 4 | 1 | 3 | 3 | | 3 | 0 | 3 | 3 | 12441.570 | -0.001 | 0.003 |
| 4 | 1 | 3 | 4 | | 3 | 0 | 3 | 3 | 12442.580 | 0.001 | 0.003 |
| 4 | 1 | 3 | 5 | | 3 | 0 | 3 | 4 | 12444.010 | 0.001 | 0.003 |
| 4 | 1 | 3 | 3 | | 3 | 0 | 3 | 2 | 12444.585 | 0.001 | 0.003 |
| 4 | 1 | 3 | 4 | | 3 | 0 | 3 | 4 | 12444.811 | -0.000 | 0.003 |
| 4 | 2 | 3 | 3 | | 3 | 1 | 3 | 3 | 12547.520 | -0.002 | 0.003 |
| 4 | 2 | 3 | 4 | | 3 | 1 | 3 | 3 | 12548.430 | -0.000 | 0.003 |
| 4 | 2 | 3 | 5 | | 3 | 1 | 3 | 4 | 12549.918 | -0.002 | 0.003 |
| 4 | 2 | 3 | 3 | | 3 | 1 | 3 | 2 | 12550.508 | -0.000 | 0.003 |
| 4 | 2 | 3 | 4 | | 3 | 1 | 3 | 4 | 12550.650 | 0.007 | 0.003 |
| 4 | 3 | 2 | 4 | | 3 | 2 | 2 | 3 | 12756.886 | 0.000 | 0.003 |
| 4 | 3 | 2 | 5 | | 3 | 2 | 2 | 4 | 12757.635 | -0.001 | 0.003 |

Table S32: Experimental rotational transition frequencies (MHz) for #1-DEAcA *(continued)*

| $J'$ | $K'_a$ | $K'_c$ | $F'$ | - | $J''$ | $K''_a$ | $K''_c$ | $F''$ | $\nu_{\text{obs}}$ | $\nu_{\text{obs}} - \nu_{\text{calc}}$ | Err. |
|---|---|---|---|---|---|---|---|---|---|---|---|
| 4 | 3 | 2 | 3 | | 3 | 2 | 2 | 2 | 12757.826 | -0.002 | 0.003 |
| 4 | 4 | 0 | 4 | | 3 | 3 | 0 | 4 | 13292.952 | -0.002 | 0.003 |
| 4 | 4 | 0 | 4 | | 3 | 3 | 0 | 3 | 13295.050 | 0.001 | 0.003 |
| 4 | 4 | 0 | 3 | | 3 | 3 | 0 | 2 | 13295.136 | 0.002 | 0.003 |
| 4 | 4 | 0 | 5 | | 3 | 3 | 0 | 4 | 13295.281 | 0.010 | 0.003 |
| 4 | 4 | 1 | 4 | | 3 | 3 | 1 | 4 | 13380.046 | -0.001 | 0.003 |
| 4 | 4 | 1 | 4 | | 3 | 3 | 1 | 3 | 13382.071 | 0.001 | 0.003 |
| 4 | 4 | 1 | 3 | | 3 | 3 | 1 | 2 | 13382.237 | 0.003 | 0.003 |
| 4 | 4 | 1 | 5 | | 3 | 3 | 1 | 4 | 13382.352 | 0.002 | 0.003 |
| 4 | 4 | 1 | 3 | | 3 | 3 | 1 | 3 | 13384.968 | 0.002 | 0.003 |
| 5 | 3 | 2 | 5 | | 4 | 2 | 2 | 4 | 15166.773 | 0.001 | 0.003 |
| 5 | 3 | 2 | 6 | | 4 | 2 | 2 | 5 | 15167.075 | 0.001 | 0.003 |
| 5 | 3 | 2 | 4 | | 4 | 2 | 2 | 3 | 15167.123 | -0.001 | 0.003 |
| 5 | 2 | 3 | 6 | | 4 | 1 | 3 | 5 | 15383.836 | -0.000 | 0.003 |
| 5 | 2 | 3 | 4 | | 4 | 1 | 3 | 3 | 15384.013 | -0.004 | 0.003 |
| 5 | 1 | 4 | 4 | | 4 | 0 | 4 | 4 | 15631.760 | 0.011 | 0.003 |
| 5 | 1 | 4 | 5 | | 4 | 0 | 4 | 4 | 15633.194 | 0.004 | 0.003 |
| 5 | 1 | 4 | 6 | | 4 | 0 | 4 | 5 | 15634.520 | 0.002 | 0.003 |
| 5 | 1 | 4 | 4 | | 4 | 0 | 4 | 3 | 15634.930 | 0.006 | 0.003 |
| 5 | 1 | 4 | 5 | | 4 | 0 | 4 | 5 | 15635.714 | -0.002 | 0.003 |
| 5 | 2 | 4 | 5 | | 4 | 1 | 4 | 4 | 15658.652 | 0.004 | 0.003 |
| 5 | 2 | 4 | 6 | | 4 | 1 | 4 | 5 | 15659.988 | 0.000 | 0.003 |
| 5 | 2 | 4 | 4 | | 4 | 1 | 4 | 3 | 15660.395 | -0.000 | 0.003 |
| 5 | 5 | 0 | 5 | | 4 | 4 | 0 | 4 | 16770.655 | 0.000 | 0.003 |
| 5 | 5 | 0 | 4 | | 4 | 4 | 0 | 3 | 16770.736 | 0.000 | 0.003 |
| 5 | 5 | 0 | 6 | | 4 | 4 | 0 | 5 | 16770.826 | 0.001 | 0.003 |
| 18 | 16 | 2 | 18 | | 17 | 15 | 2 | 17 | 59769.970 | 0.017 | 0.050 |
| 18 | 16 | 2 | 17 | | 17 | 15 | 2 | 16 | 59769.970 | -0.043 | 0.050 |
| 18 | 16 | 3 | 19 | | 17 | 15 | 3 | 18 | 59769.970 | -0.074 | 0.050 |
| 18 | 16 | 3 | 19 | | 17 | 15 | 3 | 18 | 59769.970 | -0.074 | 0.050 |
| 18 | 16 | 3 | 18 | | 17 | 15 | 3 | 17 | 59769.970 | -0.011 | 0.050 |
| 18 | 16 | 2 | 17 | | 17 | 15 | 2 | 16 | 59769.970 | -0.043 | 0.050 |
| 20 | 12 | 8 | 20 | | 19 | 11 | 8 | 19 | 60247.870 | 0.159 | 0.050 |
| 20 | 12 | 8 | 21 | | 19 | 11 | 8 | 20 | 60247.870 | 0.101 | 0.050 |
| 20 | 12 | 8 | 19 | | 19 | 11 | 8 | 18 | 60247.870 | 0.099 | 0.050 |
| 19 | 14 | 5 | 19 | | 18 | 13 | 5 | 18 | 60286.360 | 0.218 | 0.050 |
| 19 | 14 | 5 | 20 | | 18 | 13 | 5 | 19 | 60286.360 | 0.123 | 0.050 |
| 19 | 14 | 5 | 18 | | 18 | 13 | 5 | 17 | 60286.360 | 0.122 | 0.050 |
| 19 | 14 | 6 | 18 | | 18 | 13 | 6 | 17 | 60345.530 | -0.034 | 0.050 |
| 19 | 14 | 6 | 19 | | 18 | 13 | 6 | 18 | 60345.530 | 0.061 | 0.050 |
| 19 | 14 | 6 | 20 | | 18 | 13 | 6 | 19 | 60345.530 | -0.032 | 0.050 |
| 18 | 17 | 1 | 19 | | 17 | 16 | 1 | 18 | 60711.510 | 0.002 | 0.050 |
| 18 | 17 | 2 | 19 | | 17 | 16 | 2 | 18 | 60711.510 | 0.002 | 0.050 |
| 18 | 17 | 2 | 17 | | 17 | 16 | 2 | 16 | 60711.510 | 0.008 | 0.050 |
| 18 | 17 | 1 | 17 | | 17 | 16 | 1 | 16 | 60711.510 | 0.008 | 0.050 |
| 18 | 17 | 2 | 18 | | 17 | 16 | 2 | 17 | 60711.510 | 0.044 | 0.050 |
| 18 | 17 | 1 | 18 | | 17 | 16 | 1 | 17 | 60711.510 | 0.044 | 0.050 |
| 18 | 18 | 1 | 19 | | 17 | 17 | 1 | 18 | 61651.800 | 0.010 | 0.050 |
| 18 | 18 | 0 | 17 | | 17 | 17 | 0 | 16 | 61651.800 | 0.020 | 0.050 |
| 18 | 18 | 1 | 18 | | 17 | 17 | 1 | 17 | 61651.800 | 0.028 | 0.050 |
| 18 | 18 | 0 | 18 | | 17 | 17 | 0 | 17 | 61651.800 | 0.028 | 0.050 |
| 18 | 18 | 1 | 17 | | 17 | 17 | 1 | 16 | 61651.800 | 0.020 | 0.050 |
| 18 | 18 | 0 | 19 | | 17 | 17 | 0 | 18 | 61651.800 | 0.010 | 0.050 |
| 20 | 12 | 9 | 19 | | 19 | 11 | 9 | 18 | 61732.170 | -0.067 | 0.050 |
| 20 | 12 | 9 | 20 | | 19 | 11 | 9 | 19 | 61732.170 | 0.037 | 0.050 |
| 20 | 12 | 9 | 21 | | 19 | 11 | 9 | 20 | 61732.170 | -0.063 | 0.050 |
| 20 | 8 | 12 | 19 | | 19 | 7 | 12 | 18 | 62183.820 | 0.096 | 0.050 |

Table S32: Experimental rotational transition frequencies (MHz) for #1-DEAcA *(continued)*

| $J'$ | $K_a'$ | $K_c'$ | $F'$ | - | $J''$ | $K_a''$ | $K_c''$ | $F''$ | $\nu_{\rm obs}$ | $\nu_{\rm obs}-\nu_{\rm calc}$ | Err. |
|---|---|---|---|---|---|---|---|---|---|---|---|
| 20 | 8 | 12 | 21 | | 19 | 7 | 12 | 20 | 62183.820 | 0.107 | 0.050 |
| 20 | 8 | 12 | 20 | | 19 | 7 | 12 | 19 | 62183.820 | 0.293 | 0.050 |
| 20 | 9 | 12 | 21 | | 19 | 8 | 12 | 20 | 62185.720 | -0.270 | 0.050 |
| 20 | 9 | 12 | 19 | | 19 | 8 | 12 | 18 | 62185.720 | -0.281 | 0.050 |
| 20 | 9 | 12 | 20 | | 19 | 8 | 12 | 19 | 62185.720 | -0.084 | 0.050 |
| 20 | 6 | 14 | 19 | | 19 | 5 | 14 | 18 | 62441.660 | -0.027 | 0.050 |
| 20 | 6 | 14 | 21 | | 19 | 5 | 14 | 20 | 62441.660 | -0.011 | 0.050 |
| 20 | 7 | 14 | 21 | | 19 | 6 | 14 | 20 | 62441.660 | -0.017 | 0.050 |
| 20 | 7 | 14 | 19 | | 19 | 6 | 14 | 18 | 62441.660 | -0.033 | 0.050 |
| 20 | 5 | 15 | 21 | | 19 | 4 | 15 | 20 | 62529.290 | -0.038 | 0.050 |
| 20 | 5 | 15 | 19 | | 19 | 4 | 15 | 18 | 62529.290 | -0.058 | 0.050 |
| 20 | 6 | 15 | 19 | | 19 | 5 | 15 | 18 | 62529.290 | -0.058 | 0.050 |
| 20 | 6 | 15 | 21 | | 19 | 5 | 15 | 20 | 62529.290 | -0.038 | 0.050 |
| 20 | 3 | 17 | 19 | | 19 | 2 | 17 | 18 | 62658.630 | 0.054 | 0.050 |
| 20 | 4 | 17 | 21 | | 19 | 3 | 17 | 20 | 62658.630 | 0.081 | 0.050 |
| 20 | 4 | 17 | 19 | | 19 | 3 | 17 | 18 | 62658.630 | 0.054 | 0.050 |
| 20 | 3 | 17 | 21 | | 19 | 2 | 17 | 20 | 62658.630 | 0.081 | 0.050 |
| 19 | 17 | 2 | 19 | | 18 | 16 | 2 | 18 | 63218.610 | 0.048 | 0.050 |
| 19 | 17 | 3 | 19 | | 18 | 16 | 3 | 18 | 63218.610 | 0.040 | 0.050 |
| 19 | 17 | 2 | 18 | | 18 | 16 | 2 | 17 | 63218.610 | -0.005 | 0.050 |
| 19 | 17 | 2 | 20 | | 18 | 16 | 2 | 19 | 63218.610 | -0.009 | 0.050 |
| 19 | 17 | 3 | 20 | | 18 | 16 | 3 | 19 | 63218.610 | -0.018 | 0.050 |
| 19 | 17 | 3 | 18 | | 18 | 16 | 3 | 17 | 63218.610 | -0.014 | 0.050 |
| 19 | 18 | 1 | 19 | | 18 | 17 | 1 | 18 | 64160.520 | 0.068 | 0.050 |
| 19 | 18 | 2 | 19 | | 18 | 17 | 2 | 18 | 64160.520 | 0.068 | 0.050 |
| 19 | 18 | 1 | 18 | | 18 | 17 | 1 | 17 | 64160.520 | 0.036 | 0.050 |
| 19 | 18 | 1 | 20 | | 18 | 17 | 1 | 19 | 64160.520 | 0.030 | 0.050 |
| 19 | 18 | 2 | 20 | | 18 | 17 | 2 | 19 | 64160.520 | 0.030 | 0.050 |
| 19 | 18 | 2 | 18 | | 18 | 17 | 2 | 17 | 64160.520 | 0.036 | 0.050 |
| 21 | 11 | 10 | 20 | | 20 | 10 | 10 | 19 | 64303.880 | -0.011 | 0.050 |
| 21 | 11 | 10 | 21 | | 20 | 10 | 10 | 20 | 64303.880 | 0.076 | 0.050 |
| 21 | 11 | 10 | 22 | | 20 | 10 | 10 | 21 | 64303.880 | -0.008 | 0.050 |
| 20 | 16 | 4 | 20 | | 19 | 15 | 4 | 19 | 64762.990 | 0.089 | 0.050 |
| 20 | 16 | 4 | 21 | | 19 | 15 | 4 | 20 | 64762.990 | 0.011 | 0.050 |
| 20 | 16 | 4 | 19 | | 19 | 15 | 4 | 18 | 64762.990 | 0.011 | 0.050 |
| 20 | 16 | 5 | 20 | | 19 | 15 | 5 | 19 | 64764.720 | 0.008 | 0.050 |
| 20 | 16 | 5 | 19 | | 19 | 15 | 5 | 18 | 64764.720 | -0.071 | 0.050 |
| 20 | 16 | 5 | 21 | | 19 | 15 | 5 | 20 | 64764.720 | -0.071 | 0.050 |
| 21 | 12 | 10 | 20 | | 20 | 11 | 10 | 19 | 64785.150 | -0.042 | 0.050 |
| 21 | 12 | 10 | 21 | | 20 | 11 | 10 | 20 | 64785.150 | 0.066 | 0.050 |
| 21 | 12 | 10 | 22 | | 20 | 11 | 10 | 21 | 64785.150 | -0.038 | 0.050 |
| 21 | 14 | 7 | 22 | | 20 | 13 | 7 | 21 | 64798.990 | -0.095 | 0.050 |
| 21 | 14 | 7 | 21 | | 20 | 13 | 7 | 20 | 64798.990 | -0.008 | 0.050 |
| 21 | 14 | 7 | 20 | | 20 | 13 | 7 | 19 | 64798.990 | -0.097 | 0.050 |
| 21 | 13 | 9 | 20 | | 20 | 12 | 9 | 19 | 64860.730 | -0.062 | 0.050 |
| 21 | 13 | 9 | 21 | | 20 | 12 | 9 | 20 | 64860.730 | 0.032 | 0.050 |
| 21 | 13 | 9 | 22 | | 20 | 12 | 9 | 21 | 64860.730 | -0.059 | 0.050 |
| 21 | 10 | 11 | 22 | | 20 | 9 | 11 | 21 | 64895.690 | -0.089 | 0.050 |
| 21 | 10 | 11 | 21 | | 20 | 9 | 11 | 20 | 64895.690 | 0.035 | 0.050 |
| 21 | 10 | 11 | 20 | | 20 | 9 | 11 | 19 | 64895.690 | -0.095 | 0.050 |
| 21 | 11 | 11 | 22 | | 20 | 10 | 11 | 21 | 64970.180 | -0.097 | 0.050 |
| 21 | 11 | 11 | 20 | | 20 | 10 | 11 | 19 | 64970.180 | -0.103 | 0.050 |
| 21 | 11 | 11 | 21 | | 20 | 10 | 11 | 20 | 64970.180 | 0.031 | 0.050 |
| 19 | 19 | 0 | 19 | | 18 | 18 | 0 | 18 | 65101.770 | -0.009 | 0.050 |
| 19 | 19 | 1 | 19 | | 18 | 18 | 1 | 18 | 65101.770 | -0.009 | 0.050 |
| 19 | 19 | 0 | 18 | | 18 | 18 | 0 | 17 | 65101.770 | -0.017 | 0.050 |
| 19 | 19 | 1 | 18 | | 18 | 18 | 1 | 17 | 65101.770 | -0.017 | 0.050 |

Table S32: Experimental rotational transition frequencies (MHz) for #1-DEAcA *(continued)*

| $J'$ | $K_a'$ | $K_c'$ | $F'$ | - | $J''$ | $K_a''$ | $K_c''$ | $F''$ | $\nu_{\text{obs}}$ | $\nu_{\text{obs}} - \nu_{\text{calc}}$ | Err. |
|---|---|---|---|---|---|---|---|---|---|---|---|
| 19 | 19 | 0 | 20 | | 18 | 18 | 0 | 19 | 65101.770 | -0.025 | 0.050 |
| 19 | 19 | 1 | 20 | | 18 | 18 | 1 | 19 | 65101.770 | -0.025 | 0.050 |
| 21 | 9 | 12 | 22 | | 20 | 8 | 12 | 21 | 65177.480 | 0.038 | 0.050 |
| 21 | 9 | 12 | 20 | | 20 | 8 | 12 | 19 | 65177.480 | 0.030 | 0.050 |
| 21 | 9 | 12 | 21 | | 20 | 8 | 12 | 20 | 65177.480 | 0.196 | 0.050 |
| 21 | 10 | 12 | 22 | | 20 | 9 | 12 | 21 | 65184.730 | -0.047 | 0.050 |
| 21 | 10 | 12 | 20 | | 20 | 9 | 12 | 19 | 65184.730 | -0.055 | 0.050 |
| 21 | 10 | 12 | 21 | | 20 | 9 | 12 | 20 | 65184.730 | 0.111 | 0.050 |
| 21 | 8 | 13 | 22 | | 20 | 7 | 13 | 21 | 65355.640 | 0.219 | 0.050 |
| 21 | 9 | 13 | 20 | | 20 | 8 | 13 | 19 | 65355.640 | -0.307 | 0.050 |
| 21 | 8 | 13 | 20 | | 20 | 7 | 13 | 19 | 65355.640 | 0.208 | 0.050 |
| 21 | 8 | 13 | 21 | | 20 | 7 | 13 | 20 | 65355.640 | 0.409 | 0.050 |
| 21 | 9 | 13 | 22 | | 20 | 8 | 13 | 21 | 65355.640 | -0.297 | 0.050 |
| 21 | 9 | 13 | 21 | | 20 | 8 | 13 | 20 | 65355.640 | -0.106 | 0.050 |
| 21 | 14 | 8 | 21 | | 20 | 13 | 8 | 20 | 65394.120 | 0.115 | 0.050 |
| 21 | 14 | 8 | 20 | | 20 | 13 | 8 | 19 | 65394.120 | 0.025 | 0.050 |
| 21 | 14 | 8 | 22 | | 20 | 13 | 8 | 21 | 65394.120 | 0.027 | 0.050 |
| 21 | 8 | 14 | 22 | | 20 | 7 | 14 | 21 | 65487.020 | -0.030 | 0.050 |
| 21 | 8 | 14 | 20 | | 20 | 7 | 14 | 19 | 65487.020 | -0.043 | 0.050 |
| 21 | 7 | 14 | 22 | | 20 | 6 | 14 | 21 | 65487.020 | -0.003 | 0.050 |
| 21 | 7 | 14 | 20 | | 20 | 6 | 14 | 19 | 65487.020 | -0.016 | 0.050 |
| 21 | 6 | 15 | 21 | | 20 | 5 | 15 | 20 | 65589.740 | 0.143 | 0.050 |
| 21 | 6 | 15 | 22 | | 20 | 5 | 15 | 21 | 65589.740 | -0.118 | 0.050 |
| 21 | 6 | 15 | 20 | | 20 | 5 | 15 | 19 | 65589.740 | -0.133 | 0.050 |
| 21 | 7 | 15 | 20 | | 20 | 6 | 15 | 19 | 65589.740 | -0.134 | 0.050 |
| 21 | 7 | 15 | 22 | | 20 | 6 | 15 | 21 | 65589.740 | -0.119 | 0.050 |
| 21 | 7 | 15 | 21 | | 20 | 6 | 15 | 20 | 65589.740 | 0.142 | 0.050 |
| 21 | 6 | 16 | 20 | | 20 | 5 | 16 | 19 | 65672.520 | -0.056 | 0.050 |
| 21 | 5 | 16 | 20 | | 20 | 4 | 16 | 19 | 65672.520 | -0.056 | 0.050 |
| 21 | 6 | 16 | 22 | | 20 | 5 | 16 | 21 | 65672.520 | -0.038 | 0.050 |
| 21 | 5 | 16 | 22 | | 20 | 4 | 16 | 21 | 65672.520 | -0.038 | 0.050 |
| 21 | 6 | 16 | 21 | | 20 | 5 | 16 | 20 | 65672.520 | 0.262 | 0.050 |
| 21 | 5 | 16 | 21 | | 20 | 4 | 16 | 20 | 65672.520 | 0.262 | 0.050 |
| 20 | 17 | 3 | 20 | | 19 | 16 | 3 | 19 | 65720.640 | 0.084 | 0.050 |
| 20 | 17 | 3 | 19 | | 19 | 16 | 3 | 18 | 65720.640 | 0.018 | 0.050 |
| 20 | 17 | 3 | 21 | | 19 | 16 | 3 | 20 | 65720.640 | 0.017 | 0.050 |
| 20 | 17 | 4 | 20 | | 19 | 16 | 4 | 19 | 65720.640 | -0.002 | 0.050 |
| 20 | 17 | 4 | 19 | | 19 | 16 | 4 | 18 | 65720.640 | -0.068 | 0.050 |
| 20 | 17 | 4 | 21 | | 19 | 16 | 4 | 20 | 65720.640 | -0.070 | 0.050 |
| 21 | 5 | 17 | 20 | | 20 | 4 | 17 | 19 | 65740.400 | -0.072 | 0.050 |
| 21 | 4 | 17 | 20 | | 20 | 3 | 17 | 19 | 65740.400 | -0.072 | 0.050 |
| 21 | 5 | 17 | 22 | | 20 | 4 | 17 | 21 | 65740.400 | -0.051 | 0.050 |
| 21 | 4 | 17 | 22 | | 20 | 3 | 17 | 21 | 65740.400 | -0.051 | 0.050 |
| 21 | 3 | 18 | 21 | | 20 | 2 | 18 | 20 | 65796.700 | -0.029 | 0.050 |
| 21 | 4 | 18 | 21 | | 20 | 3 | 18 | 20 | 65796.700 | -0.029 | 0.050 |
| 21 | 3 | 18 | 22 | | 20 | 2 | 18 | 21 | 65797.140 | 0.029 | 0.050 |
| 21 | 4 | 18 | 22 | | 20 | 3 | 18 | 21 | 65797.140 | 0.029 | 0.050 |
| 21 | 3 | 18 | 20 | | 20 | 2 | 18 | 19 | 65797.140 | 0.004 | 0.050 |
| 21 | 4 | 18 | 20 | | 20 | 3 | 18 | 19 | 65797.140 | 0.004 | 0.050 |
| 21 | 3 | 19 | 21 | | 20 | 2 | 19 | 20 | 65844.650 | 0.027 | 0.050 |
| 21 | 2 | 19 | 21 | | 20 | 1 | 19 | 20 | 65844.650 | 0.027 | 0.050 |
| 21 | 3 | 19 | 20 | | 20 | 2 | 19 | 19 | 65844.970 | -0.107 | 0.050 |
| 21 | 3 | 19 | 22 | | 20 | 2 | 19 | 21 | 65844.970 | -0.080 | 0.050 |
| 21 | 2 | 19 | 20 | | 20 | 1 | 19 | 19 | 65844.970 | -0.107 | 0.050 |
| 21 | 2 | 19 | 22 | | 20 | 1 | 19 | 21 | 65844.970 | -0.080 | 0.050 |
| 21 | 1 | 20 | 22 | | 20 | 0 | 20 | 21 | 65886.130 | 0.040 | 0.050 |
| 21 | 2 | 20 | 20 | | 20 | 1 | 20 | 19 | 65886.130 | 0.009 | 0.050 |

Table S32: Experimental rotational transition frequencies (MHz) for #1-DEAcA *(continued)*

| $J'$ | $K_a'$ | $K_c'$ | $F'$ | - | $J''$ | $K_a''$ | $K_c''$ | $F''$ | $\nu_{\text{obs}}$ | $\nu_{\text{obs}} - \nu_{\text{calc}}$ | Err. |
|---|---|---|---|---|---|---|---|---|---|---|---|
| 21 | 2 | 20 | 22 | | 20 | 1 | 20 | 21 | 65886.130 | 0.040 | 0.050 |
| 21 | 1 | 20 | 20 | | 20 | 0 | 20 | 19 | 65886.130 | 0.009 | 0.050 |
| 21 | 15 | 6 | 22 | | 20 | 14 | 6 | 21 | 66167.270 | -0.085 | 0.050 |
| 21 | 15 | 6 | 21 | | 20 | 14 | 6 | 20 | 66167.270 | 0.002 | 0.050 |
| 21 | 15 | 6 | 20 | | 20 | 14 | 6 | 19 | 66167.270 | -0.087 | 0.050 |

Table S33: Experimental rotational transition frequencies (MHz) for #1-DEAcA($^{13}$C1, aaAs).

| $J'$ | $K_a'$ | $K_c'$ | $F'$ | - | $J''$ | $K_a''$ | $K_c''$ | $F''$ | $\nu_{\mathrm{obs}}$ | $\nu_{\mathrm{obs}} - \nu_{\mathrm{calc}}$ | Err. |
|---|---|---|---|---|---|---|---|---|---|---|---|
| 1 | 1 | 0 | 2 | | 0 | 0 | 0 | 1 | 3133.155 | 0.005 | 0.010 |
| 2 | 1 | 1 | 2 | | 1 | 0 | 1 | 1 | 6015.291 | 0.014 | 0.010 |
| 2 | 1 | 1 | 1 | | 1 | 0 | 1 | 1 | 6015.571 | -0.009 | 0.010 |
| 2 | 1 | 1 | 2 | | 1 | 0 | 1 | 2 | 6016.671 | 0.001 | 0.010 |
| 2 | 1 | 1 | 3 | | 1 | 0 | 1 | 2 | 6016.864 | -0.000 | 0.010 |
| 2 | 1 | 1 | 1 | | 1 | 0 | 1 | 0 | 6019.062 | -0.001 | 0.010 |
| 3 | 0 | 3 | 4 | | 2 | 0 | 2 | 3 | 6289.786 | 0.000 | 0.010 |
| 3 | 0 | 3 | 3 | | 2 | 0 | 2 | 2 | 6290.195 | 0.000 | 0.010 |
| 2 | 2 | 0 | 2 | | 1 | 1 | 0 | 2 | 6336.440 | -0.013 | 0.010 |
| 2 | 2 | 0 | 1 | | 1 | 1 | 0 | 0 | 6336.950 | -0.002 | 0.010 |
| 2 | 2 | 0 | 2 | | 1 | 1 | 0 | 1 | 6338.030 | 0.003 | 0.010 |
| 2 | 2 | 0 | 3 | | 1 | 1 | 0 | 2 | 6338.290 | -0.002 | 0.010 |
| 2 | 2 | 0 | 1 | | 1 | 1 | 0 | 1 | 6340.870 | -0.019 | 0.010 |
| 2 | 2 | 1 | 2 | | 1 | 1 | 1 | 1 | 6513.664 | 0.013 | 0.010 |
| 2 | 2 | 1 | 2 | | 1 | 1 | 1 | 2 | 6513.815 | -0.017 | 0.010 |
| 2 | 2 | 1 | 3 | | 1 | 1 | 1 | 2 | 6515.327 | 0.003 | 0.010 |
| 2 | 2 | 1 | 1 | | 1 | 1 | 1 | 1 | 6515.969 | -0.004 | 0.010 |
| 2 | 2 | 1 | 1 | | 1 | 1 | 1 | 0 | 6516.454 | 0.028 | 0.010 |

Table S34: Experimental rotational transition frequencies (MHz) for #1-DEAcA($^{13}$C2, aaAs).

| $J'$ | $K'_a$ | $K'_c$ | $F'$ | - | $J''$ | $K''_a$ | $K''_c$ | $F''$ | $\nu_{\text{obs}}$ | $\nu_{\text{obs}} - \nu_{\text{calc}}$ | Err. |
|---|---|---|---|---|---|---|---|---|---|---|---|
| 1 | 1 | 0 | 2 | | 0 | 0 | 0 | 1 | 3151.970 | -0.001 | 0.010 |
| 2 | 1 | 1 | 2 | | 1 | 0 | 1 | 1 | 6019.341 | 0.004 | 0.010 |
| 2 | 1 | 1 | 1 | | 1 | 0 | 1 | 1 | 6019.629 | 0.008 | 0.010 |
| 2 | 1 | 1 | 2 | | 1 | 0 | 1 | 2 | 6020.723 | -0.002 | 0.010 |
| 2 | 1 | 1 | 3 | | 1 | 0 | 1 | 2 | 6020.904 | -0.003 | 0.010 |
| 2 | 1 | 1 | 1 | | 1 | 0 | 1 | 0 | 6023.084 | -0.006 | 0.010 |
| 3 | 0 | 3 | 4 | | 2 | 0 | 2 | 3 | 6327.051 | 0.000 | 0.010 |
| 3 | 0 | 3 | 2 | | 2 | 0 | 2 | 1 | 6327.292 | 0.005 | 0.010 |
| 3 | 0 | 3 | 3 | | 2 | 0 | 2 | 2 | 6327.445 | -0.006 | 0.010 |
| 2 | 2 | 0 | 2 | | 1 | 1 | 0 | 2 | 6393.620 | 0.011 | 0.010 |
| 2 | 2 | 0 | 1 | | 1 | 1 | 0 | 0 | 6394.110 | -0.007 | 0.010 |
| 2 | 2 | 0 | 2 | | 1 | 1 | 0 | 1 | 6395.160 | -0.006 | 0.010 |
| 2 | 2 | 0 | 3 | | 1 | 1 | 0 | 2 | 6395.430 | -0.007 | 0.010 |
| 2 | 2 | 0 | 1 | | 1 | 1 | 0 | 1 | 6398.020 | 0.009 | 0.010 |
| 2 | 2 | 1 | 2 | | 1 | 1 | 1 | 1 | 6584.910 | -0.002 | 0.010 |
| 2 | 2 | 1 | 2 | | 1 | 1 | 1 | 2 | 6585.076 | -0.005 | 0.010 |
| 2 | 2 | 1 | 3 | | 1 | 1 | 1 | 2 | 6586.565 | -0.003 | 0.010 |
| 2 | 2 | 1 | 1 | | 1 | 1 | 1 | 1 | 6587.235 | 0.009 | 0.010 |
| 2 | 2 | 1 | 1 | | 1 | 1 | 1 | 0 | 6587.650 | 0.001 | 0.010 |

Table S35: Experimental rotational transition frequencies (MHz) for #1'-DEAcA (aaAa).

| $J'$ | $K'_a$ | $K'_c$ | $F'$ | - | $J''$ | $K''_a$ | $K''_c$ | $F''$ | $\nu_{\text{obs}}$ | $\nu_{\text{obs}} - \nu_{\text{calc}}$ | Err. |
|---|---|---|---|---|---|---|---|---|---|---|---|
| 1 | 0 | 1 | 0 | | 0 | 0 | 0 | 1 | 2428.067 | -0.004 | 0.010 |
| 1 | 0 | 1 | 2 | | 0 | 0 | 0 | 1 | 2429.771 | 0.019 | 0.010 |
| 1 | 0 | 1 | 1 | | 0 | 0 | 0 | 1 | 2430.892 | 0.019 | 0.010 |
| 1 | 1 | 0 | 1 | | 0 | 0 | 0 | 1 | 3145.349 | 0.018 | 0.010 |
| 1 | 1 | 0 | 2 | | 0 | 0 | 0 | 1 | 3146.648 | 0.040 | 0.010 |
| 1 | 1 | 0 | 0 | | 0 | 0 | 0 | 1 | 3148.546 | 0.021 | 0.010 |
| 2 | 1 | 2 | 3 | | 1 | 1 | 1 | 2 | 4362.357 | -0.017 | 0.010 |
| 2 | 1 | 1 | 1 | | 1 | 1 | 0 | 0 | 5355.139 | 0.036 | 0.010 |
| 2 | 1 | 1 | 2 | | 1 | 1 | 0 | 2 | 5356.793 | 0.035 | 0.010 |
| 2 | 1 | 1 | 3 | | 1 | 1 | 0 | 2 | 5356.969 | 0.043 | 0.010 |
| 2 | 1 | 1 | 2 | | 1 | 1 | 0 | 1 | 5358.064 | 0.028 | 0.010 |
| 2 | 1 | 1 | 1 | | 1 | 1 | 0 | 1 | 5358.338 | 0.040 | 0.010 |
| 2 | 1 | 1 | 2 | | 1 | 0 | 1 | 1 | 6072.560 | 0.066 | 0.010 |
| 2 | 1 | 1 | 2 | | 1 | 0 | 1 | 2 | 6073.680 | 0.066 | 0.010 |
| 2 | 1 | 1 | 3 | | 1 | 0 | 1 | 2 | 6073.860 | 0.077 | 0.010 |
| 2 | 1 | 1 | 1 | | 1 | 0 | 1 | 0 | 6075.643 | 0.085 | 0.010 |
| 2 | 2 | 0 | 1 | | 1 | 1 | 0 | 0 | 6349.821 | -0.009 | 0.010 |
| 2 | 2 | 0 | 2 | | 1 | 1 | 0 | 1 | 6350.708 | -0.007 | 0.010 |
| 2 | 2 | 0 | 3 | | 1 | 1 | 0 | 2 | 6350.917 | -0.005 | 0.010 |
| 2 | 2 | 0 | 1 | | 1 | 1 | 0 | 1 | 6353.015 | -0.010 | 0.010 |
| 3 | 1 | 3 | 4 | | 2 | 1 | 2 | 3 | 6372.332 | 0.002 | 0.005 |
| 3 | 1 | 3 | 2 | | 2 | 1 | 2 | 1 | 6372.473 | 0.015 | 0.005 |
| 3 | 1 | 3 | 3 | | 2 | 1 | 2 | 2 | 6372.776 | 0.007 | 0.005 |
| 3 | 0 | 3 | 2 | | 2 | 0 | 2 | 2 | 6418.247 | 0.010 | 0.005 |
| 3 | 0 | 3 | 4 | | 2 | 0 | 2 | 3 | 6420.356 | -0.003 | 0.005 |
| 3 | 0 | 3 | 2 | | 2 | 0 | 2 | 1 | 6420.532 | -0.015 | 0.005 |
| 3 | 0 | 3 | 3 | | 2 | 0 | 2 | 2 | 6420.692 | -0.003 | 0.005 |
| 3 | 0 | 3 | 3 | | 2 | 0 | 2 | 3 | 6422.166 | -0.014 | 0.005 |
| 2 | 2 | 1 | 2 | | 1 | 1 | 1 | 1 | 6510.985 | 0.027 | 0.010 |
| 2 | 2 | 1 | 3 | | 1 | 1 | 1 | 2 | 6512.356 | 0.040 | 0.010 |
| 2 | 2 | 1 | 1 | | 1 | 1 | 1 | 0 | 6513.250 | 0.031 | 0.010 |
| 3 | 2 | 2 | 2 | | 2 | 2 | 1 | 1 | 7288.879 | -0.003 | 0.005 |
| 3 | 2 | 2 | 4 | | 2 | 2 | 1 | 3 | 7289.535 | -0.013 | 0.005 |
| 3 | 2 | 2 | 3 | | 2 | 2 | 1 | 2 | 7290.739 | -0.011 | 0.005 |
| 3 | 1 | 2 | 2 | | 2 | 1 | 1 | 1 | 7679.671 | -0.027 | 0.005 |
| 3 | 1 | 2 | 4 | | 2 | 1 | 1 | 3 | 7679.915 | 0.030 | 0.005 |
| 3 | 1 | 2 | 3 | | 2 | 1 | 1 | 2 | 7680.345 | 0.023 | 0.005 |
| 3 | 2 | 2 | 3 | | 2 | 1 | 2 | 2 | 9438.096 | -0.025 | 0.005 |
| 3 | 2 | 2 | 2 | | 2 | 1 | 2 | 1 | 9440.220 | -0.031 | 0.005 |
| 3 | 3 | 1 | 3 | | 2 | 2 | 1 | 2 | 9800.261 | 0.013 | 0.005 |
| 3 | 3 | 1 | 2 | | 2 | 2 | 1 | 1 | 9800.603 | 0.018 | 0.005 |
| 3 | 3 | 1 | 4 | | 2 | 2 | 1 | 3 | 9800.693 | 0.013 | 0.005 |
| 4 | 3 | 1 | 4 | | 3 | 2 | 1 | 3 | 12296.042 | -0.029 | 0.005 |
| 4 | 3 | 1 | 5 | | 3 | 2 | 1 | 4 | 12296.281 | -0.029 | 0.005 |
| 4 | 3 | 1 | 3 | | 3 | 2 | 1 | 2 | 12296.281 | -0.037 | 0.005 |

Table S36: Experimental rotational transition frequencies (MHz) for #2-DEAcA (gaAs).

| $J'$ | $K_a'$ | $K_c'$ | $F'$ | - | $J''$ | $K_a''$ | $K_c''$ | $F''$ | $\nu_{\text{obs}}$ | $\nu_{\text{obs}} - \nu_{\text{calc}}$ | Err. |
|---|---|---|---|---|---|---|---|---|---|---|---|
| 3 | 1 | 2 | 3 | | 3 | 0 | 3 | 3 | 2278.692 | 0.017 | 0.010 |
| 3 | 1 | 2 | 4 | | 3 | 0 | 3 | 4 | 2280.735 | 0.010 | 0.010 |
| 3 | 1 | 2 | 2 | | 3 | 0 | 3 | 2 | 2281.455 | 0.011 | 0.010 |
| 3 | 2 | 1 | 3 | | 3 | 1 | 2 | 3 | 2450.968 | 0.001 | 0.010 |
| 3 | 2 | 1 | 4 | | 3 | 1 | 2 | 4 | 2451.959 | 0.008 | 0.010 |
| 3 | 2 | 1 | 2 | | 3 | 1 | 2 | 2 | 2452.305 | 0.010 | 0.010 |
| 2 | 2 | 1 | 2 | | 2 | 1 | 1 | 3 | 2520.553 | -0.002 | 0.010 |
| 2 | 2 | 1 | 2 | | 2 | 1 | 1 | 2 | 2520.729 | -0.002 | 0.010 |
| 2 | 2 | 1 | 3 | | 2 | 1 | 1 | 3 | 2522.290 | -0.015 | 0.010 |
| 2 | 2 | 1 | 3 | | 2 | 1 | 1 | 2 | 2522.474 | -0.008 | 0.010 |
| 2 | 2 | 1 | 1 | | 2 | 1 | 1 | 1 | 2523.177 | -0.001 | 0.010 |
| 4 | 2 | 2 | 4 | | 4 | 1 | 3 | 4 | 2532.892 | 0.007 | 0.010 |
| 4 | 2 | 2 | 5 | | 4 | 1 | 3 | 5 | 2533.747 | -0.010 | 0.010 |
| 4 | 2 | 2 | 3 | | 4 | 1 | 3 | 3 | 2533.977 | -0.004 | 0.010 |
| 2 | 2 | 0 | 2 | | 2 | 1 | 1 | 2 | 2597.623 | -0.009 | 0.010 |
| 2 | 2 | 0 | 3 | | 2 | 1 | 1 | 3 | 2599.452 | -0.016 | 0.010 |
| 2 | 2 | 0 | 1 | | 2 | 1 | 1 | 1 | 2600.474 | -0.013 | 0.010 |
| 2 | 0 | 2 | 1 | | 1 | 1 | 0 | 0 | 2834.846 | -0.024 | 0.010 |
| 2 | 0 | 2 | 3 | | 1 | 1 | 0 | 2 | 2838.685 | -0.002 | 0.010 |
| 2 | 0 | 2 | 2 | | 1 | 1 | 0 | 2 | 2840.696 | -0.004 | 0.010 |
| 2 | 0 | 2 | 2 | | 1 | 1 | 0 | 1 | 2842.488 | -0.011 | 0.010 |
| 1 | 1 | 1 | 0 | | 0 | 0 | 0 | 1 | 2881.045 | -0.009 | 0.010 |
| 1 | 1 | 1 | 2 | | 0 | 0 | 0 | 1 | 2881.298 | -0.004 | 0.010 |
| 1 | 1 | 1 | 1 | | 0 | 0 | 0 | 1 | 2881.459 | -0.007 | 0.010 |
| 2 | 0 | 2 | 1 | | 1 | 1 | 1 | 0 | 3162.291 | 0.005 | 0.010 |
| 2 | 0 | 2 | 3 | | 1 | 1 | 1 | 2 | 3163.160 | 0.004 | 0.010 |
| 2 | 0 | 2 | 2 | | 1 | 1 | 1 | 1 | 3165.015 | 0.010 | 0.010 |
| 1 | 1 | 0 | 1 | | 0 | 0 | 0 | 1 | 3203.970 | -0.002 | 0.010 |
| 1 | 1 | 0 | 2 | | 0 | 0 | 0 | 1 | 3205.767 | -0.004 | 0.010 |
| 1 | 1 | 0 | 0 | | 0 | 0 | 0 | 1 | 3208.470 | -0.000 | 0.010 |
| 4 | 1 | 3 | 4 | | 4 | 0 | 4 | 4 | 3332.675 | 0.019 | 0.010 |
| 4 | 1 | 3 | 5 | | 4 | 0 | 4 | 5 | 3334.704 | 0.012 | 0.010 |
| 4 | 1 | 3 | 3 | | 4 | 0 | 4 | 3 | 3335.205 | -0.011 | 0.010 |
| 2 | 2 | 1 | 2 | | 2 | 1 | 2 | 2 | 3491.528 | 0.008 | 0.010 |
| 2 | 2 | 1 | 3 | | 2 | 1 | 2 | 3 | 3495.231 | 0.033 | 0.010 |
| 2 | 2 | 1 | 1 | | 2 | 1 | 2 | 1 | 3497.262 | 0.021 | 0.010 |
| 2 | 2 | 0 | 2 | | 2 | 1 | 2 | 2 | 3568.422 | 0.002 | 0.010 |
| 2 | 2 | 0 | 3 | | 2 | 1 | 2 | 3 | 3572.356 | -0.005 | 0.010 |
| 2 | 2 | 0 | 1 | | 2 | 1 | 2 | 1 | 3574.552 | 0.002 | 0.010 |
| 2 | 1 | 2 | 1 | | 1 | 1 | 1 | 0 | 3755.970 | 0.002 | 0.010 |
| 2 | 1 | 2 | 3 | | 1 | 1 | 1 | 2 | 3756.805 | 0.015 | 0.010 |
| 2 | 1 | 2 | 2 | | 1 | 1 | 1 | 1 | 3758.537 | -0.016 | 0.010 |
| 2 | 1 | 2 | 2 | | 1 | 1 | 1 | 2 | 3758.716 | -0.002 | 0.010 |
| 3 | 1 | 2 | 2 | | 2 | 2 | 0 | 1 | 3951.899 | 0.009 | 0.010 |
| 3 | 1 | 2 | 4 | | 2 | 2 | 0 | 3 | 3953.157 | 0.019 | 0.010 |
| 3 | 1 | 2 | 3 | | 2 | 2 | 0 | 2 | 3955.544 | 0.018 | 0.010 |
| 2 | 0 | 2 | 3 | | 1 | 0 | 1 | 2 | 4004.074 | 0.016 | 0.010 |
| 2 | 0 | 2 | 2 | | 1 | 0 | 1 | 1 | 4004.443 | 0.005 | 0.010 |
| 3 | 2 | 2 | 3 | | 3 | 1 | 3 | 3 | 4022.283 | 0.015 | 0.010 |
| 3 | 2 | 2 | 4 | | 3 | 1 | 3 | 4 | 4024.791 | -0.004 | 0.010 |
| 3 | 2 | 2 | 2 | | 3 | 1 | 3 | 2 | 4025.669 | -0.011 | 0.010 |
| 3 | 0 | 3 | 2 | | 2 | 1 | 1 | 1 | 4270.931 | -0.002 | 0.010 |
| 3 | 0 | 3 | 2 | | 2 | 1 | 1 | 2 | 4271.189 | -0.021 | 0.010 |
| 3 | 0 | 3 | 4 | | 2 | 1 | 1 | 3 | 4271.877 | -0.005 | 0.010 |
| 3 | 0 | 3 | 3 | | 2 | 1 | 1 | 3 | 4274.309 | 0.003 | 0.010 |
| 3 | 0 | 3 | 3 | | 2 | 1 | 1 | 2 | 4274.485 | 0.002 | 0.010 |
| 4 | 3 | 2 | 4 | | 4 | 2 | 2 | 4 | 4284.417 | 0.015 | 0.010 |

Table S36: Experimental rotational transition frequencies (MHz) for #2-DEAcA. *(continued)*

| $J'$ | $K_a'$ | $K_c'$ | $F'$ | - | $J''$ | $K_a''$ | $K_c''$ | $F''$ | $\nu_{\text{obs}}$ | $\nu_{\text{obs}} - \nu_{\text{calc}}$ | Err. |
|---|---|---|---|---|---|---|---|---|---|---|---|
| 4 | 3 | 2 | 5 | | 4 | 2 | 2 | 5 | 4285.124 | 0.020 | 0.010 |
| 4 | 3 | 1 | 5 | | 4 | 2 | 2 | 5 | 4365.352 | 0.033 | 0.010 |
| 4 | 3 | 1 | 3 | | 4 | 2 | 2 | 3 | 4365.552 | 0.022 | 0.010 |
| 3 | 2 | 1 | 3 | | 3 | 1 | 3 | 3 | 4382.368 | 0.027 | 0.010 |
| 3 | 2 | 1 | 4 | | 3 | 1 | 3 | 4 | 4385.488 | 0.011 | 0.010 |
| 3 | 2 | 1 | 2 | | 3 | 1 | 3 | 2 | 4386.588 | 0.012 | 0.010 |
| 2 | 1 | 1 | 1 | | 1 | 1 | 0 | 0 | 4402.636 | 0.021 | 0.010 |
| 2 | 1 | 1 | 2 | | 1 | 1 | 0 | 2 | 4405.027 | -0.010 | 0.010 |
| 2 | 1 | 1 | 3 | | 1 | 1 | 0 | 2 | 4405.203 | -0.010 | 0.010 |
| 2 | 1 | 1 | 2 | | 1 | 1 | 0 | 1 | 4406.832 | -0.004 | 0.010 |
| 2 | 1 | 1 | 1 | | 1 | 1 | 0 | 1 | 4407.122 | 0.009 | 0.010 |
| 2 | 1 | 2 | 1 | | 1 | 0 | 1 | 1 | 4594.979 | -0.009 | 0.010 |
| 2 | 1 | 2 | 3 | | 1 | 0 | 1 | 2 | 4597.681 | -0.011 | 0.010 |
| 2 | 1 | 2 | 2 | | 1 | 0 | 1 | 1 | 4597.974 | -0.012 | 0.010 |
| 2 | 1 | 2 | 1 | | 1 | 0 | 1 | 0 | 4599.062 | -0.010 | 0.010 |
| 2 | 1 | 2 | 2 | | 1 | 0 | 1 | 2 | 4599.607 | -0.013 | 0.010 |
| 3 | 3 | 1 | 3 | | 3 | 2 | 1 | 3 | 4695.338 | 0.002 | 0.010 |
| 3 | 3 | 1 | 4 | | 3 | 2 | 1 | 4 | 4697.071 | -0.000 | 0.010 |
| 3 | 3 | 1 | 4 | | 3 | 2 | 1 | 3 | 4697.677 | -0.003 | 0.010 |
| 3 | 3 | 0 | 3 | | 3 | 2 | 1 | 3 | 4707.163 | -0.012 | 0.010 |
| 3 | 3 | 0 | 4 | | 3 | 2 | 1 | 4 | 4708.936 | -0.003 | 0.010 |
| 3 | 3 | 0 | 2 | | 3 | 2 | 1 | 2 | 4709.557 | 0.001 | 0.010 |
| 4 | 2 | 3 | 4 | | 4 | 1 | 4 | 4 | 4735.178 | 0.016 | 0.010 |
| 4 | 2 | 3 | 5 | | 4 | 1 | 4 | 5 | 4737.243 | 0.024 | 0.010 |
| 4 | 2 | 3 | 3 | | 4 | 1 | 4 | 3 | 4737.775 | 0.027 | 0.010 |
| 3 | 3 | 1 | 3 | | 3 | 2 | 2 | 3 | 5055.415 | 0.006 | 0.010 |
| 3 | 3 | 1 | 4 | | 3 | 2 | 2 | 4 | 5057.739 | -0.014 | 0.010 |
| 3 | 3 | 1 | 2 | | 3 | 2 | 2 | 2 | 5058.563 | -0.010 | 0.010 |
| 3 | 3 | 0 | 3 | | 3 | 2 | 2 | 3 | 5067.254 | 0.006 | 0.010 |
| 3 | 3 | 0 | 4 | | 3 | 2 | 2 | 4 | 5069.613 | -0.009 | 0.010 |
| 3 | 3 | 0 | 2 | | 3 | 2 | 2 | 2 | 5070.441 | -0.010 | 0.010 |
| 3 | 0 | 3 | 4 | | 2 | 1 | 2 | 3 | 5244.783 | 0.009 | 0.010 |
| 3 | 0 | 3 | 2 | | 2 | 1 | 2 | 1 | 5245.012 | 0.016 | 0.010 |
| 3 | 0 | 3 | 3 | | 2 | 1 | 2 | 2 | 5245.282 | 0.011 | 0.010 |
| 4 | 0 | 4 | 3 | | 3 | 1 | 2 | 2 | 5282.120 | 0.002 | 0.010 |
| 4 | 0 | 4 | 5 | | 3 | 1 | 2 | 4 | 5282.700 | -0.004 | 0.010 |
| 4 | 0 | 4 | 4 | | 3 | 1 | 2 | 3 | 5285.116 | -0.003 | 0.010 |
| 4 | 3 | 1 | 4 | | 4 | 2 | 3 | 4 | 5321.916 | 0.012 | 0.010 |
| 4 | 3 | 1 | 5 | | 4 | 2 | 3 | 5 | 5323.677 | 0.024 | 0.010 |
| 4 | 3 | 1 | 3 | | 4 | 2 | 3 | 3 | 5324.114 | 0.011 | 0.010 |
| 2 | 1 | 1 | 2 | | 1 | 0 | 1 | 1 | 5568.776 | 0.002 | 0.010 |
| 2 | 1 | 1 | 1 | | 1 | 0 | 1 | 1 | 5569.054 | 0.003 | 0.010 |
| 2 | 1 | 1 | 2 | | 1 | 0 | 1 | 2 | 5570.410 | 0.002 | 0.010 |
| 2 | 1 | 1 | 3 | | 1 | 0 | 1 | 2 | 5570.586 | 0.001 | 0.010 |
| 2 | 1 | 1 | 1 | | 1 | 0 | 1 | 0 | 5573.134 | -0.001 | 0.010 |
| 3 | 1 | 3 | 4 | | 2 | 1 | 2 | 3 | 5591.985 | 0.013 | 0.010 |
| 3 | 1 | 3 | 3 | | 2 | 1 | 2 | 2 | 5592.577 | 0.004 | 0.010 |
| 4 | 2 | 2 | 4 | | 4 | 1 | 4 | 4 | 5692.539 | -0.028 | 0.010 |
| 4 | 2 | 2 | 5 | | 4 | 1 | 4 | 5 | 5695.537 | -0.015 | 0.010 |
| 4 | 2 | 2 | 3 | | 4 | 1 | 4 | 3 | 5696.312 | -0.009 | 0.010 |
| 3 | 0 | 3 | 4 | | 2 | 0 | 2 | 3 | 5838.410 | 0.002 | 0.010 |
| 3 | 0 | 3 | 2 | | 2 | 0 | 2 | 1 | 5838.666 | -0.011 | 0.010 |
| 3 | 0 | 3 | 3 | | 2 | 0 | 2 | 2 | 5838.820 | 0.001 | 0.010 |
| 5 | 3 | 2 | 5 | | 5 | 2 | 4 | 5 | 5875.984 | -0.049 | 0.010 |
| 5 | 3 | 2 | 6 | | 5 | 2 | 4 | 6 | 5877.613 | -0.034 | 0.010 |
| 5 | 3 | 2 | 4 | | 5 | 2 | 4 | 4 | 5877.933 | -0.043 | 0.010 |
| 3 | 2 | 2 | 2 | | 2 | 2 | 1 | 1 | 6120.618 | 0.020 | 0.010 |

Table S36: Experimental rotational transition frequencies (MHz) for #2-DEAcA. *(continued)*

| $J'$ | $K_a'$ | $K_c'$ | $F'$ | - | $J''$ | $K_a''$ | $K_c''$ | $F''$ | $\nu_{\text{obs}}$ | $\nu_{\text{obs}} - \nu_{\text{calc}}$ | Err. |
|---|---|---|---|---|---|---|---|---|---|---|---|
| 3 | 2 | 2 | 3 | | 2 | 2 | 1 | 2 | 6123.337 | 0.016 | 0.010 |
| 4 | 1 | 3 | 5 | | 3 | 2 | 1 | 4 | 6165.437 | -0.009 | 0.010 |
| 4 | 1 | 3 | 4 | | 3 | 2 | 1 | 3 | 6166.805 | -0.002 | 0.010 |
| 3 | 1 | 3 | 4 | | 2 | 0 | 2 | 3 | 6185.606 | -0.000 | 0.010 |
| 3 | 1 | 3 | 2 | | 2 | 0 | 2 | 1 | 6185.835 | -0.005 | 0.010 |
| 3 | 1 | 3 | 3 | | 2 | 0 | 2 | 2 | 6186.116 | -0.005 | 0.010 |
| 3 | 2 | 1 | 4 | | 2 | 2 | 0 | 3 | 6405.100 | 0.011 | 0.010 |
| 4 | 1 | 3 | 3 | | 3 | 2 | 2 | 2 | 6525.924 | -0.010 | 0.010 |
| 4 | 1 | 3 | 5 | | 3 | 2 | 2 | 4 | 6526.123 | -0.005 | 0.010 |
| 4 | 1 | 3 | 4 | | 3 | 2 | 2 | 3 | 6526.874 | -0.006 | 0.010 |
| 3 | 1 | 2 | 2 | | 2 | 1 | 1 | 1 | 6552.376 | -0.000 | 0.010 |
| 3 | 1 | 2 | 4 | | 2 | 1 | 1 | 3 | 6552.607 | 0.000 | 0.010 |
| 3 | 1 | 2 | 3 | | 2 | 1 | 1 | 2 | 6553.169 | 0.011 | 0.010 |
| 2 | 2 | 0 | 2 | | 1 | 1 | 0 | 2 | 7002.659 | -0.009 | 0.010 |
| 2 | 2 | 0 | 1 | | 1 | 1 | 0 | 0 | 7003.096 | -0.006 | 0.010 |
| 2 | 2 | 0 | 2 | | 1 | 1 | 0 | 1 | 7004.461 | -0.006 | 0.010 |
| 2 | 2 | 0 | 3 | | 1 | 1 | 0 | 2 | 7004.677 | -0.005 | 0.010 |
| 2 | 2 | 1 | 2 | | 1 | 1 | 1 | 1 | 7250.067 | -0.006 | 0.010 |
| 2 | 2 | 1 | 2 | | 1 | 1 | 1 | 2 | 7250.238 | 0.001 | 0.010 |
| 2 | 2 | 1 | 3 | | 1 | 1 | 1 | 2 | 7251.986 | -0.002 | 0.010 |
| 2 | 2 | 1 | 1 | | 1 | 1 | 1 | 1 | 7252.800 | 0.003 | 0.010 |
| 2 | 2 | 1 | 1 | | 1 | 1 | 1 | 0 | 7253.201 | -0.007 | 0.010 |
| 2 | 2 | 0 | 2 | | 1 | 1 | 1 | 1 | 7326.970 | -0.003 | 0.010 |
| 2 | 2 | 0 | 2 | | 1 | 1 | 1 | 2 | 7327.138 | 0.000 | 0.010 |
| 2 | 2 | 0 | 3 | | 1 | 1 | 1 | 2 | 7329.151 | 0.000 | 0.010 |
| 2 | 2 | 0 | 1 | | 1 | 1 | 1 | 1 | 7330.111 | 0.005 | 0.010 |
| 2 | 2 | 0 | 1 | | 1 | 1 | 1 | 0 | 7330.514 | -0.003 | 0.010 |

Table S37: Experimental rotational transition frequencies (MHz) for #3-DEAcA (agAs).

| $J'$ | $K'_a$ | $K'_c$ | $F'$ | - | $J''$ | $K''_a$ | $K''_c$ | $F''$ | $\nu_{\mathrm{obs}}$ | $\nu_{\mathrm{obs}} - \nu_{\mathrm{calc}}$ | Err. |
|---|---|---|---|---|---|---|---|---|---|---|---|
| 1 | 1 | 0 | 1 | | 0 | 0 | 0 | 1 | 3134.025 | 0.004 | 0.010 |
| 1 | 1 | 0 | 2 | | 0 | 0 | 0 | 1 | 3134.951 | 0.011 | 0.010 |
| 1 | 1 | 0 | 0 | | 0 | 0 | 0 | 1 | 3136.344 | -0.001 | 0.010 |
| 3 | 3 | 1 | 4 | | 3 | 2 | 1 | 4 | 3645.558 | -0.026 | 0.010 |
| 2 | 1 | 2 | 3 | | 1 | 1 | 1 | 2 | 4058.744 | -0.004 | 0.010 |
| 2 | 1 | 2 | 2 | | 1 | 1 | 1 | 2 | 4059.716 | -0.019 | 0.010 |
| 2 | 1 | 2 | 2 | | 1 | 1 | 1 | 1 | 4059.852 | 0.004 | 0.010 |
| 2 | 0 | 2 | 1 | | 1 | 0 | 1 | 1 | 4277.990 | 0.011 | 0.010 |
| 2 | 0 | 2 | 3 | | 1 | 0 | 1 | 2 | 4279.694 | 0.016 | 0.010 |
| 2 | 0 | 2 | 2 | | 1 | 0 | 1 | 1 | 4279.886 | 0.028 | 0.010 |
| 2 | 0 | 2 | 1 | | 1 | 0 | 1 | 0 | 4280.579 | -0.003 | 0.010 |
| 2 | 0 | 2 | 2 | | 1 | 0 | 1 | 2 | 4280.896 | 0.009 | 0.010 |
| 3 | 0 | 3 | 2 | | 2 | 1 | 1 | 1 | 4895.313 | -0.008 | 0.010 |
| 3 | 0 | 3 | 4 | | 2 | 1 | 1 | 3 | 4895.731 | -0.007 | 0.010 |
| 3 | 0 | 3 | 3 | | 2 | 1 | 1 | 2 | 4896.993 | -0.007 | 0.010 |
| 3 | 1 | 2 | 4 | | 2 | 2 | 0 | 3 | 4924.711 | 0.011 | 0.010 |
| 3 | 3 | 1 | 2 | | 3 | 1 | 2 | 3 | 5577.664 | 0.003 | 0.010 |
| 2 | 1 | 1 | 1 | | 1 | 0 | 1 | 1 | 5623.854 | -0.005 | 0.010 |
| 2 | 1 | 1 | 2 | | 1 | 0 | 1 | 1 | 5624.037 | -0.008 | 0.010 |
| 2 | 1 | 1 | 3 | | 1 | 0 | 1 | 2 | 5624.946 | -0.007 | 0.010 |
| 2 | 1 | 1 | 1 | | 1 | 0 | 1 | 0 | 5626.448 | -0.015 | 0.010 |
| 3 | 1 | 3 | 2 | | 2 | 1 | 2 | 1 | 6040.334 | -0.053 | 0.010 |
| 3 | 1 | 3 | 4 | | 2 | 1 | 2 | 3 | 6040.334 | 0.027 | 0.010 |
| 3 | 1 | 3 | 3 | | 2 | 1 | 2 | 2 | 6040.668 | 0.008 | 0.010 |
| 3 | 0 | 3 | 4 | | 2 | 0 | 2 | 3 | 6241.035 | 0.021 | 0.010 |
| 3 | 0 | 3 | 3 | | 2 | 0 | 2 | 2 | 6241.214 | 0.027 | 0.010 |
| 3 | 0 | 3 | 2 | | 2 | 0 | 2 | 1 | 6241.214 | 0.012 | 0.010 |
| 3 | 2 | 2 | 2 | | 2 | 2 | 1 | 1 | 6548.129 | -0.015 | 0.010 |
| 3 | 2 | 2 | 4 | | 2 | 2 | 1 | 3 | 6548.735 | -0.027 | 0.010 |
| 3 | 2 | 2 | 3 | | 2 | 2 | 1 | 2 | 6549.846 | -0.024 | 0.010 |
| 2 | 2 | 0 | 2 | | 1 | 1 | 0 | 2 | 6692.457 | 0.002 | 0.010 |
| 2 | 2 | 0 | 1 | | 1 | 1 | 0 | 0 | 6692.934 | -0.002 | 0.010 |
| 2 | 2 | 0 | 2 | | 1 | 1 | 0 | 1 | 6693.363 | -0.012 | 0.010 |
| 2 | 2 | 0 | 3 | | 1 | 1 | 0 | 2 | 6693.685 | 0.021 | 0.010 |
| 2 | 2 | 0 | 1 | | 1 | 1 | 0 | 1 | 6695.282 | 0.021 | 0.010 |
| 3 | 2 | 1 | 2 | | 2 | 2 | 0 | 1 | 6856.096 | -0.026 | 0.010 |
| 3 | 2 | 1 | 4 | | 2 | 2 | 0 | 3 | 6856.704 | -0.012 | 0.010 |
| 3 | 2 | 1 | 3 | | 2 | 2 | 0 | 2 | 6857.669 | -0.022 | 0.010 |
| 2 | 2 | 1 | 3 | | 1 | 1 | 1 | 2 | 6914.573 | 0.009 | 0.010 |
| 3 | 1 | 2 | 2 | | 2 | 1 | 1 | 1 | 6945.450 | 0.022 | 0.010 |
| 3 | 1 | 2 | 4 | | 2 | 1 | 1 | 3 | 6945.546 | 0.026 | 0.010 |
| 3 | 1 | 2 | 3 | | 2 | 1 | 1 | 2 | 6945.872 | 0.019 | 0.010 |

Table S38: Experimental rotational transition frequencies (MHz) for the #1:1 water complex of #1-DEAcA (aaAs:1w).

| $J'$ | $K'_a$ | $K'_c$ | $F'$ | - | $J''$ | $K''_a$ | $K''_c$ | $F''$ | $\nu_{\text{obs}}$ | $\nu_{\text{obs}} - \nu_{\text{calc}}$ | Err. |
|---|---|---|---|---|---|---|---|---|---|---|---|
| 1 | 1 | 0 | 1 | | 0 | 0 | 0 | 1 | 2172.770 | 0.003 | 0.010 |
| 1 | 1 | 0 | 2 | | 0 | 0 | 0 | 1 | 2174.846 | 0.006 | 0.010 |
| 1 | 1 | 0 | 0 | | 0 | 0 | 0 | 1 | 2177.948 | -0.002 | 0.010 |
| 2 | 1 | 2 | 2 | | 1 | 1 | 1 | 1 | 3638.726 | -0.005 | 0.010 |
| 2 | 1 | 2 | 2 | | 1 | 1 | 1 | 2 | 3638.867 | 0.006 | 0.010 |
| 2 | 0 | 2 | 1 | | 1 | 0 | 1 | 1 | 3701.262 | -0.003 | 0.010 |
| 2 | 0 | 2 | 3 | | 1 | 0 | 1 | 2 | 3704.525 | -0.003 | 0.010 |
| 2 | 0 | 2 | 2 | | 1 | 0 | 1 | 1 | 3704.958 | -0.008 | 0.010 |
| 2 | 0 | 2 | 1 | | 1 | 0 | 1 | 0 | 3706.124 | 0.006 | 0.010 |
| 2 | 0 | 2 | 2 | | 1 | 0 | 1 | 2 | 3706.908 | 0.001 | 0.010 |
| 2 | 1 | 1 | 1 | | 1 | 1 | 0 | 0 | 3813.742 | -0.003 | 0.010 |
| 2 | 1 | 1 | 2 | | 1 | 1 | 0 | 2 | 3816.629 | -0.005 | 0.010 |
| 2 | 1 | 1 | 3 | | 1 | 1 | 0 | 2 | 3816.776 | 0.003 | 0.010 |
| 2 | 1 | 1 | 2 | | 1 | 1 | 0 | 1 | 3818.704 | -0.003 | 0.010 |
| 2 | 1 | 1 | 1 | | 1 | 1 | 0 | 1 | 3818.935 | 0.007 | 0.010 |
| 2 | 1 | 1 | 2 | | 1 | 0 | 1 | 1 | 4126.304 | 0.003 | 0.010 |
| 2 | 1 | 1 | 3 | | 1 | 0 | 1 | 2 | 4128.389 | 0.006 | 0.010 |
| 2 | 1 | 1 | 1 | | 1 | 0 | 1 | 0 | 4131.382 | 0.006 | 0.010 |
| 2 | 2 | 0 | 2 | | 1 | 1 | 0 | 1 | 4502.117 | -0.070 | 0.010 |
| 2 | 2 | 0 | 3 | | 1 | 1 | 0 | 2 | 4502.506 | 0.012 | 0.010 |
| 2 | 2 | 1 | 2 | | 1 | 1 | 1 | 1 | 4568.213 | 0.006 | 0.010 |
| 2 | 2 | 1 | 2 | | 1 | 1 | 1 | 2 | 4568.364 | 0.028 | 0.010 |
| 2 | 2 | 1 | 3 | | 1 | 1 | 1 | 2 | 4570.423 | 0.006 | 0.010 |
| 2 | 2 | 1 | 1 | | 1 | 1 | 1 | 1 | 4571.434 | -0.010 | 0.010 |
| 2 | 2 | 1 | 1 | | 1 | 1 | 1 | 0 | 4571.774 | 0.004 | 0.010 |
| 3 | 1 | 3 | 4 | | 2 | 1 | 2 | 3 | 5442.800 | -0.010 | 0.010 |
| 3 | 1 | 3 | 2 | | 2 | 1 | 2 | 1 | 5443.014 | -0.004 | 0.010 |
| 3 | 1 | 3 | 3 | | 2 | 1 | 2 | 2 | 5443.494 | -0.022 | 0.010 |
| 3 | 0 | 3 | 4 | | 2 | 0 | 2 | 3 | 5508.804 | 0.005 | 0.010 |
| 3 | 0 | 3 | 2 | | 2 | 0 | 2 | 1 | 5509.123 | -0.001 | 0.010 |
| 3 | 0 | 3 | 3 | | 2 | 0 | 2 | 2 | 5509.278 | 0.011 | 0.010 |
| 3 | 2 | 2 | 2 | | 2 | 2 | 1 | 1 | 5589.000 | -0.012 | 0.010 |
| 3 | 2 | 2 | 4 | | 2 | 2 | 1 | 3 | 5590.156 | -0.011 | 0.010 |
| 3 | 2 | 2 | 3 | | 2 | 2 | 1 | 2 | 5592.238 | -0.010 | 0.010 |
| 3 | 2 | 1 | 2 | | 2 | 2 | 0 | 1 | 5670.834 | -0.007 | 0.010 |
| 3 | 2 | 1 | 4 | | 2 | 2 | 0 | 3 | 5671.916 | -0.005 | 0.010 |
| 3 | 2 | 1 | 3 | | 2 | 2 | 0 | 2 | 5673.607 | -0.004 | 0.010 |
| 3 | 1 | 2 | 2 | | 2 | 0 | 2 | 2 | 6130.552 | 0.008 | 0.010 |
| 3 | 1 | 2 | 3 | | 2 | 0 | 2 | 2 | 6131.245 | 0.010 | 0.010 |
| 3 | 1 | 2 | 4 | | 2 | 0 | 2 | 3 | 6133.109 | 0.008 | 0.010 |
| 3 | 1 | 2 | 3 | | 2 | 0 | 2 | 3 | 6133.611 | -0.004 | 0.010 |
| 3 | 1 | 2 | 2 | | 2 | 0 | 2 | 1 | 6134.254 | 0.009 | 0.010 |
| 4 | 1 | 4 | 3 | | 3 | 2 | 2 | 2 | 6156.063 | 0.014 | 0.010 |
| 4 | 1 | 4 | 5 | | 3 | 2 | 2 | 4 | 6156.894 | -0.010 | 0.010 |
| 4 | 1 | 4 | 4 | | 3 | 2 | 2 | 3 | 6160.239 | 0.013 | 0.010 |
| 3 | 2 | 1 | 3 | | 2 | 1 | 1 | 3 | 6356.943 | -0.009 | 0.010 |
| 3 | 2 | 1 | 3 | | 2 | 1 | 1 | 2 | 6357.105 | 0.013 | 0.010 |
| 3 | 2 | 1 | 4 | | 2 | 1 | 1 | 3 | 6357.645 | 0.003 | 0.010 |
| 3 | 2 | 1 | 2 | | 2 | 1 | 1 | 1 | 6357.806 | 0.002 | 0.010 |
| 3 | 2 | 2 | 3 | | 2 | 1 | 2 | 2 | 6521.717 | -0.006 | 0.010 |
| 3 | 2 | 2 | 4 | | 2 | 1 | 2 | 3 | 6523.947 | 0.003 | 0.010 |
| 3 | 2 | 2 | 2 | | 2 | 1 | 2 | 1 | 6525.188 | 0.010 | 0.010 |
| 4 | 0 | 4 | 3 | | 3 | 1 | 2 | 2 | 6658.252 | 0.018 | 0.010 |
| 4 | 0 | 4 | 5 | | 3 | 1 | 2 | 4 | 6658.900 | 0.007 | 0.010 |
| 4 | 0 | 4 | 4 | | 3 | 1 | 2 | 3 | 6661.631 | -0.004 | 0.010 |
| 3 | 3 | 0 | 3 | | 2 | 2 | 0 | 3 | 6911.564 | 0.005 | 0.010 |

Table S38: Experimental rotational transition frequencies (MHz) for the #1:1 water complex of #1-DEAcA (aaAs:1w) *(continued)*

| $J'$ | $K_a'$ | $K_c'$ | $F'$ | - | $J''$ | $K_a''$ | $K_c''$ | $F''$ | $\nu_{\rm obs}$ | $\nu_{\rm obs} - \nu_{\rm calc}$ | Err. |
|---|---|---|---|---|---|---|---|---|---|---|---|
| 3 | 3 | 0 | 3 | | 2 | 2 | 0 | 2 | 6913.937 | -0.002 | 0.010 |
| 3 | 3 | 0 | 2 | | 2 | 2 | 0 | 1 | 6914.035 | -0.003 | 0.010 |
| 3 | 3 | 0 | 4 | | 2 | 2 | 0 | 3 | 6914.378 | 0.003 | 0.010 |
| 3 | 3 | 0 | 2 | | 2 | 2 | 0 | 2 | 6917.744 | 0.003 | 0.010 |
| 4 | 1 | 3 | 3 | | 3 | 2 | 1 | 2 | 6928.472 | 0.024 | 0.010 |
| 4 | 1 | 3 | 5 | | 3 | 2 | 1 | 4 | 6928.961 | 0.025 | 0.010 |
| 3 | 3 | 1 | 3 | | 2 | 2 | 1 | 3 | 6930.419 | 0.007 | 0.010 |
| 4 | 1 | 3 | 4 | | 3 | 2 | 1 | 3 | 6930.607 | 0.025 | 0.010 |
| 3 | 3 | 1 | 3 | | 2 | 2 | 1 | 2 | 6932.494 | 0.002 | 0.010 |
| 3 | 3 | 1 | 2 | | 2 | 2 | 1 | 1 | 6933.025 | 0.014 | 0.010 |
| 3 | 3 | 1 | 4 | | 2 | 2 | 1 | 3 | 6933.202 | 0.008 | 0.010 |
| 3 | 3 | 1 | 2 | | 2 | 2 | 1 | 2 | 6936.251 | 0.002 | 0.010 |
| 4 | 2 | 3 | 3 | | 3 | 2 | 2 | 2 | 7435.958 | -0.011 | 0.010 |
| 4 | 2 | 3 | 5 | | 3 | 2 | 2 | 4 | 7436.212 | -0.004 | 0.010 |
| 4 | 2 | 3 | 4 | | 3 | 2 | 2 | 3 | 7437.168 | -0.007 | 0.010 |
| 4 | 3 | 2 | 3 | | 3 | 3 | 1 | 2 | 7488.911 | -0.031 | 0.010 |
| 4 | 3 | 2 | 5 | | 3 | 3 | 1 | 4 | 7489.642 | -0.024 | 0.010 |
| 4 | 3 | 2 | 4 | | 3 | 3 | 1 | 3 | 7491.448 | -0.033 | 0.010 |
| 4 | 3 | 1 | 3 | | 3 | 3 | 0 | 2 | 7509.657 | -0.024 | 0.010 |
| 4 | 3 | 1 | 5 | | 3 | 3 | 0 | 4 | 7510.358 | -0.023 | 0.010 |
| 4 | 3 | 1 | 4 | | 3 | 3 | 0 | 3 | 7512.080 | -0.012 | 0.010 |
| 4 | 2 | 2 | 3 | | 3 | 2 | 1 | 2 | 7606.424 | 0.015 | 0.010 |
| 4 | 2 | 2 | 5 | | 3 | 2 | 1 | 4 | 7606.651 | 0.023 | 0.010 |
| 4 | 2 | 2 | 4 | | 3 | 2 | 1 | 3 | 7607.247 | 0.016 | 0.010 |

Table S39: Experimental rotational transition frequencies (MHz) for the #1:2 water complex of #1-DEAcA (aaAs:2w)

| $J'$ | $K'_a$ | $K'_c$ | $F'$ | - | $J''$ | $K''_a$ | $K''_c$ | $F''$ | $\nu_{\text{obs}}$ | $\nu_{\text{obs}} - \nu_{\text{calc}}$ | Err. |
|---|---|---|---|---|---|---|---|---|---|---|---|
| 2 | 1 | 2 | 1 | | 1 | 1 | 1 | 1 | 2516.130 | -0.004 | 0.010 |
| 2 | 1 | 2 | 1 | | 1 | 1 | 1 | 0 | 2516.352 | 0.001 | 0.010 |
| 2 | 1 | 2 | 3 | | 1 | 1 | 1 | 2 | 2517.439 | 0.003 | 0.010 |
| 2 | 1 | 2 | 2 | | 1 | 1 | 1 | 1 | 2519.537 | -0.006 | 0.010 |
| 2 | 1 | 2 | 2 | | 1 | 1 | 1 | 2 | 2519.633 | 0.004 | 0.010 |
| 2 | 0 | 2 | 1 | | 1 | 0 | 1 | 1 | 2546.998 | -0.002 | 0.010 |
| 2 | 0 | 2 | 3 | | 1 | 0 | 1 | 2 | 2550.161 | -0.001 | 0.010 |
| 2 | 0 | 2 | 2 | | 1 | 0 | 1 | 1 | 2550.368 | -0.003 | 0.010 |
| 2 | 0 | 2 | 1 | | 1 | 0 | 1 | 0 | 2551.898 | 0.003 | 0.010 |
| 2 | 0 | 2 | 2 | | 1 | 0 | 1 | 2 | 2552.332 | 0.002 | 0.010 |
| 2 | 1 | 1 | 1 | | 1 | 1 | 0 | 0 | 2582.671 | 0.000 | 0.010 |
| 2 | 1 | 1 | 2 | | 1 | 1 | 0 | 2 | 2585.574 | -0.017 | 0.010 |
| 2 | 1 | 1 | 3 | | 1 | 1 | 0 | 2 | 2585.686 | 0.002 | 0.010 |
| 2 | 1 | 1 | 2 | | 1 | 1 | 0 | 1 | 2587.638 | 0.000 | 0.010 |
| 2 | 1 | 1 | 1 | | 1 | 1 | 0 | 1 | 2587.794 | 0.005 | 0.010 |
| 2 | 1 | 1 | 2 | | 1 | 0 | 1 | 1 | 3131.336 | 0.006 | 0.010 |
| 2 | 1 | 1 | 3 | | 1 | 0 | 1 | 2 | 3133.389 | 0.007 | 0.010 |
| 2 | 1 | 1 | 1 | | 1 | 0 | 1 | 0 | 3136.376 | 0.000 | 0.010 |
| 3 | 0 | 3 | 2 | | 2 | 1 | 1 | 1 | 3237.193 | 0.006 | 0.010 |
| 3 | 0 | 3 | 4 | | 2 | 1 | 1 | 3 | 3238.072 | 0.004 | 0.010 |
| 3 | 0 | 3 | 3 | | 2 | 1 | 1 | 2 | 3240.523 | 0.005 | 0.010 |
| 3 | 1 | 3 | 2 | | 2 | 1 | 2 | 2 | 3772.443 | -0.003 | 0.010 |
| 3 | 1 | 3 | 4 | | 2 | 1 | 2 | 3 | 3775.631 | -0.000 | 0.010 |
| 3 | 1 | 3 | 2 | | 2 | 1 | 2 | 1 | 3775.843 | -0.012 | 0.010 |
| 3 | 1 | 3 | 3 | | 2 | 1 | 2 | 2 | 3776.279 | 0.000 | 0.010 |
| 3 | 1 | 3 | 3 | | 2 | 1 | 2 | 3 | 3778.468 | -0.004 | 0.010 |
| 3 | 0 | 3 | 2 | | 2 | 0 | 2 | 2 | 3818.297 | 0.001 | 0.010 |
| 3 | 0 | 3 | 4 | | 2 | 0 | 2 | 3 | 3821.295 | 0.007 | 0.010 |
| 3 | 0 | 3 | 3 | | 2 | 0 | 2 | 2 | 3821.476 | -0.001 | 0.010 |
| 3 | 0 | 3 | 2 | | 2 | 0 | 2 | 1 | 3821.665 | -0.003 | 0.010 |
| 3 | 0 | 3 | 3 | | 2 | 0 | 2 | 3 | 3823.649 | 0.004 | 0.010 |
| 3 | 2 | 2 | 2 | | 2 | 2 | 1 | 1 | 3826.288 | -0.008 | 0.010 |
| 3 | 2 | 2 | 4 | | 2 | 2 | 1 | 3 | 3827.456 | -0.005 | 0.010 |
| 3 | 2 | 2 | 3 | | 2 | 2 | 1 | 2 | 3829.556 | -0.004 | 0.010 |
| 3 | 2 | 1 | 2 | | 2 | 2 | 0 | 1 | 3832.883 | 0.001 | 0.010 |
| 3 | 2 | 1 | 3 | | 2 | 2 | 0 | 3 | 3833.830 | -0.013 | 0.010 |
| 3 | 2 | 1 | 4 | | 2 | 2 | 0 | 3 | 3834.019 | -0.003 | 0.010 |
| 3 | 2 | 1 | 3 | | 2 | 2 | 0 | 2 | 3836.007 | -0.004 | 0.010 |
| 3 | 2 | 1 | 2 | | 2 | 2 | 0 | 2 | 3836.263 | 0.006 | 0.010 |
| 3 | 1 | 2 | 2 | | 2 | 1 | 1 | 1 | 3877.591 | -0.001 | 0.010 |
| 3 | 1 | 2 | 4 | | 2 | 1 | 1 | 3 | 3877.806 | -0.005 | 0.010 |
| 3 | 1 | 2 | 3 | | 2 | 1 | 1 | 2 | 3878.373 | -0.001 | 0.010 |
| 2 | 2 | 0 | 2 | | 1 | 1 | 0 | 2 | 4124.570 | 0.010 | 0.010 |
| 2 | 2 | 0 | 1 | | 1 | 1 | 0 | 0 | 4124.880 | 0.017 | 0.010 |
| 2 | 2 | 0 | 2 | | 1 | 1 | 0 | 1 | 4126.612 | 0.005 | 0.010 |
| 2 | 2 | 0 | 3 | | 1 | 1 | 0 | 2 | 4126.738 | 0.009 | 0.010 |
| 2 | 2 | 0 | 1 | | 1 | 1 | 0 | 1 | 4129.992 | 0.011 | 0.010 |
| 2 | 2 | 1 | 3 | | 1 | 1 | 1 | 2 | 4159.485 | 0.007 | 0.010 |
| 2 | 2 | 1 | 1 | | 1 | 1 | 1 | 0 | 4160.781 | 0.004 | 0.010 |
| 4 | 0 | 4 | 3 | | 3 | 1 | 2 | 2 | 4447.405 | 0.011 | 0.010 |
| 4 | 0 | 4 | 5 | | 3 | 1 | 2 | 4 | 4447.889 | 0.001 | 0.010 |
| 4 | 0 | 4 | 4 | | 3 | 1 | 2 | 3 | 4449.977 | 0.006 | 0.010 |
| 3 | 1 | 2 | 3 | | 2 | 0 | 2 | 2 | 4459.338 | 0.005 | 0.010 |
| 3 | 1 | 2 | 4 | | 2 | 0 | 2 | 3 | 4461.032 | 0.001 | 0.010 |
| 3 | 1 | 2 | 2 | | 2 | 0 | 2 | 1 | 4462.074 | 0.002 | 0.010 |
| 4 | 1 | 4 | 5 | | 3 | 1 | 3 | 4 | 5032.526 | 0.000 | 0.010 |
| 4 | 1 | 4 | 3 | | 3 | 1 | 3 | 2 | 5032.708 | 0.001 | 0.010 |

Table S39: Experimental rotational transition frequencies (MHz) for the #1:2 water complex of #1-DEAcA (aaAs:2w) (aaAs:2w).*(continued)*

| $J'$ | $K_a'$ | $K_c'$ | $F'$ | - | $J''$ | $K_a''$ | $K_c''$ | $F''$ | $\nu_{\rm obs}$ | $\nu_{\rm obs}-\nu_{\rm calc}$ | Err. |
|---|---|---|---|---|---|---|---|---|---|---|---|
| 4 | 1 | 4 | 4 | | 3 | 1 | 3 | 3 | 5032.848 | 0.002 | 0.010 |
| 4 | 0 | 4 | 5 | | 3 | 0 | 3 | 4 | 5087.631 | 0.001 | 0.010 |
| 4 | 0 | 4 | 3 | | 3 | 0 | 3 | 2 | 5087.776 | -0.022 | 0.010 |
| 4 | 0 | 4 | 4 | | 3 | 0 | 3 | 3 | 5087.837 | 0.010 | 0.010 |
| 4 | 2 | 3 | 3 | | 3 | 2 | 2 | 2 | 5102.137 | -0.001 | 0.010 |
| 4 | 2 | 3 | 5 | | 3 | 2 | 2 | 4 | 5102.365 | -0.007 | 0.010 |
| 4 | 2 | 3 | 4 | | 3 | 2 | 2 | 3 | 5103.269 | -0.011 | 0.010 |
| 4 | 1 | 3 | 5 | | 3 | 1 | 2 | 4 | 5168.477 | 0.002 | 0.010 |
| 4 | 1 | 3 | 3 | | 3 | 1 | 2 | 2 | 5168.477 | 0.029 | 0.010 |
| 4 | 1 | 3 | 4 | | 3 | 1 | 2 | 3 | 5168.745 | -0.001 | 0.010 |
| 3 | 2 | 1 | 3 | | 2 | 1 | 1 | 2 | 5374.988 | 0.008 | 0.010 |
| 3 | 2 | 1 | 2 | | 2 | 1 | 1 | 1 | 5375.076 | 0.002 | 0.010 |
| 3 | 2 | 1 | 4 | | 2 | 1 | 1 | 3 | 5375.076 | 0.010 | 0.010 |
| 3 | 2 | 2 | 3 | | 2 | 1 | 2 | 2 | 5467.316 | 0.005 | 0.010 |
| 3 | 2 | 2 | 4 | | 2 | 1 | 2 | 3 | 5469.506 | 0.003 | 0.010 |
| 3 | 2 | 2 | 2 | | 2 | 1 | 2 | 1 | 5470.721 | -0.001 | 0.010 |
| 5 | 0 | 5 | 4 | | 4 | 1 | 3 | 3 | 5627.101 | 0.002 | 0.010 |
| 5 | 0 | 5 | 6 | | 4 | 1 | 3 | 5 | 5627.477 | 0.004 | 0.010 |
| 5 | 0 | 5 | 5 | | 4 | 1 | 3 | 4 | 5629.504 | 0.008 | 0.010 |
| 4 | 1 | 3 | 4 | | 3 | 0 | 3 | 3 | 5806.612 | 0.010 | 0.010 |
| 4 | 1 | 3 | 5 | | 3 | 0 | 3 | 4 | 5808.218 | 0.000 | 0.010 |
| 4 | 1 | 3 | 3 | | 3 | 0 | 3 | 2 | 5808.854 | 0.001 | 0.010 |
| 5 | 1 | 5 | 4 | | 4 | 1 | 4 | 4 | 6284.126 | 0.012 | 0.010 |
| 5 | 1 | 5 | 6 | | 4 | 1 | 4 | 5 | 6287.932 | -0.026 | 0.010 |
| 5 | 1 | 5 | 5 | | 4 | 1 | 4 | 4 | 6288.114 | -0.046 | 0.010 |
| 5 | 1 | 5 | 4 | | 4 | 1 | 4 | 3 | 6288.114 | 0.028 | 0.010 |
| 5 | 0 | 5 | 4 | | 4 | 0 | 4 | 4 | 6344.965 | 0.021 | 0.010 |
| 6 | 1 | 5 | 5 | | 5 | 2 | 3 | 4 | 6345.403 | 0.005 | 0.010 |
| 6 | 1 | 5 | 7 | | 5 | 2 | 3 | 6 | 6345.403 | 0.008 | 0.010 |
| 6 | 1 | 5 | 6 | | 5 | 2 | 3 | 5 | 6345.572 | 0.013 | 0.010 |
| 5 | 0 | 5 | 6 | | 4 | 0 | 4 | 5 | 6348.079 | 0.019 | 0.010 |
| 5 | 0 | 5 | 4 | | 4 | 0 | 4 | 3 | 6348.139 | -0.015 | 0.010 |
| 5 | 0 | 5 | 5 | | 4 | 0 | 4 | 4 | 6348.275 | 0.004 | 0.010 |
| 5 | 2 | 4 | 4 | | 4 | 2 | 3 | 3 | 6375.980 | -0.022 | 0.010 |
| 5 | 2 | 4 | 6 | | 4 | 2 | 3 | 5 | 6376.049 | -0.003 | 0.010 |
| 5 | 2 | 4 | 5 | | 4 | 2 | 3 | 4 | 6376.538 | -0.009 | 0.010 |
| 5 | 4 | 1 | 6 | | 4 | 4 | 0 | 5 | 6383.016 | -0.032 | 0.010 |
| 5 | 4 | 2 | 6 | | 4 | 4 | 1 | 5 | 6383.016 | -0.021 | 0.010 |
| 5 | 3 | 3 | 4 | | 4 | 3 | 2 | 3 | 6384.455 | -0.013 | 0.010 |
| 5 | 3 | 3 | 6 | | 4 | 3 | 2 | 5 | 6384.690 | -0.022 | 0.010 |
| 5 | 4 | 1 | 5 | | 4 | 4 | 0 | 4 | 6384.811 | 0.030 | 0.010 |
| 5 | 4 | 2 | 5 | | 4 | 4 | 1 | 4 | 6384.811 | 0.041 | 0.010 |
| 5 | 3 | 2 | 4 | | 4 | 3 | 1 | 3 | 6385.491 | -0.015 | 0.010 |
| 5 | 3 | 3 | 5 | | 4 | 3 | 2 | 4 | 6385.670 | -0.019 | 0.010 |
| 5 | 3 | 2 | 6 | | 4 | 3 | 1 | 5 | 6385.735 | -0.013 | 0.010 |
| 5 | 3 | 2 | 5 | | 4 | 3 | 1 | 4 | 6386.698 | -0.015 | 0.010 |
| 5 | 2 | 3 | 4 | | 4 | 2 | 2 | 3 | 6407.714 | -0.041 | 0.010 |
| 5 | 2 | 3 | 6 | | 4 | 2 | 2 | 5 | 6407.805 | 0.016 | 0.010 |
| 5 | 2 | 3 | 5 | | 4 | 2 | 2 | 4 | 6408.106 | 0.000 | 0.010 |
| 5 | 1 | 4 | 4 | | 4 | 1 | 3 | 4 | 6456.319 | 0.045 | 0.010 |
| 5 | 1 | 4 | 4 | | 4 | 1 | 3 | 3 | 6457.211 | 0.007 | 0.010 |
| 5 | 1 | 4 | 6 | | 4 | 1 | 3 | 5 | 6457.211 | 0.010 | 0.010 |
| 5 | 1 | 4 | 5 | | 4 | 1 | 3 | 4 | 6457.379 | 0.002 | 0.010 |
| 5 | 1 | 4 | 5 | | 4 | 1 | 3 | 5 | 6458.133 | 0.014 | 0.010 |
| 3 | 3 | 0 | 3 | | 2 | 2 | 0 | 2 | 6476.995 | 0.001 | 0.010 |
| 3 | 3 | 0 | 2 | | 2 | 2 | 0 | 1 | 6477.324 | -0.000 | 0.010 |

Table S39: Experimental rotational transition frequencies (MHz) for the #1:2 water complex of #1-DEAcA (aaAs:2w) (aaAs:2w).*(continued)*

| $J'$ | $K_a'$ | $K_c'$ | $F'$ | - | $J''$ | $K_a''$ | $K_c''$ | $F''$ | $\nu_{\mathrm{obs}}$ | $\nu_{\mathrm{obs}} - \nu_{\mathrm{calc}}$ | Err. |
|---|---|---|---|---|---|---|---|---|---|---|---|
| 3 | 3 | 0 | 4 | | 2 | 2 | 0 | 3 | 6477.571 | 0.002 | 0.010 |
| 3 | 3 | 1 | 3 | | 2 | 2 | 1 | 2 | 6478.528 | -0.003 | 0.010 |
| 3 | 3 | 1 | 2 | | 2 | 2 | 1 | 1 | 6478.966 | -0.002 | 0.010 |
| 3 | 3 | 1 | 4 | | 2 | 2 | 1 | 3 | 6479.172 | -0.003 | 0.010 |
| 4 | 2 | 2 | 3 | | 3 | 1 | 2 | 2 | 6615.843 | -0.008 | 0.010 |
| 4 | 2 | 2 | 5 | | 3 | 1 | 2 | 4 | 6615.843 | 0.006 | 0.010 |
| 4 | 2 | 2 | 4 | | 3 | 1 | 2 | 3 | 6615.942 | -0.004 | 0.010 |
| 4 | 2 | 3 | 4 | | 3 | 1 | 3 | 3 | 6794.310 | -0.002 | 0.010 |
| 4 | 2 | 3 | 5 | | 3 | 1 | 3 | 4 | 6796.238 | -0.006 | 0.010 |
| 4 | 2 | 3 | 3 | | 3 | 1 | 3 | 2 | 6796.998 | -0.007 | 0.010 |
| 5 | 1 | 4 | 5 | | 4 | 0 | 4 | 4 | 7176.157 | 0.004 | 0.010 |
| 5 | 1 | 4 | 6 | | 4 | 0 | 4 | 5 | 7177.793 | 0.005 | 0.010 |
| 5 | 1 | 4 | 4 | | 4 | 0 | 4 | 3 | 7178.266 | 0.008 | 0.010 |
| 6 | 1 | 6 | 7 | | 5 | 1 | 5 | 6 | 7541.777 | 0.005 | 0.010 |
| 6 | 1 | 6 | 6 | | 5 | 1 | 5 | 5 | 7541.929 | 0.008 | 0.010 |
| 6 | 0 | 6 | 7 | | 5 | 0 | 5 | 6 | 7602.083 | -0.009 | 0.010 |
| 6 | 0 | 6 | 5 | | 5 | 0 | 5 | 4 | 7602.167 | 0.016 | 0.010 |
| 6 | 0 | 6 | 6 | | 5 | 0 | 5 | 5 | 7602.314 | 0.002 | 0.010 |
| 6 | 2 | 5 | 7 | | 5 | 2 | 4 | 6 | 7648.304 | -0.010 | 0.010 |
| 6 | 2 | 5 | 5 | | 5 | 2 | 4 | 4 | 7648.304 | -0.004 | 0.010 |
| 6 | 2 | 5 | 6 | | 5 | 2 | 4 | 5 | 7648.606 | -0.019 | 0.010 |
| 6 | 3 | 4 | 7 | | 5 | 3 | 3 | 6 | 7663.260 | -0.047 | 0.010 |
| 6 | 3 | 4 | 5 | | 5 | 3 | 3 | 4 | 7663.260 | 0.044 | 0.010 |
| 6 | 3 | 4 | 6 | | 5 | 3 | 3 | 5 | 7663.874 | 0.002 | 0.010 |
| 6 | 3 | 3 | 7 | | 5 | 3 | 2 | 6 | 7666.013 | -0.037 | 0.010 |
| 6 | 3 | 3 | 5 | | 5 | 3 | 2 | 4 | 7666.013 | 0.051 | 0.010 |
| 6 | 3 | 3 | 6 | | 5 | 3 | 2 | 5 | 7666.584 | -0.010 | 0.010 |
| 6 | 2 | 4 | 7 | | 5 | 2 | 3 | 6 | 7701.940 | 0.009 | 0.010 |
| 6 | 2 | 4 | 5 | | 5 | 2 | 3 | 4 | 7701.940 | -0.002 | 0.010 |
| 6 | 2 | 4 | 6 | | 5 | 2 | 3 | 5 | 7702.009 | -0.040 | 0.010 |
| 6 | 1 | 5 | 7 | | 5 | 1 | 4 | 6 | 7743.357 | 0.012 | 0.010 |
| 6 | 1 | 5 | 5 | | 5 | 1 | 4 | 4 | 7743.357 | 0.005 | 0.010 |
| 6 | 1 | 5 | 6 | | 5 | 1 | 4 | 5 | 7743.506 | 0.018 | 0.010 |
| 4 | 3 | 1 | 4 | | 3 | 2 | 1 | 3 | 7749.729 | -0.017 | 0.010 |
| 4 | 3 | 1 | 5 | | 3 | 2 | 1 | 4 | 7750.400 | -0.006 | 0.010 |
| 4 | 3 | 1 | 3 | | 3 | 2 | 1 | 2 | 7750.549 | -0.010 | 0.010 |
| 4 | 3 | 2 | 4 | | 3 | 2 | 2 | 3 | 7757.432 | -0.009 | 0.010 |
| 4 | 3 | 2 | 5 | | 3 | 2 | 2 | 4 | 7758.286 | 0.010 | 0.010 |
| 4 | 3 | 2 | 3 | | 3 | 2 | 2 | 2 | 7758.487 | -0.002 | 0.010 |
| 5 | 2 | 3 | 4 | | 4 | 1 | 3 | 3 | 7855.169 | 0.011 | 0.010 |
| 5 | 2 | 3 | 6 | | 4 | 1 | 3 | 5 | 7855.169 | 0.018 | 0.010 |
| 5 | 2 | 3 | 5 | | 4 | 1 | 3 | 4 | 7855.302 | -0.004 | 0.010 |

Table S40: Experimental rotational transition frequencies (MHz) for the #1:3 water complex of #1-DEAcA (aaAs:3w).

| $J'$ | $K_a'$ | $K_c'$ | $F'$ | - | $J''$ | $K_a''$ | $K_c''$ | $F''$ | $\nu_{\text{obs}}$ | $\nu_{\text{obs}} - \nu_{\text{calc}}$ | Err. |
|---|---|---|---|---|---|---|---|---|---|---|---|
| 3 | 1 | 3 | 2 | | 2 | 1 | 2 | 1 | 2624.100 | -0.003 | 0.010 |
| 3 | 1 | 3 | 4 | | 2 | 1 | 2 | 3 | 2624.319 | 0.008 | 0.010 |
| 3 | 1 | 3 | 3 | | 2 | 1 | 2 | 2 | 2624.830 | -0.005 | 0.010 |
| 3 | 0 | 3 | 4 | | 2 | 0 | 2 | 3 | 2639.272 | 0.011 | 0.010 |
| 3 | 0 | 3 | 3 | | 2 | 0 | 2 | 2 | 2639.272 | -0.033 | 0.010 |
| 3 | 0 | 3 | 2 | | 2 | 0 | 2 | 1 | 2639.675 | 0.008 | 0.010 |
| 3 | 1 | 2 | 4 | | 2 | 1 | 1 | 3 | 2654.937 | -0.000 | 0.010 |
| 3 | 1 | 2 | 2 | | 2 | 1 | 1 | 1 | 2655.149 | -0.019 | 0.010 |
| 3 | 1 | 2 | 3 | | 2 | 1 | 1 | 2 | 2655.539 | -0.008 | 0.010 |
| 4 | 1 | 4 | 3 | | 3 | 1 | 3 | 2 | 3499.016 | 0.018 | 0.010 |
| 4 | 1 | 4 | 5 | | 3 | 1 | 3 | 4 | 3499.016 | 0.000 | 0.010 |
| 4 | 1 | 4 | 4 | | 3 | 1 | 3 | 3 | 3499.234 | 0.000 | 0.010 |
| 4 | 0 | 4 | 4 | | 3 | 0 | 3 | 3 | 3518.409 | 0.001 | 0.010 |
| 4 | 0 | 4 | 5 | | 3 | 0 | 3 | 4 | 3518.409 | 0.004 | 0.010 |
| 4 | 0 | 4 | 3 | | 3 | 0 | 3 | 2 | 3518.598 | 0.001 | 0.010 |
| 4 | 2 | 3 | 3 | | 3 | 2 | 2 | 2 | 3519.237 | 0.009 | 0.010 |
| 4 | 2 | 3 | 5 | | 3 | 2 | 2 | 4 | 3519.453 | 0.000 | 0.010 |
| 4 | 2 | 3 | 4 | | 3 | 2 | 2 | 3 | 3520.315 | -0.010 | 0.010 |
| 4 | 2 | 2 | 3 | | 3 | 2 | 1 | 2 | 3520.613 | -0.007 | 0.010 |
| 4 | 2 | 2 | 5 | | 3 | 2 | 1 | 4 | 3520.850 | -0.002 | 0.010 |
| 4 | 2 | 2 | 4 | | 3 | 2 | 1 | 3 | 3521.764 | -0.005 | 0.010 |
| 4 | 1 | 3 | 5 | | 3 | 1 | 2 | 4 | 3539.907 | 0.003 | 0.010 |
| 4 | 1 | 3 | 3 | | 3 | 1 | 2 | 2 | 3540.085 | -0.006 | 0.010 |
| 4 | 1 | 3 | 4 | | 3 | 1 | 2 | 3 | 3540.188 | 0.013 | 0.010 |
| 5 | 1 | 5 | 4 | | 4 | 1 | 4 | 3 | 4373.545 | -0.009 | 0.010 |
| 5 | 1 | 5 | 6 | | 4 | 1 | 4 | 5 | 4373.545 | 0.002 | 0.010 |
| 5 | 1 | 5 | 5 | | 4 | 1 | 4 | 4 | 4373.662 | 0.012 | 0.010 |
| 5 | 0 | 5 | 5 | | 4 | 0 | 4 | 4 | 4396.969 | 0.022 | 0.010 |
| 5 | 0 | 5 | 6 | | 4 | 0 | 4 | 5 | 4396.969 | -0.002 | 0.010 |
| 5 | 0 | 5 | 4 | | 4 | 0 | 4 | 3 | 4397.089 | 0.002 | 0.010 |
| 5 | 2 | 4 | 6 | | 4 | 2 | 3 | 5 | 4399.273 | -0.004 | 0.010 |
| 5 | 2 | 4 | 5 | | 4 | 2 | 3 | 4 | 4399.735 | 0.008 | 0.010 |
| 5 | 2 | 3 | 4 | | 4 | 2 | 2 | 3 | 4402.069 | 0.042 | 0.010 |
| 5 | 2 | 3 | 6 | | 4 | 2 | 2 | 5 | 4402.069 | -0.010 | 0.010 |
| 5 | 2 | 3 | 5 | | 4 | 2 | 2 | 4 | 4402.595 | 0.008 | 0.010 |
| 5 | 1 | 4 | 6 | | 4 | 1 | 3 | 5 | 4424.667 | 0.001 | 0.010 |
| 5 | 1 | 4 | 5 | | 4 | 1 | 3 | 4 | 4424.791 | -0.075 | 0.010 |
| 5 | 1 | 4 | 4 | | 4 | 1 | 3 | 3 | 4424.791 | 0.094 | 0.010 |
| 6 | 1 | 6 | 7 | | 5 | 1 | 5 | 6 | 5247.894 | 0.011 | 0.010 |
| 6 | 1 | 6 | 5 | | 5 | 1 | 5 | 4 | 5247.894 | -0.006 | 0.010 |
| 6 | 1 | 6 | 6 | | 5 | 1 | 5 | 5 | 5247.894 | -0.046 | 0.010 |
| 6 | 0 | 6 | 6 | | 5 | 0 | 5 | 5 | 5274.759 | -0.028 | 0.010 |
| 6 | 0 | 6 | 5 | | 5 | 0 | 5 | 4 | 5274.883 | -0.028 | 0.010 |
| 6 | 0 | 6 | 7 | | 5 | 0 | 5 | 6 | 5274.883 | 0.052 | 0.010 |
| 6 | 2 | 5 | 7 | | 5 | 2 | 4 | 6 | 5278.922 | -0.001 | 0.010 |
| 6 | 2 | 5 | 5 | | 5 | 2 | 4 | 4 | 5278.922 | -0.002 | 0.010 |
| 6 | 2 | 5 | 6 | | 5 | 2 | 4 | 5 | 5279.187 | 0.003 | 0.010 |
| 6 | 2 | 4 | 7 | | 5 | 2 | 3 | 6 | 5283.815 | -0.005 | 0.010 |
| 6 | 2 | 4 | 5 | | 5 | 2 | 3 | 4 | 5283.815 | -0.001 | 0.010 |
| 6 | 2 | 4 | 6 | | 5 | 2 | 3 | 5 | 5284.145 | -0.005 | 0.010 |
| 6 | 1 | 5 | 7 | | 5 | 1 | 4 | 6 | 5309.209 | -0.001 | 0.010 |
| 6 | 1 | 5 | 6 | | 5 | 1 | 4 | 5 | 5309.333 | 0.097 | 0.010 |
| 6 | 1 | 5 | 5 | | 5 | 1 | 4 | 4 | 5309.333 | -0.077 | 0.010 |
| 7 | 1 | 7 | 8 | | 6 | 1 | 6 | 7 | 6122.023 | 0.009 | 0.010 |
| 7 | 1 | 7 | 6 | | 6 | 1 | 6 | 5 | 6122.023 | -0.006 | 0.010 |
| 7 | 1 | 7 | 7 | | 6 | 1 | 6 | 6 | 6122.023 | -0.020 | 0.010 |

Table S40: Experimental rotational transition frequencies (MHz) for the #1:3 water complex of #1-DEAcA (aaAs:3w) *(continued)*

| $J'$ | $K'_a$ | $K'_c$ | $F'$ | - | $J''$ | $K''_a$ | $K''_c$ | $F''$ | $\nu_{\rm obs}$ | $\nu_{\rm obs}-\nu_{\rm calc}$ | Err. |
|---|---|---|---|---|---|---|---|---|---|---|---|
| 7 | 0 | 7 | 7 | | 6 | 0 | 6 | 6 | 6151.870 | 0.063 | 0.010 |
| 7 | 0 | 7 | 8 | | 6 | 0 | 6 | 7 | 6151.870 | 0.002 | 0.010 |
| 7 | 0 | 7 | 6 | | 6 | 0 | 6 | 5 | 6151.870 | -0.057 | 0.010 |
| 7 | 2 | 6 | 8 | | 6 | 2 | 5 | 7 | 6158.385 | -0.007 | 0.010 |
| 7 | 2 | 6 | 6 | | 6 | 2 | 5 | 5 | 6158.385 | -0.022 | 0.010 |
| 7 | 2 | 6 | 7 | | 6 | 2 | 5 | 6 | 6158.547 | -0.007 | 0.010 |
| 7 | 2 | 5 | 8 | | 6 | 2 | 4 | 7 | 6166.202 | 0.005 | 0.010 |
| 7 | 2 | 5 | 6 | | 6 | 2 | 4 | 5 | 6166.202 | -0.004 | 0.010 |
| 7 | 2 | 5 | 7 | | 6 | 2 | 4 | 6 | 6166.438 | -0.001 | 0.010 |
| 7 | 1 | 6 | 8 | | 6 | 1 | 5 | 7 | 6193.532 | 0.033 | 0.010 |
| 7 | 1 | 6 | 7 | | 6 | 1 | 5 | 6 | 6193.532 | -0.013 | 0.010 |
| 7 | 1 | 6 | 6 | | 6 | 1 | 5 | 5 | 6193.532 | -0.041 | 0.010 |
| 8 | 1 | 8 | 9 | | 7 | 1 | 7 | 8 | 6995.925 | 0.010 | 0.010 |
| 8 | 1 | 8 | 8 | | 7 | 1 | 7 | 7 | 6995.925 | -0.002 | 0.010 |
| 8 | 1 | 8 | 7 | | 7 | 1 | 7 | 6 | 6995.925 | -0.004 | 0.010 |
| 8 | 0 | 8 | 8 | | 7 | 0 | 7 | 7 | 7027.931 | 0.027 | 0.010 |
| 8 | 0 | 8 | 9 | | 7 | 0 | 7 | 8 | 7027.931 | -0.048 | 0.010 |
| 8 | 0 | 8 | 7 | | 7 | 0 | 7 | 6 | 7028.063 | 0.039 | 0.010 |
| 8 | 2 | 7 | 8 | | 7 | 2 | 6 | 7 | 7037.709 | -0.065 | 0.010 |
| 8 | 2 | 7 | 9 | | 7 | 2 | 6 | 8 | 7037.709 | 0.040 | 0.010 |
| 8 | 2 | 7 | 7 | | 7 | 2 | 6 | 6 | 7037.709 | 0.021 | 0.010 |
| 8 | 2 | 6 | 9 | | 7 | 2 | 5 | 8 | 7049.304 | 0.008 | 0.010 |
| 8 | 2 | 6 | 7 | | 7 | 2 | 5 | 6 | 7049.304 | -0.006 | 0.010 |
| 8 | 2 | 6 | 8 | | 7 | 2 | 5 | 7 | 7049.491 | -0.000 | 0.010 |
| 8 | 1 | 7 | 9 | | 7 | 1 | 6 | 8 | 7077.472 | -0.015 | 0.010 |
| 9 | 1 | 9 | 10 | | 8 | 1 | 8 | 9 | 7869.568 | 0.003 | 0.010 |
| 9 | 1 | 9 | 9 | | 8 | 1 | 8 | 8 | 7869.568 | 0.002 | 0.010 |
| 9 | 1 | 9 | 8 | | 8 | 1 | 8 | 7 | 7869.568 | -0.009 | 0.010 |
| 9 | 0 | 9 | 10 | | 8 | 0 | 8 | 9 | 7903.034 | -0.052 | 0.010 |
| 9 | 0 | 9 | 9 | | 8 | 0 | 8 | 8 | 7903.034 | 0.034 | 0.010 |
| 9 | 0 | 9 | 8 | | 8 | 0 | 8 | 7 | 7903.116 | -0.006 | 0.010 |
| 9 | 2 | 8 | 10 | | 8 | 2 | 7 | 9 | 7916.721 | -0.011 | 0.010 |
| 9 | 2 | 8 | 8 | | 8 | 2 | 7 | 7 | 7916.721 | -0.029 | 0.010 |
| 9 | 2 | 8 | 9 | | 8 | 2 | 7 | 8 | 7916.831 | 0.030 | 0.010 |
| 9 | 3 | 7 | 10 | | 8 | 3 | 6 | 9 | 7921.215 | -0.000 | 0.010 |
| 9 | 3 | 7 | 8 | | 8 | 3 | 6 | 7 | 7921.215 | 0.000 | 0.010 |
| 9 | 3 | 7 | 9 | | 8 | 3 | 6 | 8 | 7921.399 | -0.006 | 0.010 |
| 9 | 3 | 6 | 8 | | 8 | 3 | 5 | 7 | 7921.776 | -0.004 | 0.010 |
| 9 | 3 | 6 | 10 | | 8 | 3 | 5 | 9 | 7921.776 | -0.005 | 0.010 |
| 9 | 3 | 6 | 9 | | 8 | 3 | 5 | 8 | 7921.993 | 0.014 | 0.010 |
| 9 | 2 | 7 | 10 | | 8 | 2 | 6 | 9 | 7933.176 | 0.004 | 0.010 |
| 9 | 2 | 7 | 8 | | 8 | 2 | 6 | 7 | 7933.176 | -0.009 | 0.010 |
| 9 | 2 | 7 | 9 | | 8 | 2 | 6 | 8 | 7933.339 | -0.000 | 0.010 |
| 9 | 1 | 8 | 10 | | 8 | 1 | 7 | 9 | 7961.112 | -0.008 | 0.010 |
| 9 | 1 | 8 | 9 | | 8 | 1 | 7 | 8 | 7961.112 | -0.015 | 0.010 |
| 9 | 1 | 8 | 8 | | 8 | 1 | 7 | 7 | 7961.187 | 0.020 | 0.010 |

Table S41: Experimental rotational transition frequencies (MHz) for the #1:4 water complex of #1-DEAcA (aaAs:4w).

| $J'$ | $K_a'$ | $K_c'$ | $F'$ | - | $J''$ | $K_a''$ | $K_c''$ | $F''$ | $\nu_{\text{obs}}$ | $\nu_{\text{obs}} - \nu_{\text{calc}}$ | Err. |
|---|---|---|---|---|---|---|---|---|---|---|---|
| 3 | 0 | 3 | 4 | | 2 | 0 | 2 | 3 | 2275.633 | -0.018 | 0.010 |
| 3 | 0 | 3 | 3 | | 2 | 0 | 2 | 2 | 2275.807 | 0.002 | 0.010 |
| 2 | 2 | 0 | 2 | | 1 | 1 | 0 | 1 | 2714.738 | -0.011 | 0.010 |
| 2 | 2 | 0 | 3 | | 1 | 1 | 0 | 2 | 2714.892 | 0.000 | 0.010 |
| 2 | 2 | 1 | 2 | | 1 | 1 | 1 | 1 | 2753.575 | -0.006 | 0.010 |
| 2 | 2 | 1 | 3 | | 1 | 1 | 1 | 2 | 2754.949 | 0.023 | 0.010 |
| 3 | 1 | 2 | 3 | | 2 | 0 | 2 | 2 | 2822.139 | 0.000 | 0.010 |
| 3 | 1 | 2 | 4 | | 2 | 0 | 2 | 3 | 2823.186 | 0.001 | 0.010 |
| 3 | 1 | 2 | 2 | | 2 | 0 | 2 | 1 | 2823.832 | -0.007 | 0.010 |
| 4 | 1 | 4 | 5 | | 3 | 1 | 3 | 4 | 2959.506 | 0.015 | 0.010 |
| 4 | 0 | 4 | 5 | | 3 | 0 | 3 | 4 | 3018.995 | -0.013 | 0.010 |
| 4 | 0 | 4 | 4 | | 3 | 0 | 3 | 3 | 3019.141 | -0.031 | 0.010 |
| 4 | 1 | 3 | 5 | | 3 | 1 | 2 | 4 | 3131.785 | -0.007 | 0.010 |
| 4 | 1 | 3 | 3 | | 3 | 1 | 2 | 2 | 3131.785 | 0.009 | 0.010 |
| 4 | 1 | 3 | 4 | | 3 | 1 | 2 | 3 | 3131.979 | -0.001 | 0.010 |
| 3 | 2 | 1 | 3 | | 2 | 1 | 1 | 2 | 3448.342 | -0.007 | 0.010 |
| 3 | 2 | 1 | 4 | | 2 | 1 | 1 | 3 | 3448.503 | -0.003 | 0.010 |
| 3 | 2 | 2 | 3 | | 2 | 1 | 2 | 2 | 3560.097 | 0.003 | 0.010 |
| 3 | 2 | 2 | 4 | | 2 | 1 | 2 | 3 | 3561.441 | 0.000 | 0.010 |
| 3 | 2 | 2 | 2 | | 2 | 1 | 2 | 1 | 3562.194 | 0.004 | 0.010 |
| 4 | 1 | 3 | 4 | | 3 | 0 | 3 | 3 | 3678.311 | -0.002 | 0.010 |
| 4 | 1 | 3 | 5 | | 3 | 0 | 3 | 4 | 3679.319 | -0.006 | 0.010 |
| 4 | 1 | 3 | 3 | | 3 | 0 | 3 | 2 | 3679.722 | -0.008 | 0.010 |
| 5 | 1 | 5 | 6 | | 4 | 1 | 4 | 5 | 3694.061 | 0.108 | 0.010 |
| 5 | 0 | 5 | 6 | | 4 | 0 | 4 | 5 | 3751.905 | 0.008 | 0.010 |
| 5 | 0 | 5 | 4 | | 4 | 0 | 4 | 3 | 3751.905 | -0.049 | 0.010 |
| 5 | 0 | 5 | 5 | | 4 | 0 | 4 | 4 | 3752.061 | -0.005 | 0.010 |
| 5 | 1 | 4 | 6 | | 4 | 1 | 3 | 5 | 3906.960 | -0.005 | 0.010 |
| 5 | 1 | 4 | 4 | | 4 | 1 | 3 | 3 | 3907.090 | 0.123 | 0.010 |
| 4 | 2 | 2 | 4 | | 3 | 1 | 2 | 3 | 4179.846 | 0.065 | 0.010 |
| 4 | 2 | 2 | 5 | | 3 | 1 | 2 | 4 | 4179.846 | -0.011 | 0.010 |
| 4 | 2 | 2 | 3 | | 3 | 1 | 2 | 2 | 4179.846 | -0.063 | 0.010 |
| 3 | 3 | 0 | 3 | | 2 | 2 | 0 | 2 | 4299.095 | 0.001 | 0.010 |
| 3 | 3 | 0 | 2 | | 2 | 2 | 0 | 1 | 4299.280 | 0.001 | 0.010 |
| 3 | 3 | 0 | 4 | | 2 | 2 | 0 | 3 | 4299.450 | 0.006 | 0.010 |
| 3 | 3 | 1 | 3 | | 2 | 2 | 1 | 2 | 4302.363 | 0.002 | 0.010 |
| 3 | 3 | 1 | 2 | | 2 | 2 | 1 | 1 | 4302.656 | 0.009 | 0.010 |
| 3 | 3 | 1 | 4 | | 2 | 2 | 1 | 3 | 4302.767 | -0.009 | 0.010 |
| 4 | 2 | 3 | 4 | | 3 | 1 | 3 | 3 | 4387.799 | 0.000 | 0.010 |
| 4 | 2 | 3 | 5 | | 3 | 1 | 3 | 4 | 4388.985 | 0.005 | 0.010 |
| 4 | 2 | 3 | 3 | | 3 | 1 | 3 | 2 | 4389.461 | 0.013 | 0.010 |
| 6 | 1 | 6 | 7 | | 5 | 1 | 5 | 6 | 4425.598 | 0.013 | 0.010 |
| 6 | 1 | 6 | 5 | | 5 | 1 | 5 | 4 | 4425.598 | -0.043 | 0.010 |
| 6 | 1 | 6 | 6 | | 5 | 1 | 5 | 5 | 4425.665 | -0.024 | 0.010 |
| 6 | 0 | 6 | 5 | | 5 | 0 | 5 | 4 | 4475.741 | -0.058 | 0.010 |
| 6 | 0 | 6 | 7 | | 5 | 0 | 5 | 6 | 4475.741 | -0.019 | 0.010 |
| 6 | 2 | 5 | 7 | | 5 | 2 | 4 | 6 | 4563.326 | 0.005 | 0.010 |
| 6 | 2 | 5 | 5 | | 5 | 2 | 4 | 4 | 4563.326 | 0.009 | 0.010 |
| 6 | 2 | 5 | 6 | | 5 | 2 | 4 | 5 | 4563.520 | -0.011 | 0.010 |
| 5 | 1 | 4 | 5 | | 4 | 0 | 4 | 4 | 4566.239 | -0.001 | 0.010 |
| 5 | 1 | 4 | 6 | | 4 | 0 | 4 | 5 | 4567.287 | 0.004 | 0.010 |
| 5 | 1 | 4 | 4 | | 4 | 0 | 4 | 3 | 4567.586 | -0.001 | 0.010 |
| 6 | 2 | 4 | 7 | | 5 | 2 | 3 | 6 | 4666.318 | 0.005 | 0.010 |
| 6 | 2 | 4 | 5 | | 5 | 2 | 3 | 4 | 4666.318 | 0.001 | 0.010 |
| 6 | 2 | 4 | 6 | | 5 | 2 | 3 | 5 | 4666.318 | -0.063 | 0.010 |
| 6 | 1 | 5 | 5 | | 5 | 1 | 4 | 4 | 4675.971 | -0.007 | 0.010 |

Table S41: Experimental rotational transition frequencies (MHz) for the #1:4 water complex of #1-DEAcA (aaAs:4w) *(continued)*

| $J'$ | $K_a'$ | $K_c'$ | $F'$ | - | $J''$ | $K_a''$ | $K_c''$ | $F''$ | $\nu_{\text{obs}}$ | $\nu_{\text{obs}} - \nu_{\text{calc}}$ | Err. |
|---|---|---|---|---|---|---|---|---|---|---|---|
| 6 | 1 | 5 | 7 | | 5 | 1 | 4 | 6 | 4675.971 | -0.004 | 0.010 |
| 6 | 1 | 5 | 6 | | 5 | 1 | 4 | 5 | 4676.099 | 0.003 | 0.010 |
| 5 | 2 | 3 | 5 | | 4 | 1 | 3 | 4 | 4919.922 | -0.001 | 0.010 |
| 5 | 2 | 3 | 6 | | 4 | 1 | 3 | 5 | 4920.002 | -0.009 | 0.010 |
| 5 | 2 | 3 | 4 | | 4 | 1 | 3 | 3 | 4920.002 | -0.055 | 0.010 |
| 4 | 3 | 1 | 4 | | 3 | 2 | 1 | 3 | 5055.933 | 0.000 | 0.010 |
| 4 | 3 | 1 | 5 | | 3 | 2 | 1 | 4 | 5056.331 | 0.013 | 0.010 |
| 4 | 3 | 1 | 3 | | 3 | 2 | 1 | 2 | 5056.331 | -0.069 | 0.010 |
| 4 | 3 | 2 | 4 | | 3 | 2 | 2 | 3 | 5071.899 | -0.000 | 0.010 |
| 4 | 3 | 2 | 5 | | 3 | 2 | 2 | 4 | 5072.451 | 0.005 | 0.010 |
| 4 | 3 | 2 | 3 | | 3 | 2 | 2 | 2 | 5072.581 | -0.004 | 0.010 |
| 5 | 2 | 4 | 5 | | 4 | 1 | 4 | 4 | 5236.454 | -0.001 | 0.010 |
| 5 | 2 | 4 | 6 | | 4 | 1 | 4 | 5 | 5237.530 | 0.005 | 0.010 |
| 5 | 2 | 4 | 4 | | 4 | 1 | 4 | 3 | 5237.851 | 0.002 | 0.010 |
| 9 | 2 | 7 | 9 | | 8 | 3 | 5 | 8 | 5347.044 | -0.027 | 0.010 |
| 9 | 2 | 7 | 10 | | 8 | 3 | 5 | 9 | 5347.044 | -0.039 | 0.010 |
| 9 | 2 | 7 | 8 | | 8 | 3 | 5 | 7 | 5347.044 | -0.050 | 0.010 |
| 6 | 1 | 5 | 6 | | 5 | 0 | 5 | 5 | 5490.264 | -0.006 | 0.010 |
| 6 | 1 | 5 | 7 | | 5 | 0 | 5 | 6 | 5491.370 | 0.009 | 0.010 |
| 6 | 1 | 5 | 5 | | 5 | 0 | 5 | 4 | 5491.606 | -0.005 | 0.010 |
| 6 | 2 | 4 | 6 | | 5 | 1 | 4 | 5 | 5679.200 | -0.005 | 0.010 |
| 6 | 2 | 4 | 5 | | 5 | 1 | 4 | 4 | 5679.360 | -0.049 | 0.010 |
| 6 | 2 | 4 | 7 | | 5 | 1 | 4 | 6 | 5679.360 | 0.001 | 0.010 |
| 5 | 3 | 2 | 5 | | 4 | 2 | 2 | 4 | 5802.209 | 0.001 | 0.010 |
| 5 | 3 | 2 | 6 | | 4 | 2 | 2 | 5 | 5802.467 | 0.021 | 0.010 |
| 5 | 3 | 2 | 4 | | 4 | 2 | 2 | 3 | 5802.467 | -0.042 | 0.010 |
| 5 | 3 | 3 | 5 | | 4 | 2 | 3 | 4 | 5848.073 | -0.001 | 0.010 |
| 5 | 3 | 3 | 6 | | 4 | 2 | 3 | 5 | 5848.589 | 0.003 | 0.010 |
| 5 | 3 | 3 | 4 | | 4 | 2 | 3 | 3 | 5848.717 | -0.004 | 0.010 |
| 4 | 4 | 0 | 4 | | 3 | 3 | 0 | 3 | 5868.401 | 0.006 | 0.010 |
| 4 | 4 | 0 | 3 | | 3 | 3 | 0 | 2 | 5868.573 | 0.033 | 0.010 |
| 4 | 4 | 0 | 5 | | 3 | 3 | 0 | 4 | 5868.573 | -0.067 | 0.010 |
| 4 | 4 | 1 | 4 | | 3 | 3 | 1 | 3 | 5868.573 | 0.010 | 0.010 |
| 4 | 4 | 1 | 5 | | 3 | 3 | 1 | 4 | 5868.786 | -0.025 | 0.010 |
| 4 | 4 | 1 | 3 | | 3 | 3 | 1 | 2 | 5868.786 | 0.074 | 0.010 |
| 8 | 1 | 8 | 9 | | 7 | 1 | 7 | 8 | 5881.047 | 0.022 | 0.010 |
| 6 | 2 | 5 | 6 | | 5 | 1 | 5 | 5 | 6105.913 | 0.018 | 0.010 |
| 6 | 2 | 5 | 7 | | 5 | 1 | 5 | 6 | 6106.905 | 0.011 | 0.010 |
| 6 | 2 | 5 | 5 | | 5 | 1 | 5 | 4 | 6107.156 | 0.020 | 0.010 |
| 8 | 1 | 7 | 7 | | 7 | 1 | 6 | 6 | 6187.486 | 0.007 | 0.010 |
| 8 | 1 | 7 | 9 | | 7 | 1 | 6 | 8 | 6187.486 | 0.006 | 0.010 |
| 8 | 2 | 6 | 7 | | 7 | 2 | 5 | 6 | 6256.845 | 0.013 | 0.010 |
| 8 | 2 | 6 | 8 | | 7 | 2 | 5 | 7 | 6256.845 | -0.007 | 0.010 |
| 8 | 2 | 6 | 9 | | 7 | 2 | 5 | 8 | 6256.845 | 0.019 | 0.010 |
| 7 | 1 | 6 | 7 | | 6 | 0 | 6 | 6 | 6451.277 | -0.007 | 0.010 |
| 7 | 1 | 6 | 8 | | 6 | 0 | 6 | 7 | 6452.444 | 0.033 | 0.010 |
| 7 | 1 | 6 | 6 | | 6 | 0 | 6 | 5 | 6452.598 | -0.026 | 0.010 |
| 7 | 2 | 5 | 7 | | 6 | 1 | 5 | 6 | 6465.815 | -0.007 | 0.010 |
| 7 | 2 | 5 | 6 | | 6 | 1 | 5 | 5 | 6466.077 | -0.045 | 0.010 |
| 7 | 2 | 5 | 8 | | 6 | 1 | 5 | 7 | 6466.077 | 0.010 | 0.010 |
| 6 | 3 | 3 | 5 | | 5 | 2 | 3 | 4 | 6533.649 | -0.087 | 0.010 |
| 6 | 3 | 3 | 6 | | 5 | 2 | 3 | 5 | 6533.649 | 0.033 | 0.010 |
| 6 | 3 | 3 | 7 | | 5 | 2 | 3 | 6 | 6533.649 | -0.056 | 0.010 |
| 9 | 1 | 9 | 10 | | 8 | 1 | 8 | 9 | 6605.548 | -0.001 | 0.010 |
| 5 | 4 | 1 | 5 | | 4 | 3 | 1 | 4 | 6631.740 | -0.006 | 0.010 |
| 5 | 4 | 1 | 6 | | 4 | 3 | 1 | 5 | 6632.065 | -0.029 | 0.010 |

Table S41: Experimental rotational transition frequencies (MHz) for the #1:4 water complex of #1-DEAcA (aaAs:4w) *(continued)*

| $J'$ | $K'_a$ | $K'_c$ | $F'$ | - | $J''$ | $K''_a$ | $K''_c$ | $F''$ | $\nu_{\text{obs}}$ | $\nu_{\text{obs}} - \nu_{\text{calc}}$ | Err. |
|---|---|---|---|---|---|---|---|---|---|---|---|
| 5 | 4 | 1 | 4 | | 4 | 3 | 1 | 3 | 6632.178 | 0.043 | 0.010 |
| 5 | 4 | 2 | 5 | | 4 | 3 | 2 | 4 | 6632.909 | 0.005 | 0.010 |
| 5 | 4 | 2 | 6 | | 4 | 3 | 2 | 5 | 6633.244 | -0.019 | 0.010 |
| 5 | 4 | 2 | 4 | | 4 | 3 | 2 | 3 | 6633.339 | 0.032 | 0.010 |
| 6 | 3 | 4 | 6 | | 5 | 2 | 4 | 5 | 6633.663 | 0.001 | 0.010 |
| 6 | 3 | 4 | 7 | | 5 | 2 | 4 | 6 | 6634.139 | -0.001 | 0.010 |
| 6 | 3 | 4 | 5 | | 5 | 2 | 4 | 4 | 6634.260 | 0.007 | 0.010 |
| 9 | 2 | 8 | 8 | | 8 | 2 | 7 | 7 | 6807.616 | -0.013 | 0.010 |
| 9 | 2 | 8 | 10 | | 8 | 2 | 7 | 9 | 6807.616 | -0.006 | 0.010 |
| 9 | 2 | 8 | 9 | | 8 | 2 | 7 | 8 | 6807.731 | 0.002 | 0.010 |
| 9 | 1 | 8 | 8 | | 8 | 1 | 7 | 7 | 6926.675 | 0.023 | 0.010 |
| 9 | 1 | 8 | 10 | | 8 | 1 | 7 | 9 | 6926.675 | 0.022 | 0.010 |
| 7 | 2 | 6 | 7 | | 6 | 1 | 6 | 6 | 6995.614 | 0.009 | 0.010 |
| 7 | 2 | 6 | 8 | | 6 | 1 | 6 | 7 | 6996.560 | 0.008 | 0.010 |
| 7 | 2 | 6 | 6 | | 6 | 1 | 6 | 5 | 6996.748 | 0.004 | 0.010 |
| 7 | 3 | 4 | 7 | | 6 | 2 | 4 | 6 | 7249.624 | -0.006 | 0.010 |
| 7 | 3 | 4 | 6 | | 6 | 2 | 4 | 5 | 7249.624 | 0.003 | 0.010 |
| 7 | 3 | 4 | 8 | | 6 | 2 | 4 | 7 | 7249.624 | 0.013 | 0.010 |
| 8 | 2 | 6 | 8 | | 7 | 1 | 6 | 7 | 7285.739 | 0.001 | 0.010 |
| 8 | 2 | 6 | 7 | | 7 | 1 | 6 | 6 | 7286.095 | -0.046 | 0.010 |
| 8 | 2 | 6 | 9 | | 7 | 1 | 6 | 8 | 7286.095 | 0.013 | 0.010 |
| 6 | 4 | 2 | 6 | | 5 | 3 | 2 | 5 | 7393.055 | -0.005 | 0.010 |
| 6 | 4 | 2 | 7 | | 5 | 3 | 2 | 6 | 7393.377 | 0.002 | 0.010 |
| 6 | 4 | 2 | 5 | | 5 | 3 | 2 | 4 | 7393.377 | -0.053 | 0.010 |
| 6 | 4 | 3 | 6 | | 5 | 3 | 3 | 5 | 7397.605 | 0.001 | 0.010 |
| 6 | 4 | 3 | 5 | | 5 | 3 | 3 | 4 | 7397.963 | -0.044 | 0.010 |
| 6 | 4 | 3 | 7 | | 5 | 3 | 3 | 6 | 7397.963 | 0.016 | 0.010 |
| 7 | 3 | 5 | 7 | | 6 | 2 | 5 | 6 | 7431.570 | 0.009 | 0.010 |
| 7 | 3 | 5 | 6 | | 6 | 2 | 5 | 5 | 7432.076 | -0.039 | 0.010 |
| 7 | 3 | 5 | 8 | | 6 | 2 | 5 | 7 | 7432.076 | 0.055 | 0.010 |
| 10 | 1 | 9 | 11 | | 9 | 1 | 8 | 10 | 7654.537 | 0.019 | 0.010 |
| 10 | 1 | 9 | 9 | | 9 | 1 | 8 | 8 | 7654.537 | 0.021 | 0.010 |
| 8 | 3 | 5 | 9 | | 7 | 2 | 5 | 8 | 7955.192 | 0.015 | 0.010 |
| 8 | 3 | 5 | 8 | | 7 | 2 | 5 | 7 | 7955.192 | -0.059 | 0.010 |
| 8 | 3 | 5 | 7 | | 7 | 2 | 5 | 6 | 7955.192 | 0.015 | 0.010 |
